# Acetoacetate–GPR43 axis epigenetically silences IL-6/CSF1 to restrict TAMs-driven metastatic lung cancer

**DOI:** 10.1186/s12967-026-08742-6

**Published:** 2026-08-03

**Authors:** Shuying Yuan, Biying Xiao, Shuaishuai Ni, Wei Liu, Mirong Hou, Yanyu Jiang, Lijun Jia

**Affiliations:** 1 Cancer Institute of Traditional Chinese Medicine, Longhua Hospital, Shanghai University of Traditional Chinese Medicine, Shanghai, 200032 China; 2 School of Integrative Medicine, Nanjing University of Chinese Medicine, Nanjing, 210023 China

**Keywords:** Acetoacetate, Metastatic lung cancer, GPR43, Tumor-associated macrophages (TAMs), DNA hypermethylation, IL-6, CSF1

## Abstract

**Background:**

Metastasis remains the principal cause of cancer mortality, with the lungs representing one of the most frequent and clinically challenging sites. The tumor microenvironment, particularly tumor-associated macrophages (TAMs), plays a pivotal role in establishing and maintaining metastatic niches. Yet, how host ketone bodies influence the immune microenvironment to govern metastatic progression is poorly defined.

**Methods:**

Pan-cancer gene expression analysis on the Home for Researchers platform to assess the activity of enzymes involved in acetoacetate synthesis and catabolism. Two murine metastatic Lung Cancer models were established to evaluate the role of acetoacetate in metastatic progression. Flow cytometry and immunohistochemistry were used to assess immune cell infiltration, particularly TAMs. Molecular modeling, Drug Affinity Responsive Target Stability, Cellular Thermal Shift Assay, receptor activation assays, inhibitor and small interfering RNA experiments were used to examine the interaction between acetoacetate and the G protein-coupled receptor 43 (GPR43). Immunofluorescence, RNA sequencing, quantitative PCR, Transwell, cytokine supplementation, dot and western blotting, methylated DNA immunoprecipitation–qPCR, and enzyme-linked immunosorbent assay were performed to investigate how acetoacetate regulates interleukin-6 (IL-6) and colony-stimulating factor 1 (CSF1), as well as the underlying epigenetic mechanisms.

**Results:**

We uncover a previously unappreciated collapse of acetoacetate homeostasis in lung cancer, driven by the coordinated suppression of ketogenesis and increased ketolysis, resulting in a systemic acetoacetate deficiency. Restoring acetoacetate significantly limits metastatic lung cancer spread and reduces TAM infiltration within the tumor microenvironment. Mechanistically, we identify acetoacetate as an endogenous ligand for GPR43, linking metabolic sensing to immune regulation. Disruption of GPR43 signaling abolishes the anti-metastatic effects of acetoacetate, confirming a reliance on this pathway. Strikingly, acetoacetate–GPR43 signaling increases methionine adenosyltransferase 2A (MAT2A) and S-adenosylmethionine levels and induces region-specific DNA hypermethylation of pro-inflammatory cytokine genes, including IL6 and CSF1, thereby silencing their transcription. Restoring IL-6 and CSF1 reestablishes TAMs’ proliferation and migration, reactivating a pro-metastatic environment.

**Conclusions:**

Our findings reveal a novel “metabolic–epigenetic–immune” axis, in which acetoacetate, through GPR43 signaling, reprograms the monocyte epigenome via DNA hypermethylation to suppress pro-metastatic inflammation. This study identifies the acetoacetate–GPR43 axis as a mechanistically grounded, potential therapeutic strategy for metastatic lung cancer and offers new insights into overcoming the limitations of current TAM-targeted therapies.

**Supplementary information:**

The online version contains supplementary material available at 10.1186/s12967-026-08742-6.

## Introduction

Metastasis remains the leading cause of cancer-related death, responsible for over 90% of fatalities in patients with solid tumors [1]. The lung is among the most susceptible organs to both primary and metastatic malignancies due to their role in filtering systemic venous return and their extensive vascular network [2]. Despite progress in targeted therapies and immunotherapies [3], effective strategies to prevent or treat metastatic lung cancer remain limited. Increasing evidence emphasizes the crucial role of the tumor microenvironment (TME) in supporting metastatic progression [4]. Among various immune cells, tumor-associated macrophages (TAMs) are significant contributors [5]. TAMs promote angiogenesis, remodel the extracellular matrix, inhibit cytotoxic immune responses, and release cytokines that create a pro-metastatic environment [6]. In particular, interleukin-6 (IL-6) and colony-stimulating factor-1 (CSF1) are key mediators of monocyte recruitment [7], TAMs proliferation and differentiation, and immunosuppressive polarization [8, 9]. High levels of IL-6 and CSF1 are associated with poor prognosis and therapy resistance across different cancer types [10, 11]. We have long tried to block these cytokines with drugs, ignoring the possibility that environmental metabolites may act as their natural regulators.

Metabolic interventions have gained renewed attention as potential adjunct therapies for cancer. Among these, the ketogenic diet (KD)—a low-carbohydrate, high-fat nutritional plan—has been proposed to reprogram tumor metabolism and modulate immune responses [12–14]. Ketone bodies, including β-hydroxybutyrate (BHB) and acetoacetate, are the main metabolites produced under KD or fasting conditions [15]. Traditionally viewed as alternative energy substrates, ketone bodies are increasingly recognized as signaling molecules that can modulate inflammation, chromatin accessibility, and cellular stress responses [12]. For instance, BHB has been shown to inhibit histone deacetylases, affecting FOXO3A and MT2 gene expression and reducing oxidative stress [16]. Although BHB has been widely studied, prolonged use may lead to metabolic disorders such as ketoacidosis and hepatorenal toxicity [17, 18]. Acetoacetate, the second most abundant ketone body in circulation, remains poorly characterized with respect to its systemic biological effects, particularly in metastatic lung cancer. Previous research indicated that acetoacetate selectively promoted the progression of BRAF V600E-positive melanoma by enhancing BRAF V600E-MEK1 binding [19, 20]. However, it has not been systematically studied whether acetoacetate has tissue-specific effects or acts as a signaling metabolite that influences immune cell epigenetic programs and inflammatory levels in the TME.

In this study, we uncover that acetoacetate acts as a signaling metabolite that suppresses metastatic lung cancer by directly engaging the G protein–coupled receptor GPR43, thereby elevating MAT2A and intracellular S-adenosylmethionine levels, which induce region-specific DNA hypermethylation at IL6 and CSF1, resulting in transcriptional silencing and reduced TAM infiltration. These results revealed a crucial perspective that acetoacetate reprograms the monocyte epigenome, linking nutrient sensing to immune regulation and breaking through the traditional limitation of focusing solely on tumor cells’ competition for nutrients and their own metabolism.

## Materials and methods

### Cell culture

The Mouse LLC (RRID: CVCL_4358), CMT167 (RRID: CVCL_2405), 4T1 (RRID: CVCL_0125), and RAW264.7 (RRID: CVCL_0493) cell lines, as well as the human THP1 (RRID: CVCL_0006) cell line, were obtained from the Type Culture Collection of the Chinese Academy of Sciences (Shanghai, China). LLC, CMT167, 4T1, and THP1 were cultured in Roswell Park Memorial Institute medium (RPMI 1640, BasalMedia, China); RAW264.7 was cultured in Dulbecco’s Modified Eagle’s Medium (DMEM, BasalMedia, China). All media contained 10% fetal bovine serum (FBS, Biochrom AG, Germany) and 1% penicillin-streptomycin solution (Gibco, United States). Cells were incubated at 37 °C in a humidified atmosphere with 5% CO₂ and were free of contamination.

Primary mouse peritoneal macrophages (PM) were isolated by peritoneal lavage. Eight-week-old C57BL/6J mice were injected intraperitoneally with 2 mL of 3% Brewer thioglycolate medium 3 days before sacrifice to elicit the recruitment of macrophages. After euthanasia, an appropriate volume of sterile PBS was injected into the peritoneal cavity, and the abdomen was gently massaged. The lavage fluid was collected and centrifuged at 500 × g for 10 min at 4 °C. The cell pellet was resuspended in RPMI 1640 medium and cultured.

### Reagents

Acetoacetic acid sodium (AC, HY-112540B, MCE), Nicotinamide (NAM, HY-B0150, MCE), LPS (tlrl-b5lps, InvivoGen), SAM (S914828, Macklin) were dissolved in H_2_O; IL-6 (216–16, Thermo Fisher Scientific), M-CSF/CSF1 (HY-P7085, MCE) were dissolved in 0.1% BSA; GLPG0974 (HY-12940, MCE), CATPB (HY-116263, MCE), 5-Azacytidine (5-AZAC, HY-10586, MCE), Decitabine (DTB, HY-A0004, MCE), Trichostatin A (TSA, HY-15144, MCE), BRD4770 (BRD, HY-16705, MCE), BIX-01294 (BIX, HY-10587, MCE), PF-9366 (PF, HY-107778, MCE), SCH772984 (SCH, HY-50846, MCE) were dissolved in dimethyl sulfoxide (DMSO, Sigma-Aldrich, Germany). Additionally, GLPG0974 was first dissolved in 10% DMSO, then diluted with 40% PEG300, followed by 5% Tween-80, and finally adjusted to 45% saline for the in vivo study.

### Animal tumor model

Five-week-old female Balb/c and C57BL/6 mice were purchased from GemPharmatech Biological Technology (Jiangsu, China). These mice were housed and treated according to established guidelines, with the protocol approved by the Institutional Animal Care and Use Committee of Longhua Hospital, Shanghai University of Traditional Chinese Medicine (LHERAW-20012). In Balb/c mice, 4T1 breast cancer cells were implanted subcutaneously to create an in situ breast cancer metastatic lung cancer model. LLC lung cancer cells were injected into the tail vein to establish a tail-vein metastatic lung cancer model in C57BL/6 mice. Tumor size was measured with a vernier caliper and calculated as length × width. The body weights of the mice, along with their food and water intake, were recorded every other day using an electronic scale. All mice were kept in specific pathogen-free (SPF) conditions. All procedures conformed to the National Institutes of Health Guide for the Care and Use of Laboratory Animals.

### Quantification of acetoacetate content

The acetoacetate content in lung and tumor tissues and in the serum of mice was measured using the Acetoacetate Content Assay Kit (Boxbio, China) according to the manufacturer’s instructions.

### Flow cytometry

For analysis of different cell populations, lung tissues were digested with collagenase IV (1 mg/ml, C5138, Sigma) and DNase I (1 mg/ml, 10104159001, Roche) to produce single-cell suspensions. These suspensions were then analyzed by FACS (DxFLEX, Beckman Colter). The following fluorochrome-labeled anti-mouse antibodies were used: L/D-FVS780, CD45–BV510, CD3e-AF700, CD4-FITC, CD8–AF647, NK1.1-PE-CF594, CD11B-BB515, F4/80-BV421, CD86-PE, CD206–AF647, Ki-67-BV650, LY-6 G-BV786, LY-6C-BV605, CD11C-PE-CY7; Fc Block (BD Pharmingen). Data were processed and analyzed using FlowJo version 10.8.1. Cells identified as CD11B^+^F4/80^+^ were classified as tumor-associated macrophages (TAMs). Additionally, when the distinction between positive and negative cell populations was unclear, a fluorescence-minus-one (FMO) control was used to ensure accurate categorization and subsequent analysis.

### H&E and immunohistochemical staining

Freshly dissected mouse lung, liver, kidney, heart, and spleen tissues were fixed in 4% paraformaldehyde buffer (Beyotime, China), embedded in paraffin, and sectioned. For hematoxylin and eosin (H&E) staining, paraffin sections were heated at 60 °C for 1 h, and slides were deparaffinized with dimethylbenzene and a series of ethanol concentrations. Sections were stained with hematoxylin, differentiated with 1% hydrochloric acid in ethanol, and rinsed in water for bluing. They were then stained with eosin, dehydrated through graded ethanol, cleared in xylene, and mounted with neutral resin.

For immunohistochemical (IHC) analysis [21–24], deparaffinized and rehydrated sections underwent antigen retrieval in EDTA buffer and were blocked with goat serum. Sections were incubated overnight at 4 °C with primary antibodies against Ki-67 (ZuochengBio, China) or F4/80 (Cell Signaling Technology, USA). After washing, sections were incubated with appropriate secondary antibodies, and signals were visualized using 3,3′-diaminobenzidine (DAB). Sections were then counterstained with hematoxylin, dehydrated, cleared in xylene, and mounted with neutral resin. ImageJ was used to perform statistical analysis on more than three randomly selected fields of view from the IHC results.

### Drug Affinity Responsive Target Stability (DARTS) Assay

RAW264.7 cells were lysed in RIPA Lysis Buffer (Beyotime, China). The cell lysate was evenly distributed among several tubes and incubated with acetoacetate (25, 50, or 100 mM) or an equal volume of H_2_O for 2 hours on a rotator at 4 °C. After incubation, the mixture was divided into 100 μL aliquots and digested with pronase (1 mg/mL) at various doses at room temperature for 30 minutes. Digestion was stopped by adding SDS-PAGE loading buffer, and the samples were then analyzed by Western blot.

### Cellular Thermal Shift Assay (CETSA)

RAW264.7 cells were lysed in RIPA Lysis Buffer (Beyotime, China) containing freshly added protease and phosphatase inhibitors. Briefly, the cell lysate was evenly distributed among several tubes and incubated with acetoacetate (25, 50, or 100 mM) or an equal volume of H_2_O for 2 hours on a rotator at 4 °C. After incubation, the mixture was divided into 100 μL aliquots in tubes and heated for 3 minutes at the specified temperature. After cooling for 3 minutes on ice, centrifuge at 12,000 rpm for 10 minutes at 4 °C, then remove the supernatant. Add SDS-PAGE loading buffer, then analyze the samples by Western blot.

### Fluo-4 Calcium Assay

RAW264.7 cells were treated with acetoacetate at indicated concentrations (0, 5, 10, 20, 30, 40, and 50 mM) for 24 hours. After treatment, cells were harvested and processed according to the manufacturer’s instructions of the Fluo-4 Calcium Assay Kit (Beyotime, S1061S). Flow cytometric analysis was performed using a DxFLEX Flow Cytometer (Beckman, United States), and the data were analyzed with FlowJo software version 10.8.1.

### RNA-seq and GO analysis

Mouse lung tissues and RAW264.7 cells were treated with acetoacetate, and total RNA was extracted using the Ultrapure RNA Kit (Vazyme Biotech, China) for RNA-Seq analysis. Differentially expressed genes were identified based on filtering criteria: Log2FC > 0.585 and FDR < 0.05, or Log2FC < −0.585 and FDR < 0.05. Gene Ontology (GO) enrichment analysis was performed using the DAVID tool (https://david-d.ncifcrf.gov/), with a significance threshold of *p* < 0.05. The results of the enrichment analysis were visualized as bubble charts.

### Quantitative real-time PCR (qPCR)

Total RNA was extracted using the Ultrapure RNA Kit according to the manufacturer’s instructions. Then, the PrimerScript reverse transcription reagent kit (Vazyme Biotech, China) was used to synthesize cDNA from the isolated total RNA. Quantitative real-time PCR was carried out with the Power SYBR Green PCR MasterMix (Vazyme Biotech, China) on an ABI 7500 thermocycler (Applied Biosystems, United States), following the manufacturer’s guidelines. As an internal control, β-Actin levels were measured alongside those of the target genes. Normalization and fold change for each gene were calculated using the 2^(-∆∆CT) method. The murine primer sequences used were as follows in Table 1, and the human primer sequences used were as follows in Table 2.

**Table 1:** Murine primers for qPCR

Gene	Forward primers (5’-3’)	Reverse primers (5’-3’)
β-actin	CCAGCCTTCCTTCTTGGGTATG	TGTGTTGGCATAGAGGTCTTACG
ARG-1	CCTGGCCTTTGTTGATGTCC	CCAGAGATGCTTCCAACTGC
PDGF-A	TCGAAGTCAGATCCACAGCA	CTCGGGCACATGGTTAATGG
PDGF-C	AGTGTCCATACGGGAAGAGC	CGTGGGACACACTGACATTC
MMP2	ACTCCGGAGATCTGCAAACA	ACTGTCCGCCAAATAAACCG
MMP9	CCTGTGTGTTCCCGTTCATC	AGGGCAGAAGCCATACAGTT
IL-6	CTGCAAGAGACTTCCATCCAG	AGTGGTATAGACAGGTCTGTTGG
CSF1	TTGCCAAGGAGGTGTCAGAA	CCAGCTGTTCCTGGTCTACA
GPR43	TGTGCACATCCTCCTGCTTA	GGTAGGTACCAGCGGAAGTT

**Table 2:** Human primers for qPCR

Gene	Forward primers (5’-3’)	Reverse primers (5’-3’)
β-actin	TGACGTGGACATCCGCAAAG	CTGGAAGGTGGACAGCGAGG
IL-6	CAGCCCTGAGAAAGGAGACA	CCAGGCAAGTCTCCTCATTG
CSF1	GCAAGAACTGCAACAACAGC	GTTGCAATCAGGCTTGGTCA

### siRNA knockdown experiment

RAW264.7 cells were transfected with siRNA oligonucleotides using CALNPTMRNAi (D-Nano, Beijing) following the manufacturer’s instructions. The siRNA sequences were synthesized by GenePharma (Shanghai, China).

### Multiplex fluorescence staining

First, paraffin sections were dewaxed and hydrated, then antigen retrieval was performed using heat-induced epitope retrieval (HIER) for 20 minutes, followed by inactivation with 3% hydrogen peroxide for 10 minutes. Next, the sections were blocked with serum for 30 minutes to reduce non-specific binding. The primary antibody was incubated overnight at 4 °C. The following day, the HRP-conjugated secondary antibody was incubated at room temperature for 30 minutes, then the TSA-AF fluorescence working solution was applied and incubated in the dark at room temperature for 15 minutes. The same procedure was used to stain another primary antibody and its corresponding secondary antibody. Finally, cell nuclei were counterstained with DAPI for 5 minutes, and an anti-fading fluorescence mounting medium was applied for coverslipping. The antibodies used were FFAR2 (Proteintech, diluted 1:100) and CD163 (ZuoChengBio, diluted 1:400). Imaging was performed using a fluorescence confocal microscope (Leica, Germany).

### Cell viability assay

Cell viability was measured using the ATPlite assay kit (PerkinElmer, United States) according to the manufacturer’s instructions. Cells were seeded into 96-well plates at a density of 1 × 10^4 cells per well in triplicate and cultured overnight under standard conditions. The next day, cells underwent the treatments specified for each experimental condition and were then incubated at 37 °C for 24 hours. Control cells received 0.1% BSA. Finally, cell viability was determined using a microplate reader (BIOTEK Synergy HT, USA).

### Transwell migration assay

Monocyte chemotaxis in response to the addition of IL-6 and CSF1 conditioned medium was assessed using a polycarbonate filter with an 8-μm pore size in 24-well Transwell chambers (Corning, United States). The upper chamber contained 2 × 10^5 RAW264.7 monocytes in serum-free DMEM medium. The lower chambers contained a concentration gradient of IL-6 and CSF1-conditioned medium. After incubation at 37 °C for 24 hours, the migrated cells were fixed with 4% paraformaldehyde (30–60 minutes) and stained with 0.1% crystal violet (30 minutes) (Beyotime, China). Images were captured using the All-in-One multifunctional high-resolution fluorescence microscopy system (KEYENCE, Japan), and the migrated cells were quantified using ImageJ.

### ELISA array

ELISA assays for murine IL-6, CSF1 (both from LIANKE, China), and murine SAM (Lengeton, China) were performed according to the manufacturers’ protocols. In vivo, RIPA lysates from mouse lung tissues were analyzed using the same ELISA kit. In vitro, the lysate of RAW264.7 cells, prepared by repeated freeze-thaw cycles in PBS with liquid nitrogen, was assessed using the SAM ELISA kit.

### Western blot analysis

After the indicated treatments, RAW264.7 cells were harvested. Additionally, nuclear and cytoplasmic extracts were prepared using NE-PER™ Nuclear and Cytoplasmic Extraction Reagents (Thermo Fisher Scientific, United States), while histones were extracted with the Histone Extraction Kit (Abcam, United States). Protein concentrations were then measured using a BCA kit (Epizyme, China). Equal amounts of protein were separated by SDS-PAGE (Epizyme, China) and transferred onto Immobilon PVDF membranes (Merck Millipore Ltd, Ireland). The membranes were blocked with 5% non-fat milk for 1 hour at room temperature, then incubated with primary antibodies overnight at 4 °C. Subsequently, the membranes were incubated with horseradish peroxidase (HRP)-conjugated secondary antibody (Cell Signaling Technology, United States) for 1 hour at room temperature. Protein signals were detected using an ECL reagent (Epizyme, China) and visualized with the Tanon 5200 imaging system (Tanon, China). The primary antibodies used included: HIF1α, IKBα, p-IKBα, P65, p-P65, p-JNK, p-c-Jun, LaminA/C, H3K27me3, H3K27ac, H3K18ac, H3K9ac, H3, DNMT1 (Cell Signaling Technology, United States); FFAR2/GPR43, DNMT3A, DNMT3B, MAT2A (Proteintech, United States); TET2 (Abcam, UK); p-STAT3 (ABclonal, China); p-TAK1 (Abmart, China); p44/42 MAPK (Erk1/2), Phospho-p44/42 MAPK (Erk1/2) (T202/Y204) (Selleck, USA); β-Tubulin (Cwbiotech, China); and β-actin (HuaBio, China).

### Global quantification of 5-mC and 5-hmC using FACS assay

After the indicated treatments, RAW264.7 cells were harvested and resuspended in FACS buffer (PBS with 1% BSA and 2 mM EDTA). The cells were then incubated with an Fc blocker for 10 minutes on ice to reduce nonspecific antibody binding. Following a wash with FACS buffer, the cells were fixed and permeabilized using a Cell Fixation/Permeabilization Kit (Thermo Fisher Scientific, United States) according to the manufacturer’s instructions. Next, the samples were treated with 2 N HCl for 20 minutes and then neutralized with 10 mM Tris–HCl (pH 8.0) for 20 minutes. For immunostaining, primary antibodies against 5-mC (Abcam, diluted 1:200) or 5-hmC (Active Motif, diluted 1:200) were incubated for 1 hour, followed by incubation with the secondary antibody, YSFluor™ 488 Goat Anti-Rabbit IgG (Yeasen Biotech, diluted 1:200), for an additional hour. Flow cytometric analysis was performed using a DxFLEX Flow Cytometer (Beckman, United States), and the data were analyzed with FlowJo software version 10.8.1.

### Global quantification of 5-mC and 5-hmC using the dot blot assay

Genomic DNA was extracted using the Ultrapure Genomic DNA Kit following the manufacturer’s instructions (Vazyme Biotech, China). Next, the DNA was spotted onto a nitrocellulose membrane (GE Whatman, UK). The membrane was exposed to ultraviolet light for 30 minutes to cross-link the DNA. It was then blocked with 5% non-fat milk in TBS-Tween 20 for 1 hour at room temperature, followed by overnight incubation with primary antibodies against 5-mC or 5-hmC at 4 °C. Afterward, the membrane was incubated with an HRP-conjugated secondary antibody (Cell Signaling Technology) for 1 hour at room temperature. The membrane was washed three times with TBS-Tween 20, then scanned with the Tanon 5200 imaging system. Finally, the membrane was stained with a 0.1% methylene blue solution (Solarbio, China) and photographed.

### Methylated DNA immunoprecipitation (MeDIP)-qPCR analysis

Firstly, genomic DNA (2 μg) was collected and sonicated in two cycles: the first at 100 W for 15 repetitions (15 seconds on, 10 seconds off), and the second at 200 W for 15 repetitions (10 seconds on, 10 seconds off). Next, the DNA was denatured at 95 °C for 10 minutes and then cooled on ice for 5 minutes. Immunoprecipitation was performed overnight with an anti-5-mC antibody (Abcam). The antibody–DNA complexes were captured by protein G beads (Thermo Fisher Scientific, United States) for 4 hours. Then, the beads were washed three times and treated with proteinase K (Vazyme Biotech) at 65 °C for 4 hours to release the DNA. Finally, the DNA was purified using a PCR Clean-up Kit (MACHEREY-NAGEL, Germany) and analyzed by qPCR. To evaluate methylation of target genes, it can be calculated using the following formula: % (MeDIP/Total input) = 2^[(Ct (input) - Ct (IP)) × 100]. The primers are as follows in Table 3.

**Table 3:** The primers for MeDIP-qPCR

Gene	Forward primers (5’-3’)	Reverse primers (5’-3’)
IL-6 (−1000 bp to −350 bp)	AGGGAGTGTGTGTTTTTGTATG	ACTTAAATCATCCTTAAAACTAACC
IL-6 (+43 bp to + 403 bp)	TTTGTAAGTAAGTGAAGGTAGTTT	CACAACCTACCCACCTCTTTTCTC
CSF1 (+255 bp to + 439 bp)	TGCACAGCCACAGAGCG	TGGTTTATGGGAAATCACCCT
CSF1 (+552 bp to + 722 bp)	TGCATCCCAGGACAGCG	CGGTTGCAGCTTACCGAAG

### Statistical analysis

All data are presented as mean ± standard error of the mean (SEM) or mean ± standard deviation (SD). Statistical comparisons between groups were performed using GraphPad Prism 9.2.0 (GraphPad Software, Inc., USA). For two‑group comparisons, the two‑tailed unpaired Student’s *t*‑test was used. For multiple comparisons, one‑way ANOVA followed by Dunnett’s post hoc test was generally applied when variances were homogeneous, as assessed by the Brown–Forsythe test. When variances were unequal, Welch’s ANOVA with Dunnett‑T3 post hoc test was employed. Where small sample sizes and considerable data dispersion led to unequal variances across groups, we opted for the Kruskal–Wallis test followed by Dunn’s multiple‑comparison test. This non‑parametric approach does not rely on normality or variance homogeneity, making it more appropriate for these highly variable datasets. The applied statistical analysis methods for each result can be found in the respective figure legends. Significance was defined at four levels: * *p* < 0.05, ** *p* < 0.01, *** *p* < 0.001, and n.s., indicating no significance. Cell‑based experiments were performed in three independent biological replicates, each containing technical triplicates. For animal experiments, details on sample size and randomization are provided in the respective figure legends.

## Result

### Clinical analyses reveal a collapse of acetoacetate homeostasis in metastatic lung cancer, while its restoration suppresses metastatic dissemination

**Fig. 1:**
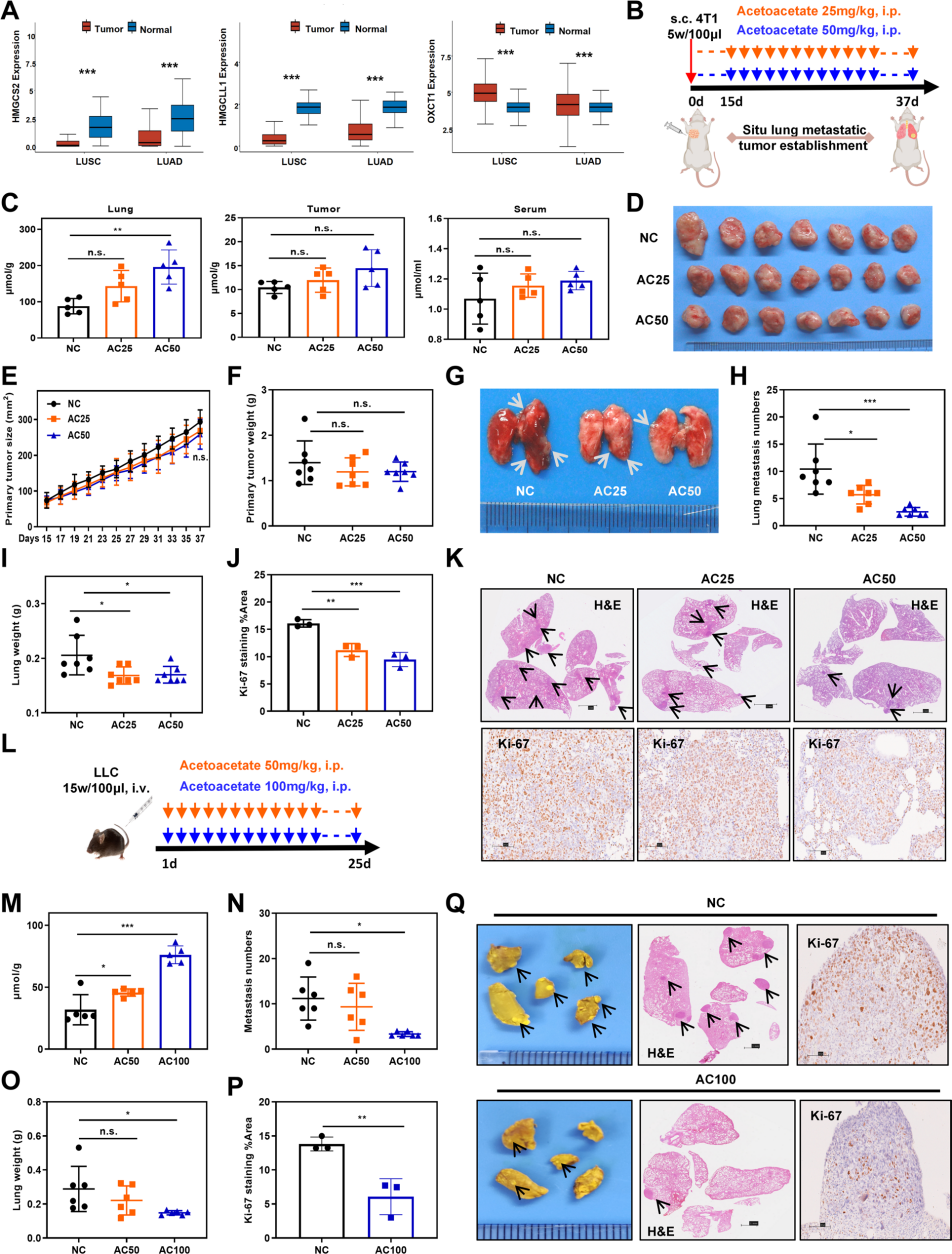
Clinical analyses reveal a collapse of acetoacetate homeostasis in metastatic lung cancer, while its restoration suppresses metastatic dissemination. (**A**). A box plot illustrating the miRNA expression levels of the HMGCS2, HMGCLL1, and OXCT1 genes in tumor and normal tissues from the TCGA and GTEx dataset was generated using the *Wilcoxon* test. (**B**). 5 × 10^4 4T1 breast cancer cells were subcutaneously injected (s.c.) into the right third mammary fat pad of Balb/c mice to establish a metastatic lung cancer model originating from an in-situ tumor. On the 15th day after implantation, mice were randomly assigned to three groups (*n* = 7 per group): negative control (NC), low-dose acetoacetate (AC 25 mg/kg), and high-dose Acetoacetate (AC 50 mg/kg). Acetoacetate was administered daily via intraperitoneal injection (i.p.). (**C**). On day 37, mice were euthanized, and acetoacetate levels in lung and tumor tissues and in serum were measured and analyzed statistically. (**D**). Primary tumors were collected and photographed. (**E**). Tumor growth was monitored following acetoacetate treatment. (**F**). Tumor weights were recorded at sacrifice. (**G**). Lung tissues were harvested and photographed. (**H-I**). Statistical analyses were performed on the number of metastatic lung tumors and lung weights in mice. (**J-K**). Lung tissues were fixed and stained with H&E and Ki-67 IHC. The representative staining images are shown in (**K**). The Ki-67 staining results from randomly selected biological replicates are shown in (**J**). (**L**). A schematic diagram of the in vivo experiment: C57BL/6 mice were intravenously injected with 1.5 × 10^5 LLC lung cancer cells via the tail vein. On the second day post-injection, mice were randomly divided into three groups (*n* = 6 per group): negative control (NC), low-dose acetoacetate (AC 50 mg/kg), and high-dose Acetoacetate (AC 100 mg/kg). Acetoacetate was administered daily via intraperitoneal injection (i.p.). (**M**). Acetoacetate levels in mouse lung tissue were measured and statistically analyzed. (**N-O**). Statistical analyses of metastatic lung tumors and lung weight in mice. (**P-Q**). Lung tissues were collected after fixation and H&E staining, then subjected to Ki-67 IHC staining. The representative staining images are shown in (**Q**). The Ki-67 staining results from randomly selected biological replicates are shown in (**P**). Values are presented as mean ± SD, with * *p* < 0.05, ** *p* < 0.01, *** *p* < 0.001, and n.s. indicating no significance, determined by one-way ANOVA (**C-M**), Welch’s one-way ANOVA (**N**), Kruskal–Wallis test (**O**), and Student’s *t*-test (**P**)

To examine the clinical significance of acetoacetate metabolism in tumors, we analyzed the expression of key acetoacetate-synthesizing enzymes (HMGCS2 and HMGCLL1) and the catabolic enzyme (OXCT1) [12] using the Home for Researchers platform. Both HMGCS2 and HMGCLL1 were notably downregulated, while OXCT1 was significantly upregulated in lung tumor tissues compared to adjacent normal tissues (Fig. 1A), indicating a metabolic reprogramming that diminishes endogenous acetoacetate production during tumor progression.

To clarify the role of acetoacetate in primary tumor growth and metastatic lung tumors, we established an orthotopic breast cancer metastatic lung cancer model by injecting 4T1 breast cancer cells into the mammary fat pad of mice and then assessed its anti-growth and anti-metastatic effects (Fig. 1B). Quantitative analysis showed that acetoacetate selectively accumulated in lung tissues rather than in primary tumors or serum, indicating its targeted enrichment in the lungs (Fig. 1C). While acetoacetate had minimal effects on primary tumor growth (Fig. 1D–F), it significantly reduced metastatic lung tumors burden in a dose-dependent manner (Fig. 1G–I, K). Consistently, Ki67 immunostaining confirmed that acetoacetate markedly inhibited the proliferation of metastatic lesions in lung tissues (Fig. 1J–K). No adverse effects were observed on body weight, food or water intake, or the weight and morphology of major organs—including the liver, kidney, heart, and spleen—supporting the safe profile of acetoacetate for in vivo use (Fig. S1A-D). Overall, these findings demonstrate that acetoacetate is an effective, well-tolerated ketone-body metabolic intervention that targets the lungs and suppresses metastatic lung cancer progression.

To further assess the anti-metastatic effects of acetoacetate, we established a metastatic lung cancer model using tail-vein injection of Lewis lung carcinoma (LLC) cells (Fig. 1L). Quantitative analysis showed a significant increase in acetoacetate levels in lung tissues compared with the control group (Fig. 1M). Consistently, counting metastatic nodules and measuring lung weight indicated that acetoacetate markedly inhibited metastatic lung tumors (Fig. 1N–O, Q). Ki-67 immunohistochemical staining further confirmed that acetoacetate reduced proliferative activity in metastatic lesions (Fig. 1P–Q). Additionally, there were no significant differences in body weight, food intake, or water consumption across the treatment groups (Fig. S1E-F). Weight and histological evaluations of major organs—including the liver, kidney, heart, and spleen—showed no detectable abnormalities (Fig. S1G-H), supporting its potential as a safe and effective metabolic strategy against metastatic lung cancer progression.

### Acetoacetate alters the immune environment to promote an anti-metastatic state

**Fig. 2:**
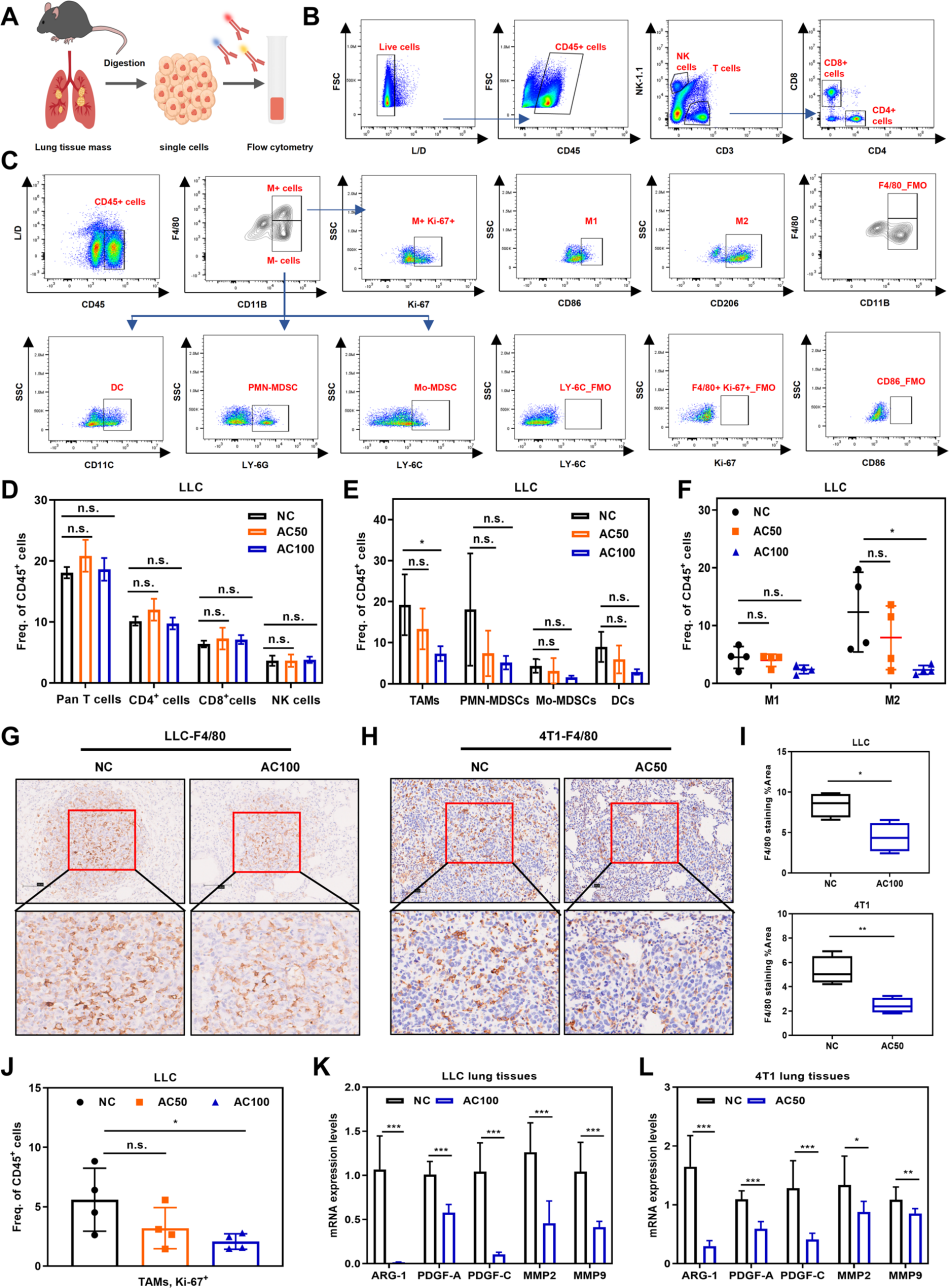
Acetoacetate alters the immune environment to promote an anti-metastatic state. (**A-C**). Flow cytometry was used to identify antibody-labeled immune cells within mouse lung tissues. A schematic illustration of the FlowJo analysis workflow is shown. Pan T cells (CD45^+^ CD3^+^), CD4^+^ T cells (CD45^+^ CD3^+^ CD4^+^ CD8^-^), CD8^+^ T cells (CD45^+^ CD3^+^ CD4^-^ CD8^+^), NK cells (CD45^+^ CD3^-^ NK-1.1^+^), TAMs (CD45^+^ CD11B^+^ F4/80^+^), PMN-MDSCs (CD45^+^ CD11B^+^ F4/80^-^ LY6G^+^), Mo-MDSCs (CD45^+^ CD11B^+^ F4/80^-^ LY6C^+^), DCs (CD45^+^ CD11B^+^ F4/80^-^ CD11C^+^), M^+^ Ki-67^+^ (F4/80^+^ Ki-67^+^), M1 cells (F4/80^+^ CD86^+^), and M2 cells (F4/80^+^ CD206^+^). F480_FMO (CD45^+^ CD11B^+^ F4/80^-^), LY6C_FMO (CD45^+^ CD11B^+^ F4/80^-^ LY6C^-^), Ki-67_FMO (CD45^+^ CD11B^+^ F4/80^+^ Ki-67^-^), and CD86_FMO (CD45^+^ CD11B^+^ F4/80^+^ CD86^-^). (**D-E**). Changes in Pan T cells, CD4^+^ T cells, CD8^+^ T cells, NK cells, TAMs, PMN-MDSCs, Mo-MDSCs, and DCs in NC, low-dose, and high-dose acetoacetate-treated LLC tumor model lung tissues were analyzed. (*n* = 4 independent biological replicates). (**F**). The cellular changes in M1 and M2 phenotypes in lung tissues from an LLC tumor model treated with low- and high-dose acetoacetate were assessed. (**G-I**). Lung tissues were fixed and stained with F4/80 IHC. The representative staining images are shown in (**G-H**). The F4/80^+^ staining results from randomly selected biological replicates are shown in (**I**). (**J**). Ki-67-positive macrophages and their changes following acetoacetate treatment were evaluated. (**K-L**). The gene mRNA expression levels of ARG-1, PDGF-A, PDGF-C, MMP2, and MMP9 in lung tissues of LLC or 4T1 tumor models were measured by qPCR. Values are presented as mean ± SD, with * *p* < 0.05, ** *p* < 0.01, *** *p* < 0.001, and n.s. indicating no significance, determined by one-way ANOVA (**D, E, J**), Welch’s one-way ANOVA (**E**), Kruskal–Wallis test (**F**), and Student’s *t*-test (**I, K-L**)

To elucidate the immunomodulatory effects of acetoacetate in the metastatic lung tumor microenvironment, we conducted flow cytometric analyses to profile key immune cell populations, including T cells (CD4^+^ and CD8^+^), natural killer (NK) cells, tumor-associated macrophages (TAMs), myeloid-derived suppressor cells (MDSCs), and dendritic cells (DCs) (Fig. 2A–C). Acetoacetate treatment exerted minimal impact on T and NK cell populations (Fig. 2D). However, it significantly reduced myeloid cell infiltration, especially TAMs (Fig. 2E). Since TAMs mainly display an immunosuppressive M2-like phenotype, we further examined acetoacetate’s effect on M1 (CD86^+^) and M2 (CD206^+^) macrophage subsets (Fig. 2C). Treatment with acetoacetate notably decreased the proportion of M2-like TAMs (Fig. 2F). Immunohistochemical staining confirmed that acetoacetate significantly reduced the number of F4/80^+^ macrophages in the lung tumor microenvironment in the tail vein–induced metastatic lung cancer model, consistent with similar reductions observed in the orthotopic breast cancer metastatic lung cancer model (Fig. 2G–I).

Since the enhanced proliferative capacity of TAMs correlates positively with TAM density and a poor clinical prognosis [25], we next examined whether acetoacetate influences TAM proliferation. Flow cytometric analysis of the proliferation marker Ki-67 showed that acetoacetate treatment significantly reduced the proportion of Ki-67^+^ TAMs (Fig. 2C, J), indicating its inhibitory effect on TAM infiltration. Additionally, acetoacetate markedly decreased the expression of TAM-associated pro-metastatic factors—including arginase-1 (ARG-1), platelet-derived growth factor (PDGF), and matrix metalloproteinases MMP2 and MMP9 [26, 27]—in both tail vein and orthotopic breast cancer–induced metastatic lung cancer models (Fig. 2K–L).

Collectively, these findings show that acetoacetate effectively inhibits TAM infiltration, proliferation, and pro-metastatic activation within the lung tumor microenvironment, thereby shifting the immune landscape toward an anti-metastatic state.

### Acetoacetate suppresses metastatic lung cancer by functioning as an endogenous agonist of GPR43

**Fig. 3:**
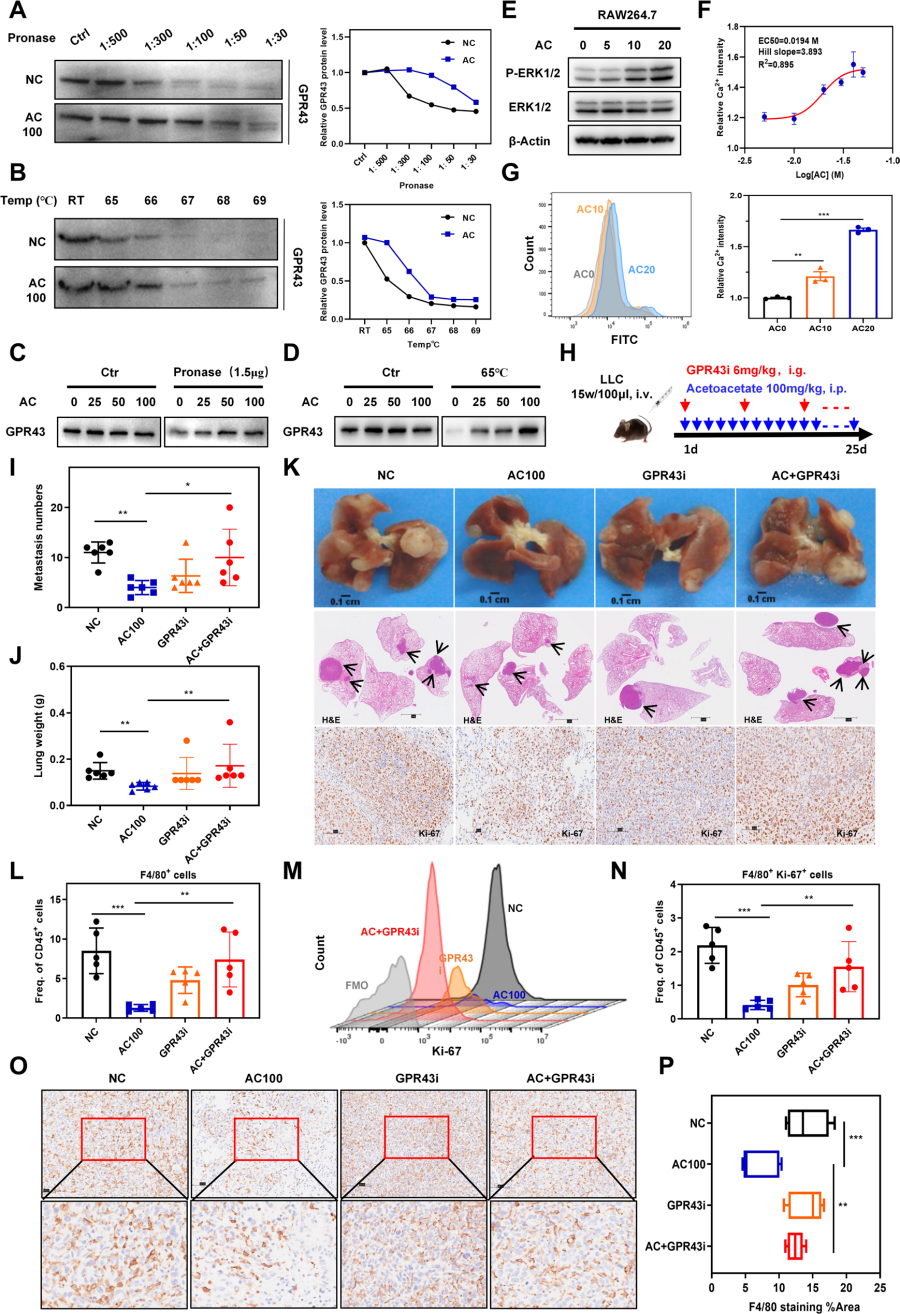
Acetoacetate suppresses metastatic lung cancer by functioning as an endogenous agonist of GPR43. (**A-B**). Immunoblot analysis of DARTS and CETSA samples, and quantitative results using ImageJ. (**C-D**). RAW264.7 cell lysates were incubated with acetoacetate at increasing concentrations (AC, 0, 25, 50, 100 mM), then subjected to DARTS and CETSA assays and analyzed by immunoblotting. (**E**). RAW264.7 cells were treated with acetoacetate (AC, 5, 10, 20 mM) for 24 hours, then harvested and analyzed by Western blot. (**F-G**). RAW264.7 cells were treated with graded concentrations of acetoacetate (AC, 0, 5, 10, 20, 30, 40, 50 mM) for 24 hours. Cells were then harvested, stained with Fluo-4, and analyzed by flow cytometry for fluorescence intensity. Statistical analysis followed. The EC_50_ is shown in (**F**). (**H**). C57BL/6 mice were intravenously injected with 1.5 × 10^5 LLC lung cancer cells via the tail vein. On the second day after modeling, they were randomly divided into four groups (*n* = 6 per group): negative control (NC), Acetoacetate (AC 100 mg/kg), GPR43 inhibitor (GPR43i), and acetoacetate combined with GPR43 inhibitor (AC+GPR43i). Acetoacetate was administered daily by i.p. injection, and the GPR43 inhibitor (GLP0974) was given by oral gavage (i.g.) every 5 days. (**I-J**). Statistical analysis of the number of metastatic lung tumors and lung weight in mice. (**K**). Lung tissues were collected after fixation and H&E staining, and lung tumors were stained for Ki-67 IHC. Representative staining images are shown. (**L**). Statistical analysis of F4/80^+^ cells across the four groups. (**M-N**). The count of Ki-67-positive macrophages (F4/80^+^ Ki-67^+^) and their statistical changes among the four groups. (**O-P**). Lung tissues were fixed and stained with F4/80 IHC. Representative staining images are shown in (**O**). F4/80^+^ staining results from randomly selected biological replicates are shown in (**P**). Values are presented as mean ± SD or mean ± SEM, with * *p* < 0.05, ** *p* < 0.01, *** *p* < 0.001, and n.s. indicating no significance, determined by one-way ANOVA and the Kruskal–Wallis test (**J**)

Previous studies have shown that GPR43 can be activated by several short-chain fatty acids, including acetate, butyrate, and propionate, mainly within the intestinal environment [28]. Based on this evidence, we hypothesized that acetoacetate, which is structurally related to short-chain fatty acids, might act as an endogenous agonist of GPR43. To test this hypothesis, we analyzed the cryo–electron microscopy (cryo-EM) structure of the acetoacetate–GPR43 complex, using the butyrate-bound GPR43 structure [29] as a reference. Molecular docking revealed that acetoacetate forms hydrogen bonds with key residues—Arg180 (3.3 Å), His242 (1.6 Å), and Arg255 (2.3 Å)—within the GPR43 binding pocket through its carboxylic group, similar to the binding mode of butyrate (Fig. S2A-B). Next, we used Drug Affinity–Responsive Target Stability (DARTS) and Cellular Thermal Shift Assay (CETSA) analyses to examine the direct interaction between acetoacetate and GPR43. In the DARTS assay, pronase treatment caused GPR43 degradation, but acetoacetate significantly increased GPR43 stability in a dose-dependent manner (Fig. 3A, C). Similarly, CETSA results showed that acetoacetate raised the thermal stability of GPR43 and, in a dose-dependent way, further confirmed their direct binding interaction (Fig. 3B, D). Additionally, consistent with previous responses following GPR43 activation, which activates the ERK cascade and elevates intracellular Ca^2 +^, respectively [28, 30]. We found that acetoacetate promoted ERK1/2 phosphorylation and increased Ca^2 +^ levels in a concentration-dependent manner, with a median effective concentration (EC_50_) of 19.4 mM for acetoacetate-induced Ca^2 +^ mobilization (Fig. 3E–G). These findings collectively provide structural and biochemical evidence that acetoacetate directly binds to GPR43 and may serve as an endogenous metabolic ligand.

Next, we examined whether acetoacetate suppressed metastatic lung cancer by acting as an endogenous agonist of GPR43. In vivo experiments in which GPR43 activation was pharmacologically blocked were conducted to evaluate the effect of GPR43 inhibition on acetoacetate’s anti-metastatic action (Fig. 3H). Quantitative analysis and H&E staining showed that acetoacetate significantly reduced the metastatic lung tumor burden, whereas GPR43 inhibition reversed this effect, resulting in extensive metastatic colonization (Fig. 3I–J, K). Consistently, Ki-67 immunostaining revealed that blocking GPR43 reduced the tumor-suppressive activity of acetoacetate (Fig. 3K, S2C). Flow cytometry further demonstrated that acetoacetate significantly reduced infiltration of F4/80^+^ macrophages and Ki-67^+^ proliferating TAMs in the lung tumor microenvironment, whereas co-administration of a GPR43 inhibitor partially restored these populations (Fig. 3L–N). Additionally, immunohistochemical staining for F4/80 supported these findings by confirming the reappearance of TAMs with the GPR43 inhibitor treatment (Fig. 3O–P). Collectively, these results suggest that GPR43 activation is crucial for both the anti-metastatic and TAMs-suppressive effects of acetoacetate, making GPR43 a key mediator of its therapeutic action.

### A GPR43-dependent metabolic signaling axis connects acetoacetate to the repression of IL-6 and CSF1

**Fig. 4:**
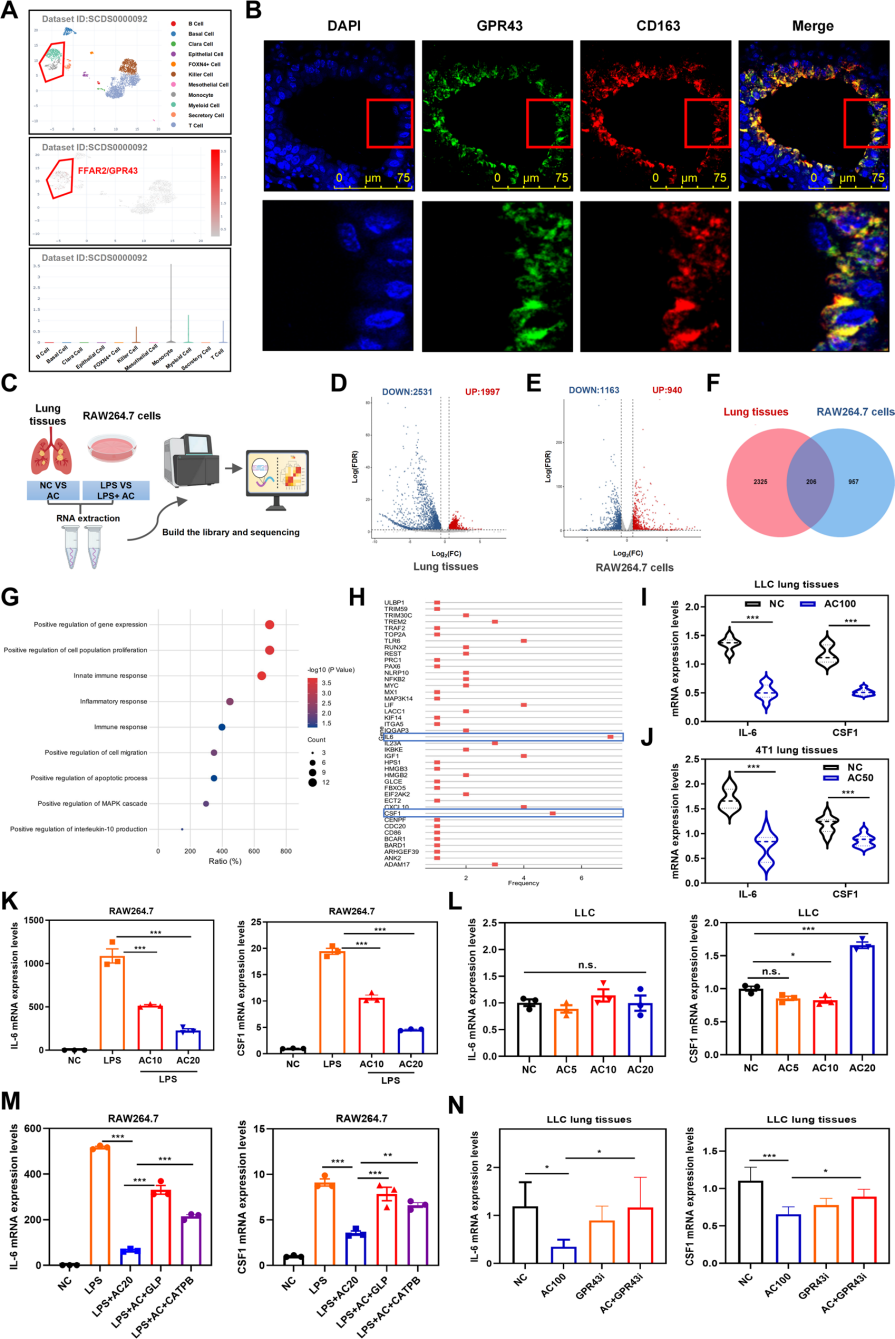
A GPR43-dependent metabolic signaling axis connects acetoacetate to the repression of IL-6 and CSF1. (A). The results of GPR43 expression in immune cells from the Cell-omics Data Coordinate Platform. (**B**). Monocytes were identified by CD163 staining, and the colocalization of GPR43 with monocytes was visualized by confocal fluorescence microscopy (DAPI, blue; GPR43, green; CD163, red; merge, orange). (**C**). A schematic drawing of the RNA-seq analysis of acetoacetate-administered LLC metastatic lung tissues and RAW264.7 cells (acetoacetate 20 mM treated cells for 24 hours, with LPS 5 µg/mL added 6 hours before the cells were harvested). (**D-E**). Volcano plot analysis was performed on lung tissues and RAW264.7 cells. (**F**). A Venn diagram showing co-down-regulated genes in mouse lung tissues and RAW264.7 cells after acetoacetate treatment. (**G-H**). Gene Ontology Biological Process (GO-BP) analysis was conducted using the DAVID website, focusing on the frequency of representative genes associated with the enriched pathways. (**I-J**). The mRNA levels of IL-6 and CSF1 were quantified by qPCR in lung tissues from the LLC and 4T1 metastatic lung tumor models. (**K**). RAW264.7 cells were treated with acetoacetate (AC, 10 mM and 20 mM) for 24 hours, with LPS added 6 hours before harvesting. The mRNA levels of IL-6 and CSF1 were measured by qPCR. (**L**). LLC cells were treated with acetoacetate (AC, 5 mM, 10 mM, 20 mM) for 24 hours, then harvested and analyzed through qPCR. (**M**). RAW264.7 cells were treated with acetoacetate (AC, 20 mM) and combined with GPR43 inhibitors (AC+GLP0974 80 µM or AC+CATPB 40 µM) for 24 hours, then harvested and assessed by qPCR. (**N**). The mRNA levels of IL-6 and CSF1 were quantified by qPCR in lung tissues from the LLC model across four groups. Values are presented as mean ± SD or mean ± SEM, with * *p* < 0.05, ** *p* < 0.01, *** *p* < 0.001, and n.s. indicating no significance, determined by one-way ANOVA and Student’s *t*-test (**I, J**)

To clarify the molecular mechanism underlying acetoacetate’s suppression of TAM infiltration and metastatic lung cancer, we examined GPR43 expression across immune cell populations using the Cell-omics Data Coordination Platform (CDCP). The analysis showed that GPR43 was primarily expressed in myeloid lineages, with the highest levels in monocytes (Fig. 4A). Consistently, immunofluorescence staining revealed clear colocalization of GPR43 with monocyte markers in lung tissues from metastatic mice, confirming its myeloid-specific expression pattern in the metastatic microenvironment (Fig. 4B).

Based on these findings, we hypothesized that acetoacetate primarily regulates gene expression in monocytes, thereby affecting TAM infiltration and lung cancer metastasis. To systematically identify its downstream molecular targets, we conducted RNA sequencing on both metastatic lung tissues and monocytes (RAW264.7 cells) following acetoacetate treatment (Fig. 4C). In metastatic lung tissues, acetoacetate treatment led to the downregulation of 2,531 genes and upregulation of 1,997 genes, while in RAW264.7 cells, only modest transcriptional changes occurred compared to lung tissues, with 1,163 genes downregulated and 940 upregulated (Fig. 4D–E). Integrated transcriptomic analysis identified 206 genes that were consistently downregulated in both metastatic lung tissues and RAW264.7 cells (Fig. 4F). Pathway enrichment analysis highlighted IL-6 and CSF1 as key regulatory nodes (Fig. 4G–H, S3A).

Next, we examined the effects of acetoacetate on IL-6 and CSF1 expression in both in vivo and in vitro settings. Quantitative PCR analysis showed significant reductions in IL-6 and CSF1 transcript levels in acetoacetate-treated mice across both the tail vein–induced metastatic lung cancer model and the orthotopic breast cancer metastatic lung cancer model (Fig. 4I–J). Consistently, acetoacetate markedly suppressed IL-6 and CSF1 expression in RAW264.7 or THP1 monocytes, as well as in primary peritoneal macrophages (PM), reinforcing its direct regulatory effect on pro-metastatic cytokines (Fig. 4K, S3B-C). In contrast, acetoacetate treatment did not change IL-6 or CSF1 expression in tumor cells (LLC and CMT167), indicating that its inhibitory effects are specific to monocyte-derived cytokine production rather than tumor cell (Fig. 4L, S3D). Collectively, these findings demonstrate that acetoacetate selectively downregulates IL-6 and CSF1 expression in monocytes.

To determine whether acetoacetate-mediated suppression of IL-6 and CSF1 depends on GPR43 signaling, RAW264.7 cells were treated with acetoacetate and the selective GPR43 inhibitors GLPG0974 [31] or CATPB [32]. Pharmacological inhibition of GPR43 significantly restored IL-6 and CSF1 expression compared to acetoacetate treatment alone (Fig. 4M). Similarly, in metastatic lung tissues, blocking GPR43 partially prevented the acetoacetate-induced decrease in IL-6 and CSF1 levels (Fig. 4N). Additionally, we knocked down GPR43 with siRNA and found that this attenuated acetoacetate’s inhibitory effect on IL-6 and CSF1, consistent with findings using GPR43 inhibitors (Fig. S3E-G). These results show that GPR43, predominantly expressed in monocytes, is crucial for mediating the acetoacetate-driven repression of IL-6 and CSF1 secretion, thereby linking metabolic signaling to the regulation of pro-metastatic cytokine expression.

### Acetoacetate suppresses TAM infiltration and metastatic lung cancer by downregulating IL-6 and CSF1 levels

**Fig. 5:**
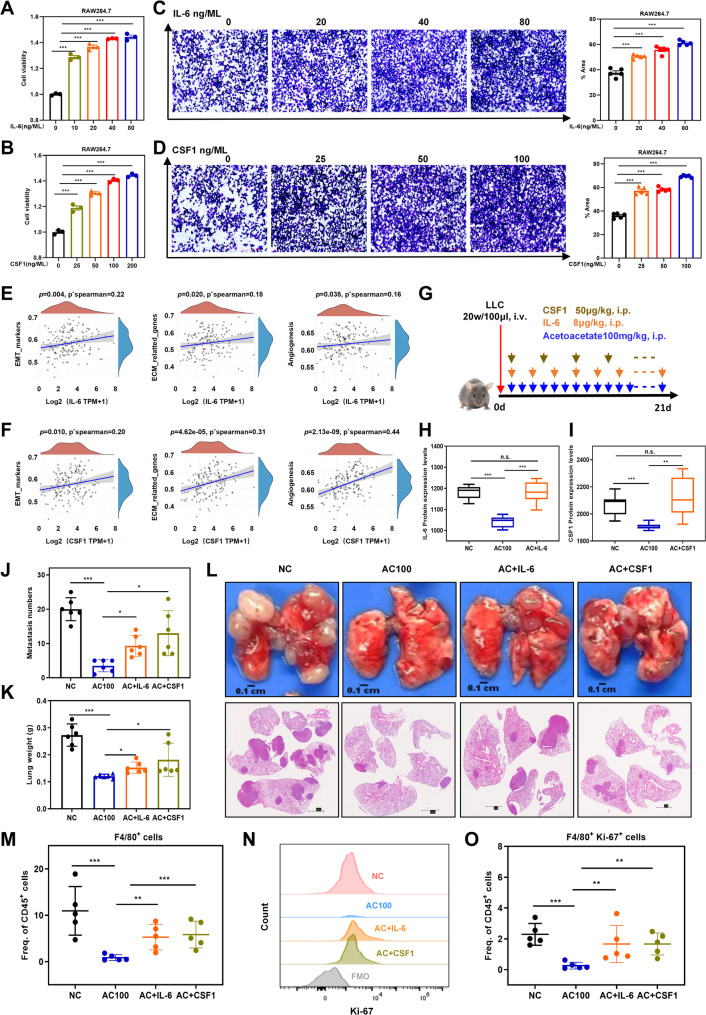
Acetoacetate suppresses TAM infiltration and metastatic lung cancer by downregulating IL-6 and CSF1 levels. (**A-B**). Raw264.7 cells were treated with the indicated concentrations of IL-6 and CSF1 for 24 hours, and cell viability was assessed using ATP Lite. (**C-D**). A Raw264.7 cell migration assay was performed at different concentrations of IL-6 and CSF1 to evaluate chemotactic efficacy. Representative images are shown on the left. Statistical analysis of randomly selected biological replicates is shown on the right. (**E-F**). The correlations of IL-6 and CSF1 with EMT, ECM, and angiogenesis were analyzed in pTNM stage IIB lung cancer patients using data from Home for Researchers (https://www.home-for-researchers.org). (**G**). C57BL/6 mice were intravenously injected with 2 × 10^5 LLC lung cancer cells via the tail vein. On the second day after injection, they were randomly divided into four groups (*n* = 6 per group): negative control (NC), Acetoacetate (AC), Acetoacetate combined with IL-6 (AC+IL-6), and Acetoacetate combined with CSF1 (AC+CSF1). Acetoacetate (100 mg/kg, daily), IL-6 (8 µg/kg, every 2 days), and CSF1 (50 µg/kg, every 3 days) were administered by intraperitoneal injection. On day 21, mice were euthanized, and lung tissues were harvested. (**H-I**). IL-6 and CSF1 protein levels in lung tissues were measured by ELISA. (**J-K**). Statistical analysis of metastatic lung tumor counts and lung weight in mice. (**L**). Lung tissues were photographed and then fixed for H&E staining. (**M**). Statistical analysis of F4/80^+^ cells across the four groups. (**N-O**). The count of Ki-67-positive macrophages (F4/80^+^ Ki-67^+^) and their changes among the four groups. Values are presented as mean ± SD or mean ± SEM, with * *p* < 0.05, ** *p* < 0.01, *** *p* < 0.001, and n.s. indicating no significance, determined by one-way ANOVA and Welch’s one-way ANOVA (**I-K**)

To investigate the functional roles of IL-6 and CSF1 in monocyte biology, we determined the proliferation and migration in acetoacetate-treated RAW264.7 cells. ATP-based cell viability assays showed that IL-6 and CSF1 significantly increased RAW264.7 cell proliferation in a dose-dependent manner (Fig. 5A–B). Likewise, transwell assays indicated that IL-6 and CSF1 greatly boosted the migratory capacity of RAW264.7 cells (Fig. 5C–D), highlighting their pro-proliferative and pro-migratory effects on macrophage-lineage cells. Consistent with these findings, clinical data analyses revealed a positive correlation between metastatic signaling pathways and IL-6 or CSF1 expression (Fig. 5E–F), implying that these cytokines serve as key downstream effectors in acetoacetate-mediated suppression of metastatic lung cancer.

To further assess whether IL-6 and CSF1 supplementation can reverse the anti-metastatic effects of acetoacetate in vivo, we performed a rescue experiment using a tail vein–induced metastatic lung cancer model (Fig. 5G). ELISA analysis confirmed that acetoacetate treatment significantly lowered IL-6 and CSF1 levels, which were restored by exogenous cytokine supplementation (Fig. 5H–I). Quantitative analysis of lung tumor burden and lung weight showed that IL-6 and CSF1 supplementation diminished the inhibitory effects of acetoacetate on metastatic lung cancer (Fig. 5J–K), aligning with histopathological evidence from H&E staining (Fig. 5L). Flow cytometry further demonstrated that reintroducing IL-6 and CSF1 restored the population and proliferative activity of F4/80^+^ macrophages, both of which had been suppressed by acetoacetate treatment (Fig. 5M–O). In summary, these findings suggest that acetoacetate reduces IL-6 and CSF1 levels within the lung tumor microenvironment, thereby decreasing TAM proliferation and migration, and hindering metastatic progression.

### Acetoacetate inhibits IL-6 and CSF1 transcription via GPR43-dependent MAT2A-SAM increase and DNA hypermethylation

**Fig. 6:**
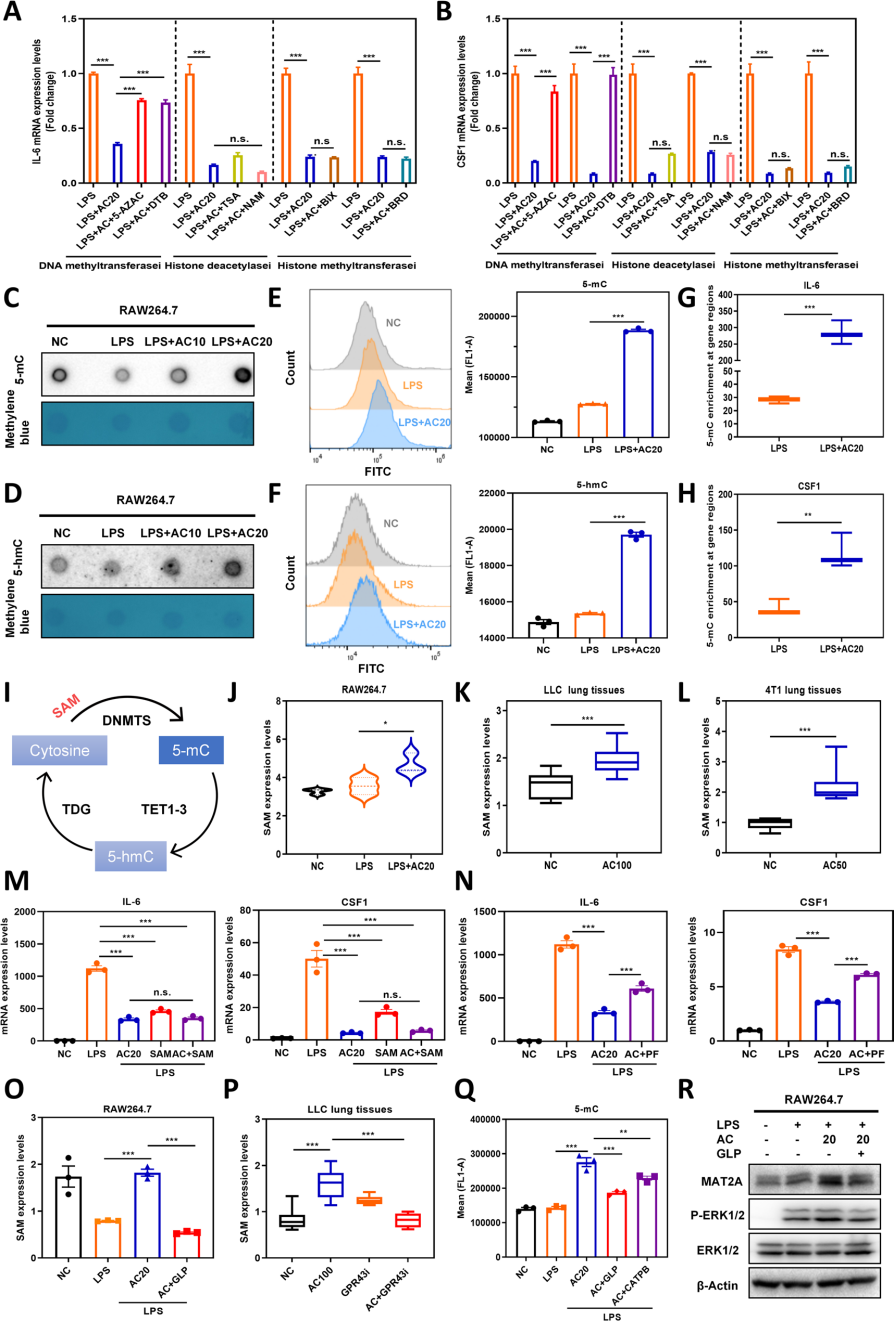
Acetoacetate inhibits IL-6 and CSF1 transcription via GPR43-dependent MAT2A-SAM increase and DNA hypermethylation. (**A-B**). RAW264.7 cells were treated with acetoacetate (AC, 20 mM) and combined with 5-AZAC (2.5 µM), DTB (1 µM), TSA (5 µM), NAM (10 mM), BIX (0.1 µM), or BRD (5 µM) for 24 hours, with LPS added 6 hours before cell harvest. (**C-D**). RAW264.7 cells were incubated with acetoacetate at specified concentrations for 24 hours, DNA was extracted, and levels of 5-mC or 5-hmC were detected by dot blot. (**E-F**). RAW264.7 cells were incubated with acetoacetate at specified concentrations for 24 hours, and 5-mC or 5-hmC levels were measured by flow cytometry. (**G-H**). The enrichment of 5-mC in the IL-6 and CSF1 gene regions was quantified using MEDIP-qPCR. (**I**). A schematic illustrating the DNA methylation process. (**J-L**). Levels of SAM expression in acetoacetate-treated RAW264.7 cells and in lung tissues from LLC or 4T1 mouse models were analyzed by ELISA. (**M-N**). RAW264.7 cells were treated with acetoacetate (AC, 20 mM), SAM (200 mM), acetoacetate and SAM together, or combined PF (1 µM) for 24 hours, with LPS added 6 hours before harvest. (**O-P**). SAM expression levels were compared across groups in RAW264.7 cells and in LLC lung tissues by ELISA. (**Q**). 5-mC levels in the indicated groups were detected using flow cytometry. (**R**). RAW264.7 cells were treated with acetoacetate (AC, 20 mM) or with a combination of AC and GLP0974 (80 µM) for 24 hours, with LPS added 6 hours before harvest. Samples were then analyzed by Western blot. Values are presented as mean ± SD or mean ± SEM, with * *p* < 0.05, ** *p* < 0.01, *** *p* < 0.001, and n.s. indicating no significance, determined by one-way ANOVA and Student’s *t*-test (**G-H, K-L**)

To elucidate the mechanism by which acetoacetate suppresses IL-6 and CSF1 transcription, we first examined key upstream transcription factors, including NF-κB [33], STAT3 [34], HIF-1α [35], and AP-1 (c-Jun) [36]. Western blotting and nucleoplasm fractionation analyses showed that acetoacetate did not change the expression or nuclear translocation of these factors (Fig. S4A-D). Given the well-established role of epigenetic modifications in regulating transcription [37], we tested whether acetoacetate influences DNA methylation or histone modifications. RAW264.7 cells were co-treated with acetoacetate and a panel of epigenetic inhibitors, including DNA methyltransferase inhibitors (5-AZAC [38] and DTB [39]), histone deacetylase inhibitors (TSA [40] and NAM [41]), and histone methyltransferase inhibitors (BIX [42] and BRD [43]). Among these, only the DNA methyltransferase inhibitors 5-AZAC and DTB effectively reversed acetoacetate’s suppression of IL-6 and CSF1 expression (Fig. 6A–B). Consistently, histone isolation followed by Western blotting showed no significant changes in histone acetylation or methylation at relevant loci (Fig. S4E), while global levels of 5-methylcytosine (5-mC) and 5-hydroxymethylcytosine (5-hmC) were significantly increased (Fig. 6C–F), indicating that acetoacetate mainly affects DNA methylation rather than histone modifications. Additionally, methylated DNA immunoprecipitation (MeDIP)-qPCR analysis confirmed enrichment of 5-mC at the IL-6 and CSF1 region, demonstrating that acetoacetate induces hypermethylation of IL-6 and CSF1 (Fig. 6G–H, S4F).

To elucidate how acetoacetate influences DNA methylation, we first examined the expression of key DNA methyltransferases (DNMT1, DNMT3A, DNMT3B) and the DNA hydroxylase TET2 after acetoacetate treatment. Western blot analyses revealed no significant changes in the levels of these enzymes in acetoacetate-treated RAW264.7 cells (Fig. S4G-H). Since DNA methylation relies on S-adenosylmethionine (SAM) as a methyl donor [44], we next evaluated whether acetoacetate affects intracellular SAM levels (Fig. 6I). ELISA measurements revealed a significant increase in SAM levels in both acetoacetate-treated RAW264.7 cells and metastatic lung tissues (Fig. 6J–L). We then tested whether SAM mediates acetoacetate’s suppression of IL-6 and CSF1. As with acetoacetate, SAM reduced IL-6 and CSF1 expression; importantly, combining SAM and acetoacetate did not yield an additive effect (Fig. 6M). Conversely, co-treatment with acetoacetate and PF9366 [45], a MAT2A inhibitor, partially restored IL-6 and CSF1 expression (Fig. 6N). Western blotting further revealed that acetoacetate significantly increased MAT2A protein levels (Fig. S4I). Collectively, these findings indicate that acetoacetate inhibits IL-6 and CSF1 by upregulating MAT2A, enhancing SAM biosynthesis, and providing methyl donors for DNA hypermethylation.

Finally, we investigated whether GPR43 is required for SAM elevation and elucidated the underlying mechanism. Pharmacological blockade of GPR43 markedly attenuated acetoacetate-induced SAM accumulation in both RAW264.7 cells and metastatic lung tissues (Fig. 6O–P). Accordingly, dot blot and flow cytometry analyses confirmed that GPR43 inhibition significantly reduced acetoacetate-induced global 5 mC accumulation (Fig. 6Q, S4J). Nevertheless, the downstream signaling cascade connecting GPR43 activation to SAM upregulation remained elusive. Given that ERK1/2 phosphorylation has been reported to positively regulate MAT2A expression [46], we hypothesized that the ERK–MAT2A axis might mediate the SAM-elevating effect of acetoacetate. Strikingly, both interventions (GPR43 or ERK inhibition) partially abrogated acetoacetate-induced ERK1/2 phosphorylation and MAT2A upregulation (Fig. 6R, S4K). Furthermore, treatment with the ERK-specific inhibitor SCH772984 [47] partially reversed the acetoacetate-mediated suppression of IL-6 and CSF1 (Fig. S4L), reinforcing the functional relevance of this axis. Collectively, these findings establish that acetoacetate elevates SAM through the GPR43–pERK–MAT2A axis, which in turn drives DNA hypermethylation and subsequent transcriptional silencing of IL-6 and CSF1.

## Discussion

Metabolic reprogramming is a well-established hallmark of cancer; however, the expression status of acetoacetate-metabolizing enzymes and their impact on endogenous ketone levels in lung cancer have not been characterized. Previous findings indicate that desuccinylated HMGCS2 preferentially generates more acetoacetate rather than β-hydroxybutyrate [48]; HMGCL has tumor-suppressive effects in osteosarcoma by blocking the PI3K/AKT/mTOR pathway [49]; and OXCT1 promotes oncogenic progression across various cancer types [50]. Here, we demonstrate that acetoacetate-synthesizing enzymes (HMGCS2 and HMGCLL1) are downregulated, whereas the catabolic enzyme OXCT1 is upregulated in lung tumors, resulting in a localized deficiency of acetoacetate within the tumor microenvironment. Notably, in vivo supplementation with acetoacetate reversed this deficit and suppressed metastatic lung cancer across different models, without observable systemic toxicity. Therefore, we propose that “ketone body imbalance” constitutes a targetable metabolic vulnerability. Correcting this pathological imbalance through exogenous acetoacetate supplementation may provide a precision metabolic therapy to restore tumor-suppressive signaling.

Ketone bodies have traditionally been regarded primarily as alternative energy substrates, with acetoacetate known to sustain ATP production in nutrition-deprived tumor cells [51, 52] and to fuel acetyl-CoA–dependent programs in CD8^+^ T cells [53]. However, the potential of acetoacetate’s metabolic signaling and regulatory roles has largely remained unexplored. Our present study broadens this paradigm by demonstrating that acetoacetate also acts as a signaling ligand that directly binds to GPR43, mediating the transcriptional suppression of monocyte-derived IL-6 and CSF1 and thereby affecting tumor-associated macrophage recruitment. This discovery expands the known functions of ketone bodies beyond energy metabolism and emphasizes acetoacetate as an active immunoregulatory molecule with previously unrecognized relevance in tumor immunity.

GPR43, also known as free fatty acid receptor 2 (FFAR2), a short-chain fatty acid (SCFA) receptor [28, 30], is well established as a regulator of intestinal immunity and metabolic homeostasis [54]. GPR43 (−/−) mice exhibit exacerbated colitis and heightened inflammatory responses [55]. However, its role within tumor microenvironments, particularly in metastatic lung cancer, remains poorly defined. To address this, we implemented a systematic experimental approach: molecular docking revealed structural compatibility between acetoacetate and GPR43, indicating shared binding sites with the canonical ligand butyrate and suggesting conserved activation mechanisms. Further biophysical validation using DARTS and CETSA confirmed a direct, dose-dependent interaction: acetoacetate enhanced GPR43’s resistance to proteolysis and thermal denaturation, thereby demonstrating target engagement. In parallel, acetoacetate promoted ERK1/2 phosphorylation and Ca^2 +^ mobilization in a dose‑dependent manner, indicative of receptor activation. Moreover, functional interrogation with receptor inhibitors and siRNA demonstrated that GPR43 is indispensable for acetoacetate-mediated suppression of IL-6/CSF1 expression in both in vivo and in vitro settings. These findings extend beyond GPR43’s known role in gut pathologies [28, 56]. Critically, we demonstrate that acetoacetate, via GPR43, disrupts cytokine-driven TAM recruitment, thereby impeding metastatic niche formation. These findings provide a theoretical basis for developing acetoacetate-inspired GPR43 agonists as a potential novel therapeutic strategy.

Tumor-associated macrophages (TAMs), located within or near solid tumors, are pivotal drivers of tumor progression and metastasis [1, 57]. Tumor cells actively recruit TAMs, often by elevating cytokines like IL-6 and CSF1, which correlate with higher TAM density and poorer patient outcomes [58]. Our research provides the first direct evidence that acetoacetate reduces IL-6 and CSF1 secretion in monocytes, thereby blocking TAMs’ proliferation and migration in TME. This finding aligns with and greatly extends the known metabolic roles of acetoacetate in macrophages. Prior studies have established that acetoacetate—rather than β-hydroxybutyrate—is a vital energy source for macrophages, protecting them from lactic acidosis-induced mitochondrial damage [59], and that mitochondrial acetoacetate metabolism in macrophages guards against liver fibrosis [60]. Furthermore, acetoacetate acts as a key molecule for B3GALNT2 in regulating MIF activity and macrophage recruitment in the liver [61]. Together, acetoacetate selectively targets macrophages and functions not only as a metabolic fuel but also as a signaling molecule, serving as a crucial metabolite that directly modulates macrophage biology.

In this study, we propose a novel axis: Acetoacetate-GPR43-DNA hypermethylation-IL-6/CSF1 silencing. Consistent with this idea, DNA methylation is known to modulate these cytokines [62, 63]. However, we provide the first evidence that acetoacetate increases S-adenosylmethionine (SAM), the universal methyl donor, and induces hypermethylation of IL-6/CSF1. Notably, SAM’s immunomodulatory role is context-dependent: it suppresses LPS-induced TNF-α via histone/DNA methylation [64, 65], yet in one-carbon metabolism, SAM upregulates IL-1β through H3K36me3 histone methylation in inflammatory macrophages [66]. Our functional studies resolve this discrepancy: SAM, alone or in combination with acetoacetate, similarly inhibited IL‑6/CSF1 expression in RAW264.7 cells; however, when acetoacetate was co‑incubated with the MAT2A inhibitor (an SAM synthetase), suppression was blocked. This bidirectional validation confirms SAM’s essential role in acetoacetate-driven cytokine silencing. We uncover a novel metabolic-epigenetic-immune axis by which acetoacetate suppresses metastatic lung cancer, providing new insights into the anti-tumor effects of ketone bodies. However, we have preliminarily hypothesized that the downstream signaling link between GPR43 activation by acetoacetate and SAM biosynthesis involves the ERK cascade: GPR43 engagement enhances ERK1/2 phosphorylation, which in turn promotes MAT2A accumulation and upregulates SAM production. Nevertheless, the detailed mechanisms and direct evidence warrant a comprehensive, in‑depth investigation in future studies.

**Fig. 7:**
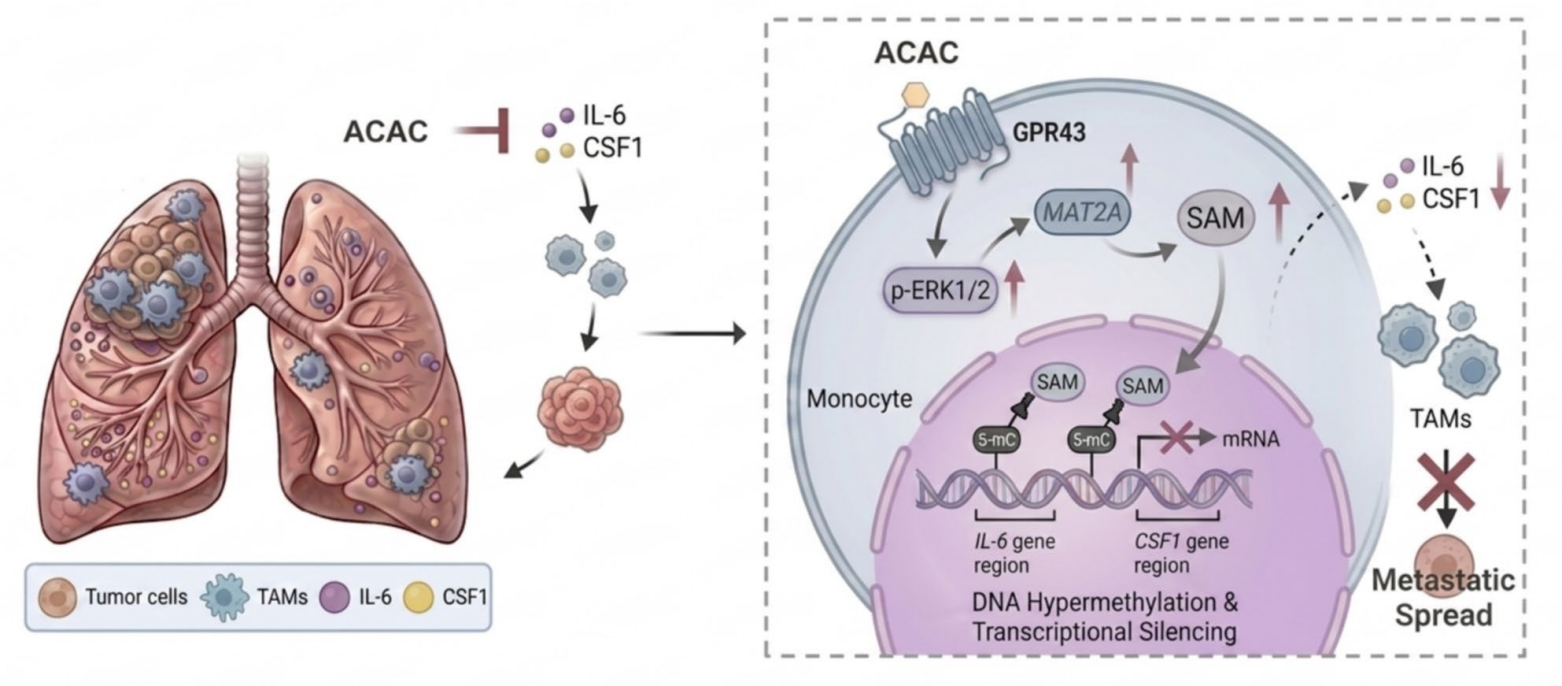
The acetoacetate working model inhibits monocyte-derived IL-6 and CSF1 via GPR43-mediated P-ERK1/2-MAT2A-SAM elevation and DNA hypermethylation, suppressing TAM infiltration and metastatic lung cancer

In summary, we demonstrate that supplementing acetoacetate inhibits metastatic lung cancer. Acetoacetate activates GPR43, which induces ERK1/2 phosphorylation, upregulates MAT2A, increases endogenous SAM levels, and leads to gene-region-specific DNA hypermethylation. This silences monocyte-derived IL-6/CSF1 expression, thereby reducing TAM infiltration (Fig. 7). These findings provide a preclinical scientific foundation for acetoacetate as a promising ketone-body therapeutic strategy to target tumor-associated macrophages (TAMs) in the metastatic lung microenvironment, offering a new potential approach to disrupt metastatic lung cancer through immunometabolic modulation.

## Electronic supplementary material

Below is the link to the electronic supplementary material.

Supplementary Material 1

Supplementary Material 2

## Data Availability

The original contributions in the study are included in the article or supplementary material. For further inquiries, please contact the corresponding authors.

## Abbreviations

TAMs: Tumor-associated macrophages
TCGA: The Cancer Genome Atlas
GTEx: Genotype-Tissue Expression
qPCR: Quantitative real-time polymerase chain reaction
DARTS: Drug affinity responsive target stability
CETSA: Cellular thermal shift assay
GPR43: G protein-coupled receptor 43
MeDIP: Methylated DNA immunoprecipitation
ELISA: Enzyme-linked immunosorbent assay
AC: Acetoacetate
IL-6: Interleukin-6
CSF1: Colony-stimulating factor 1
MAT2A: Methionine adenosyltransferase 2A
TME: Tumor microenvironment
KD: Ketogenic diet
BHB: β-hydroxybutyrate
5-mC: 5-methylcytosine
5-hmC: 5-hydroxymethylcytosine
SAM: S-adenosyl methionine
