# Supplementary material for: Acetoacetate–GPR43 axis epigenetically silences IL-6/CSF1 to restrict TAMs-driven metastatic lung cancer
Source: J Transl Med. 2026 Aug 3;24:1035. doi: 10.1186/s12967-026-08742-6 (PMC13471439; doi:10.1186/s12967-026-08742-6)

Figure 3

A

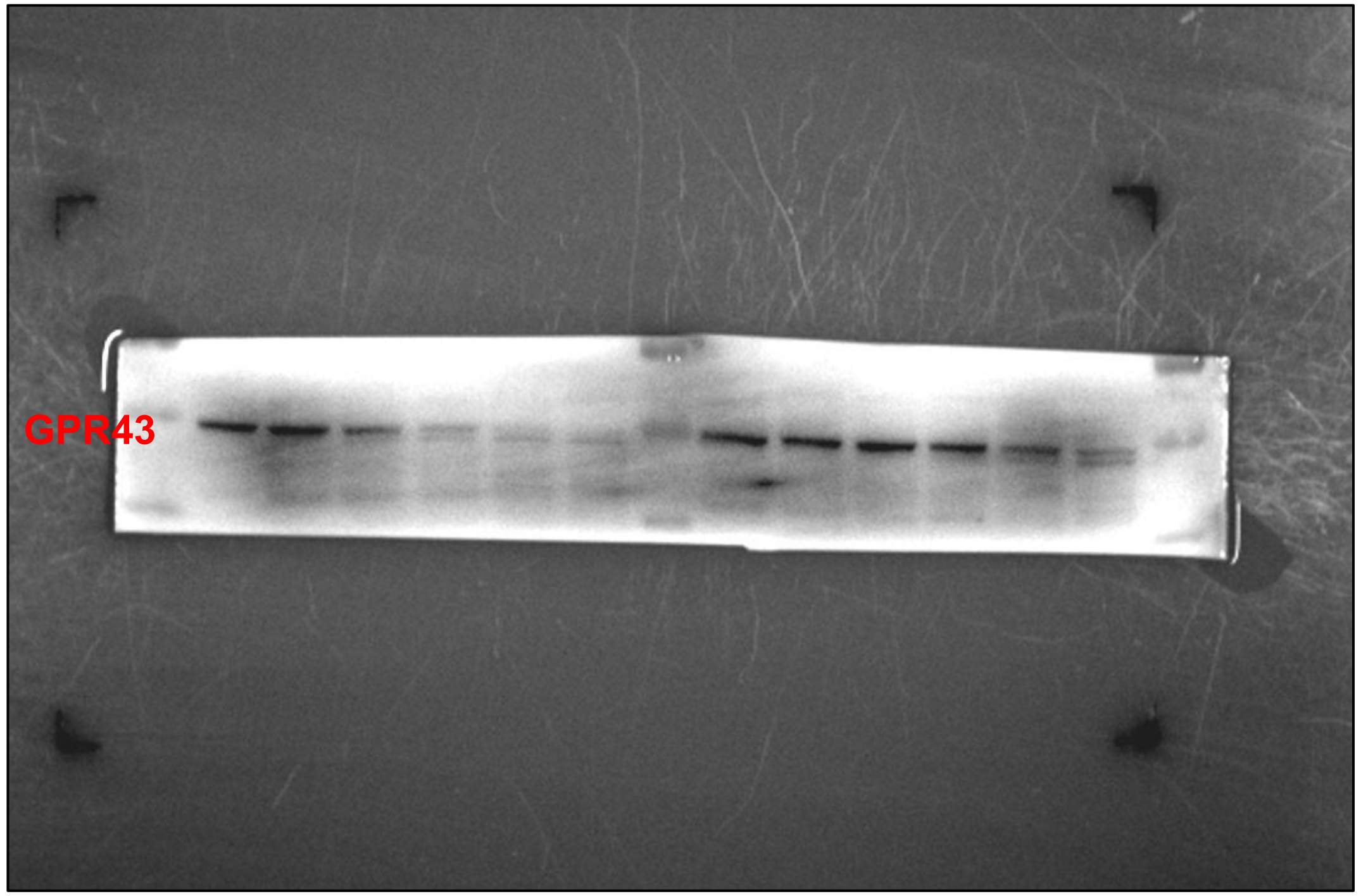

B

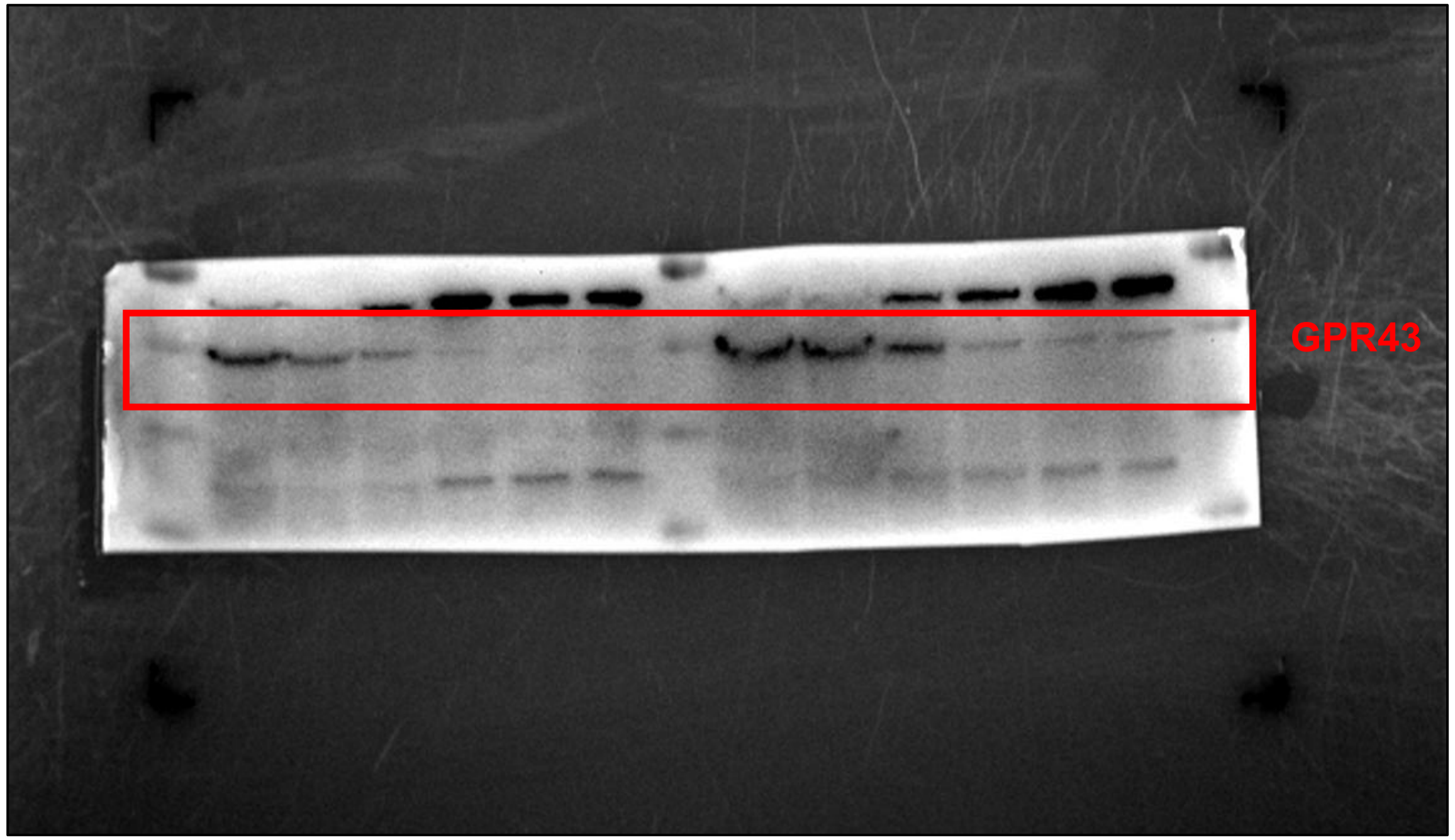

Figure 3

c

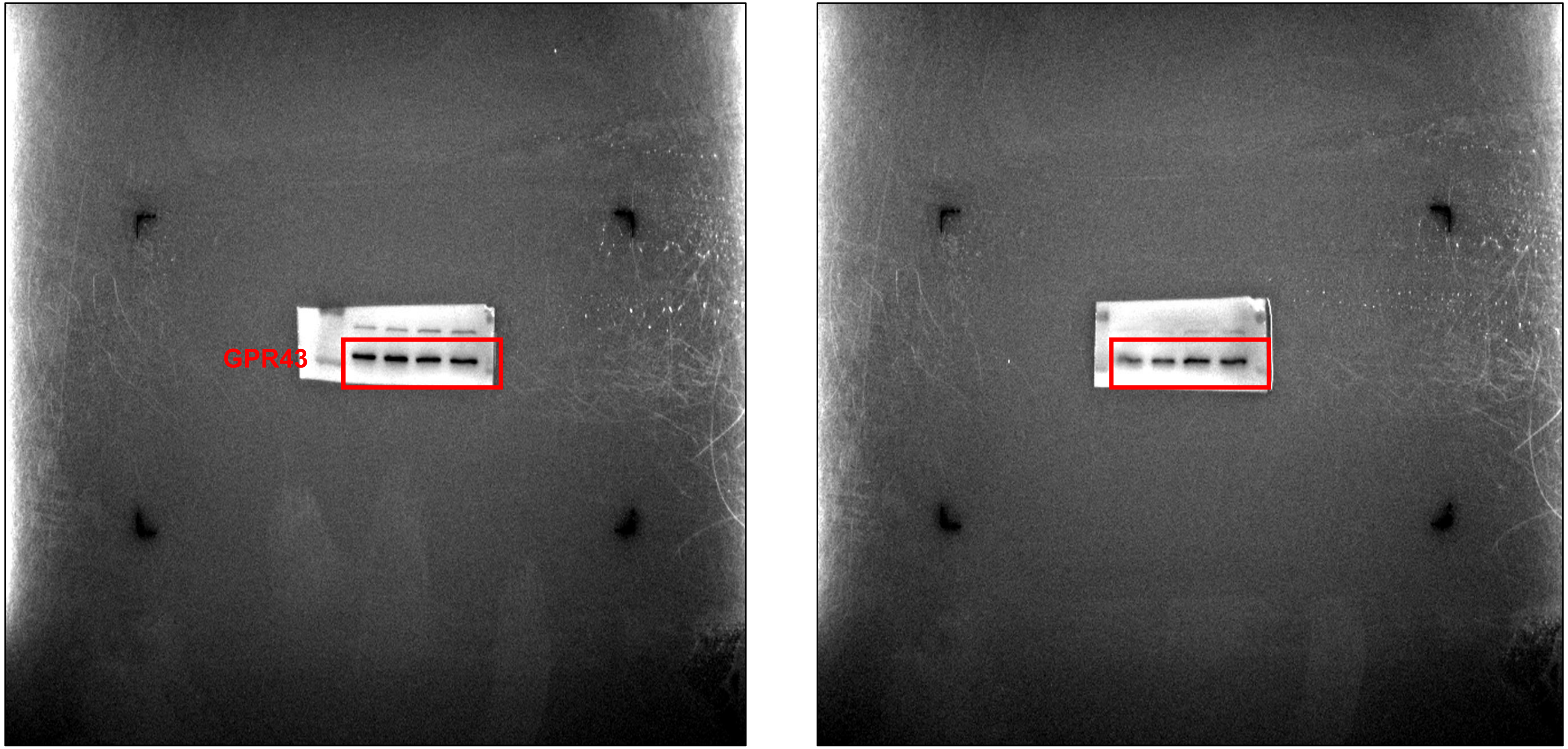

Figure 3

D

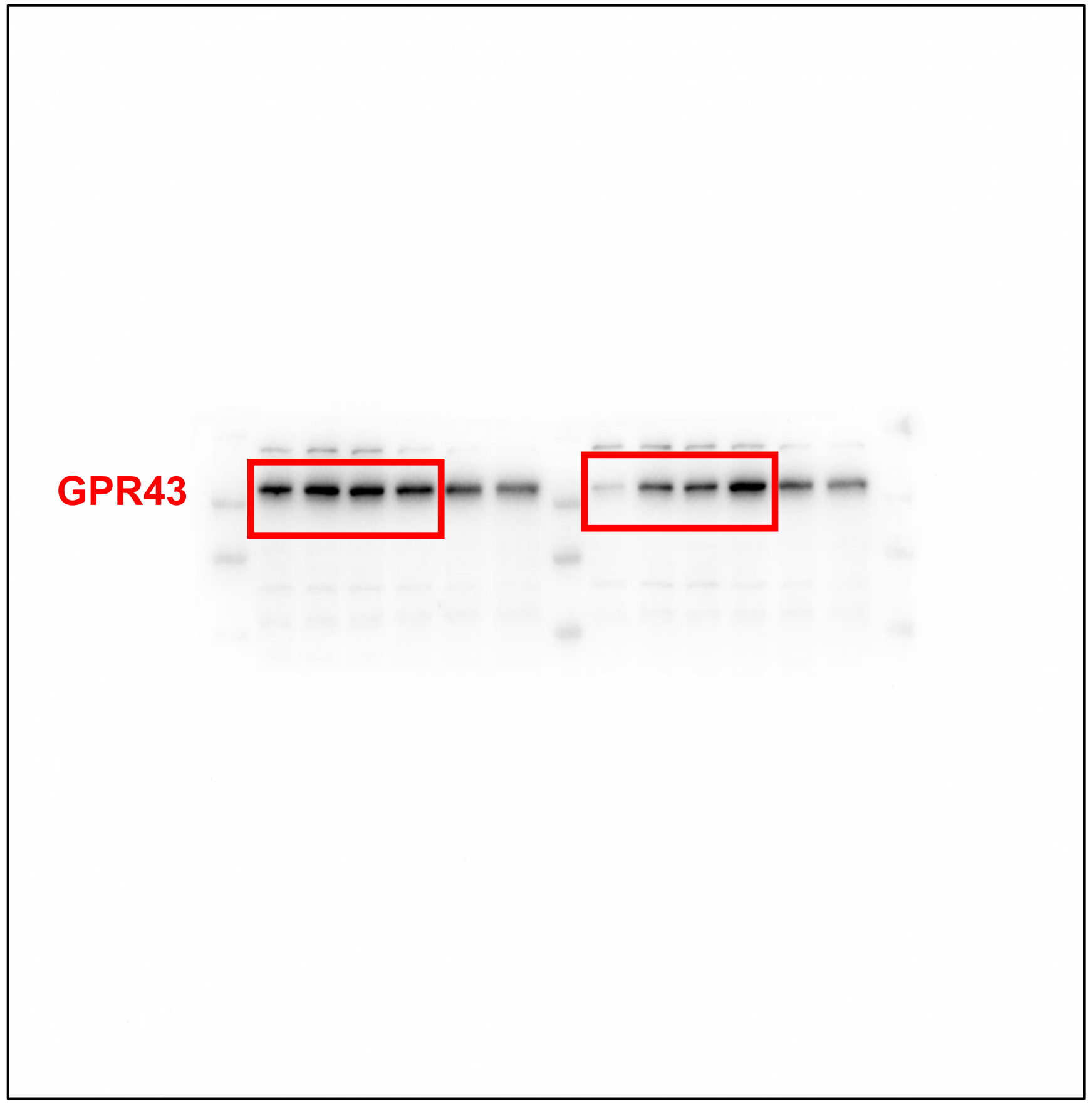

Figure 3

E

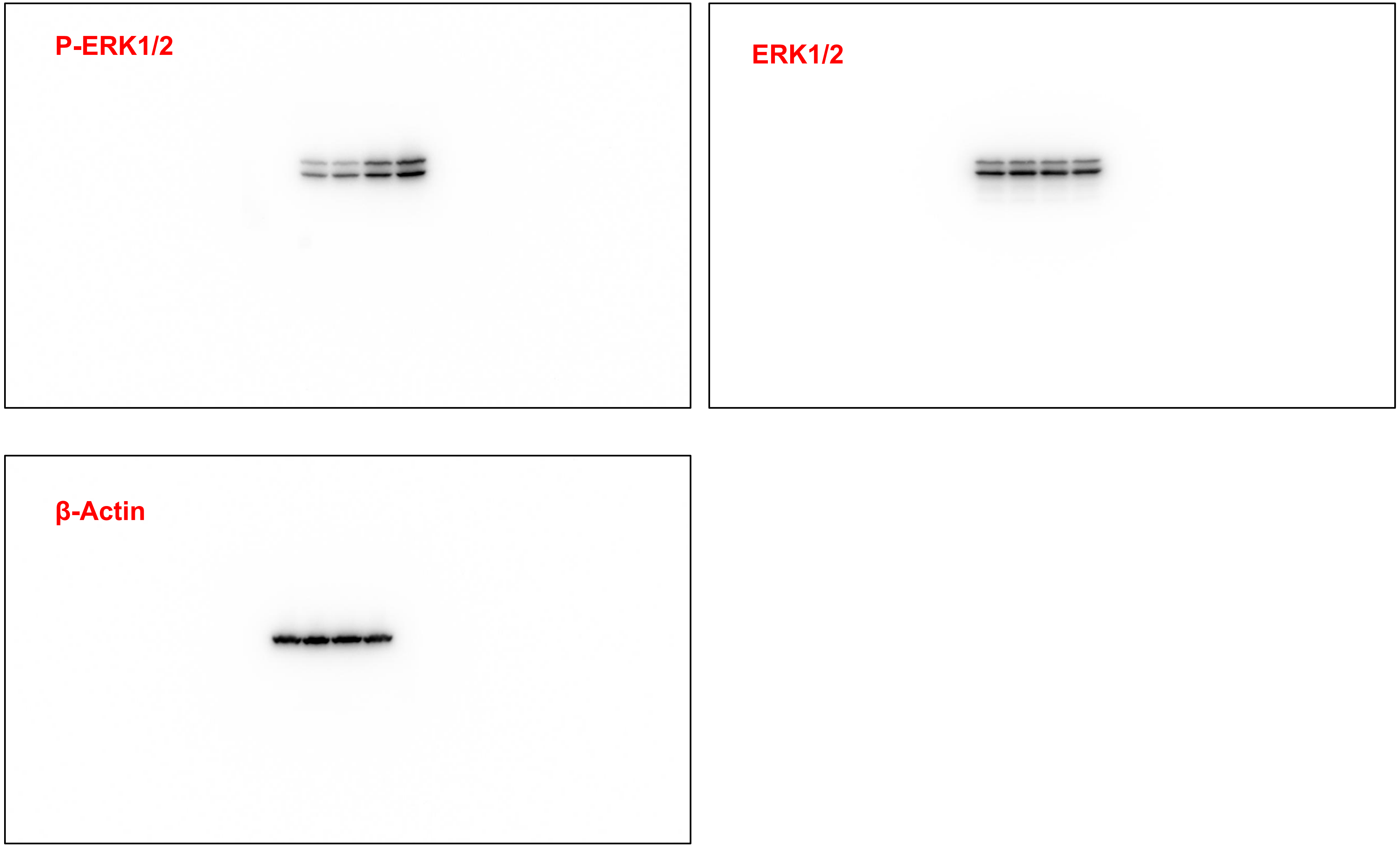

Figure 6 C

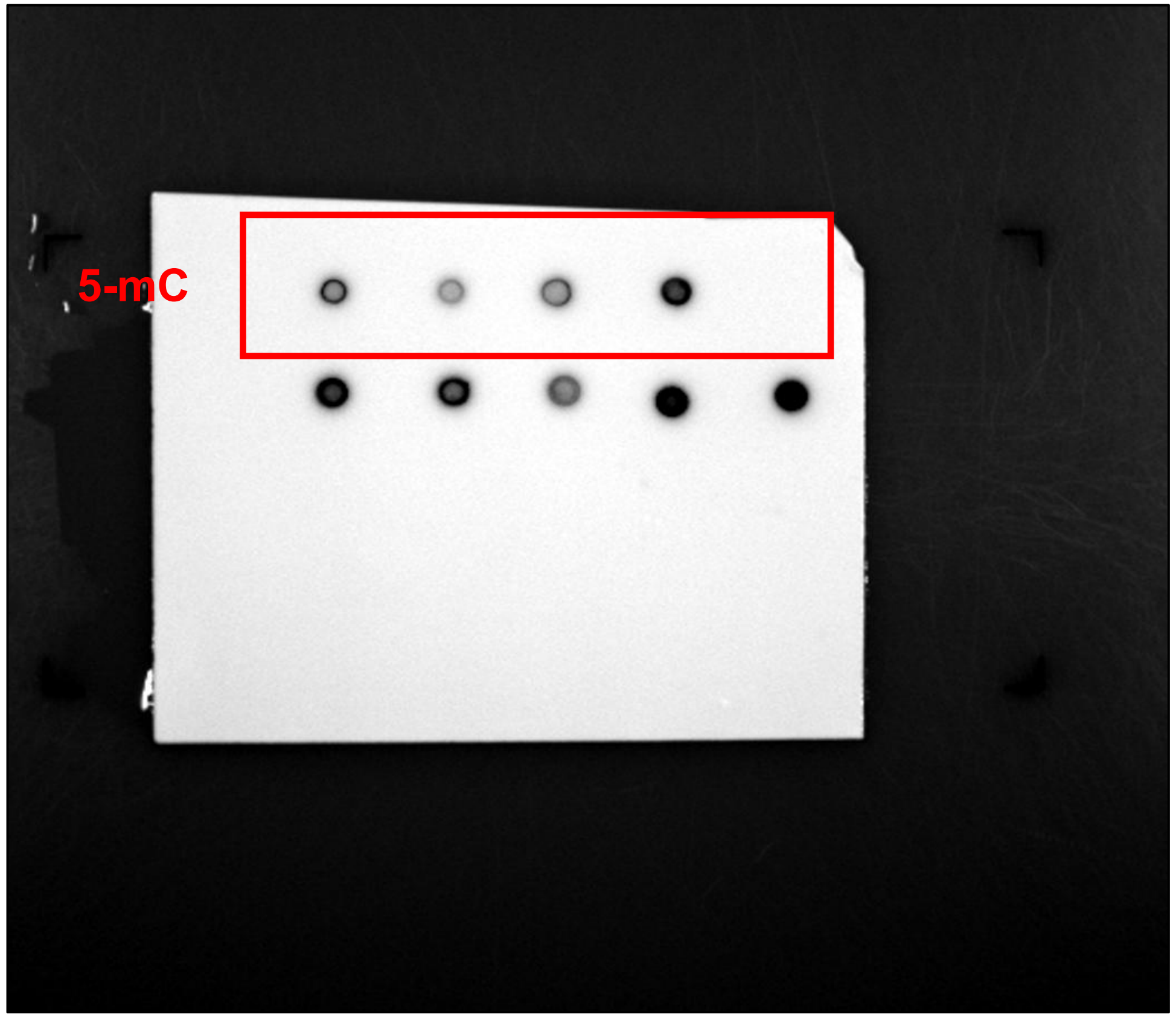

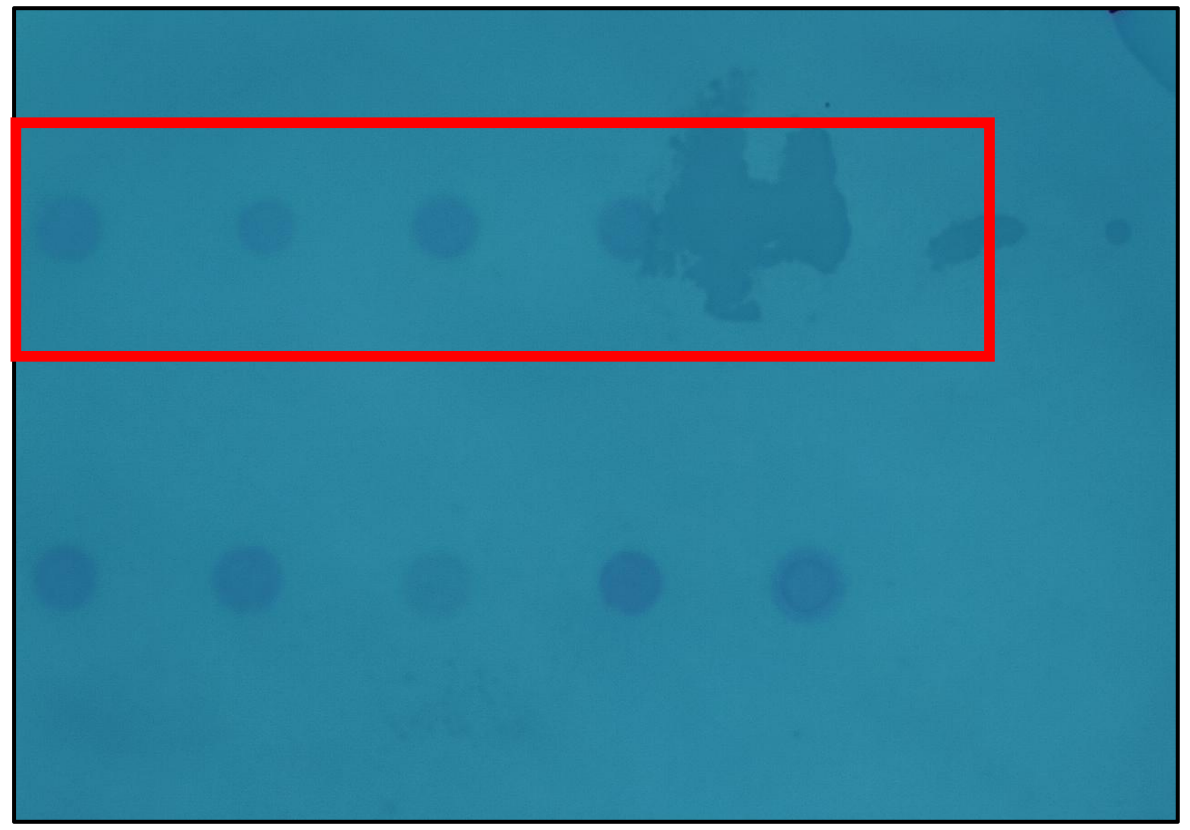

Figure 6 D

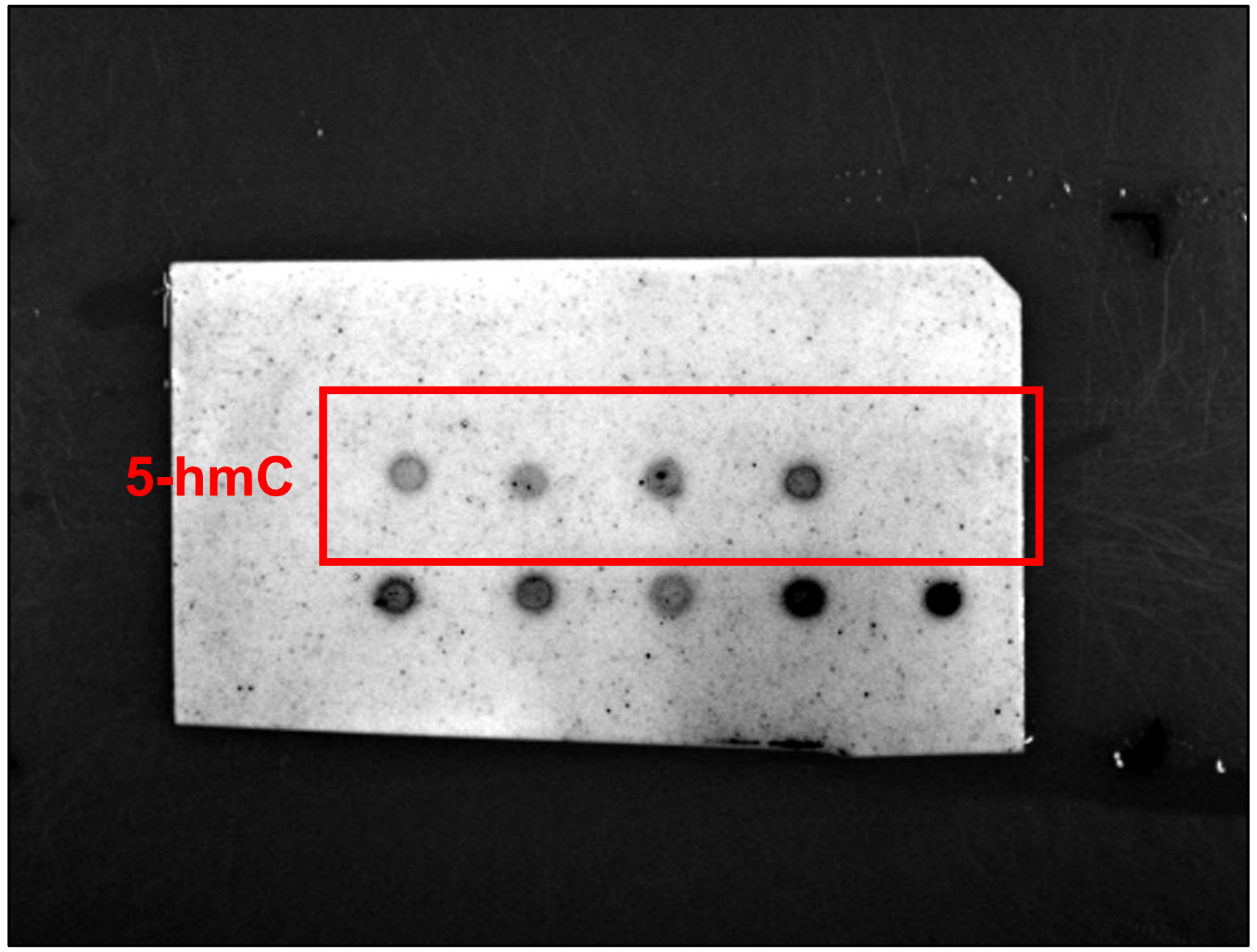

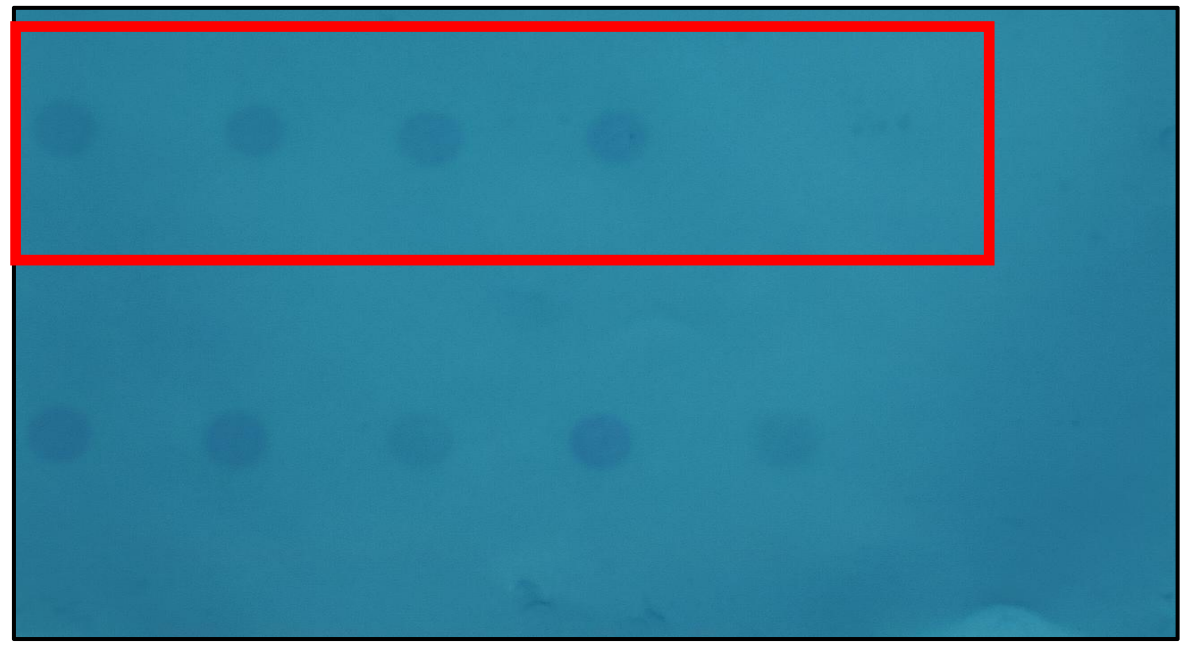

Figure 6 R

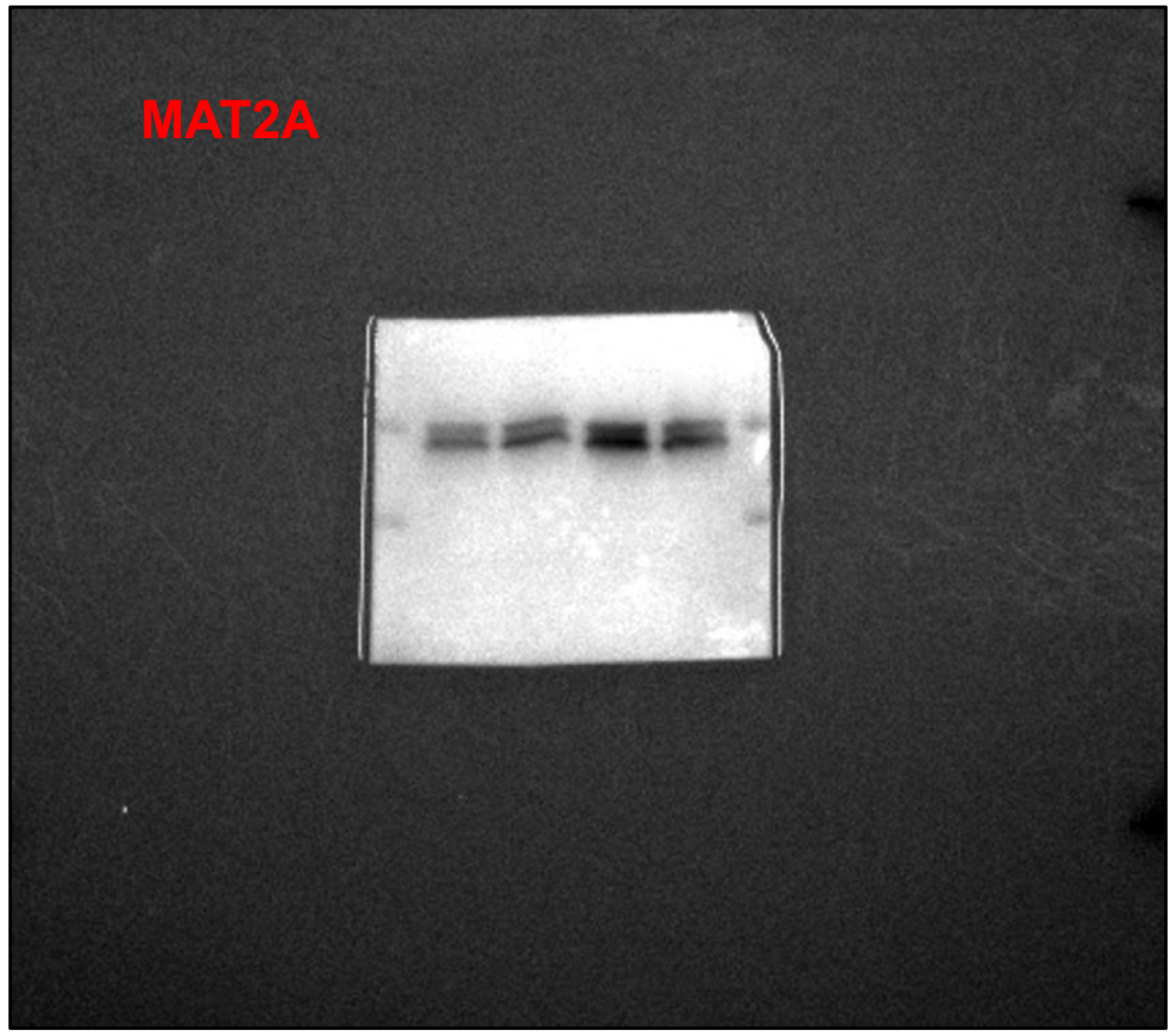

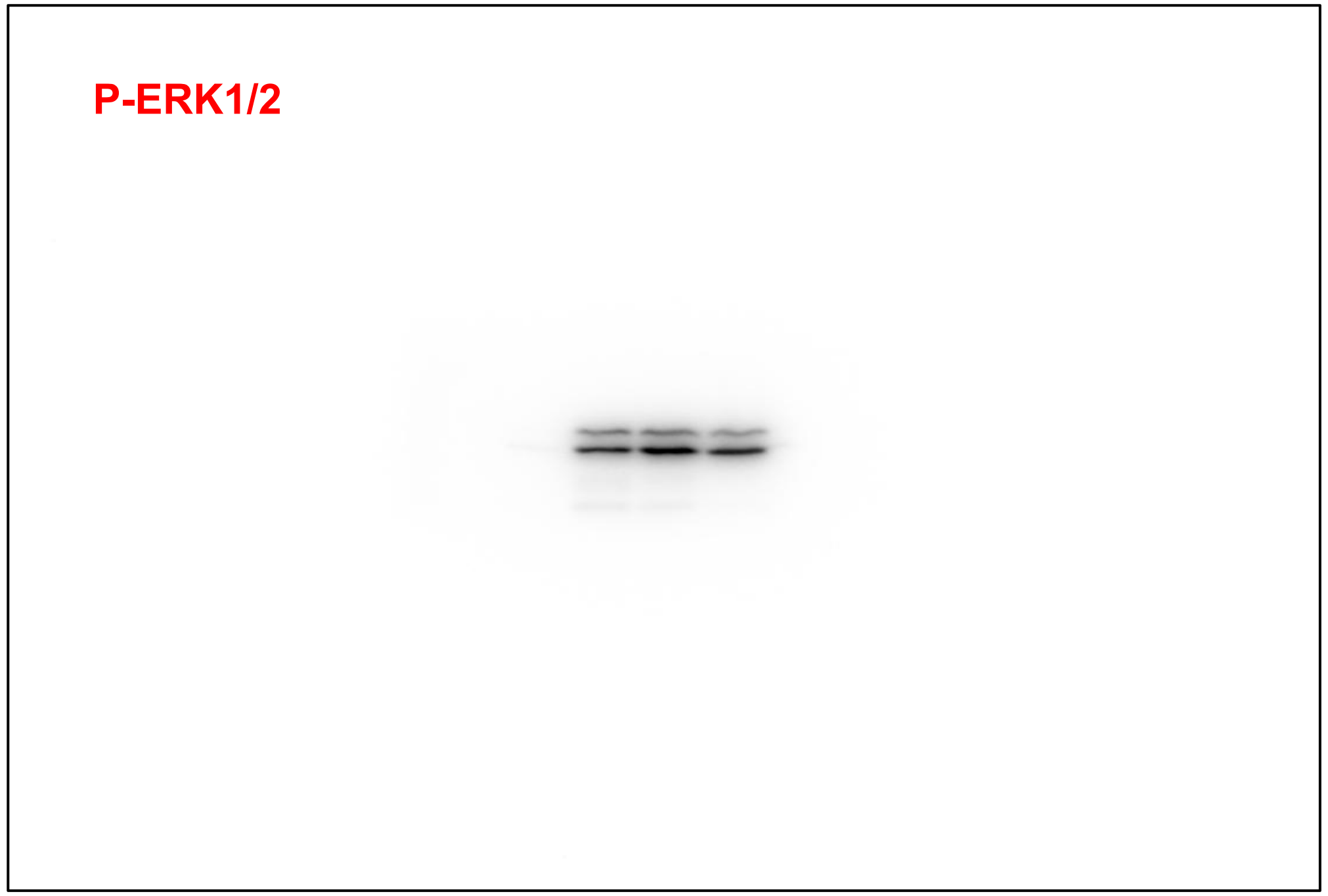

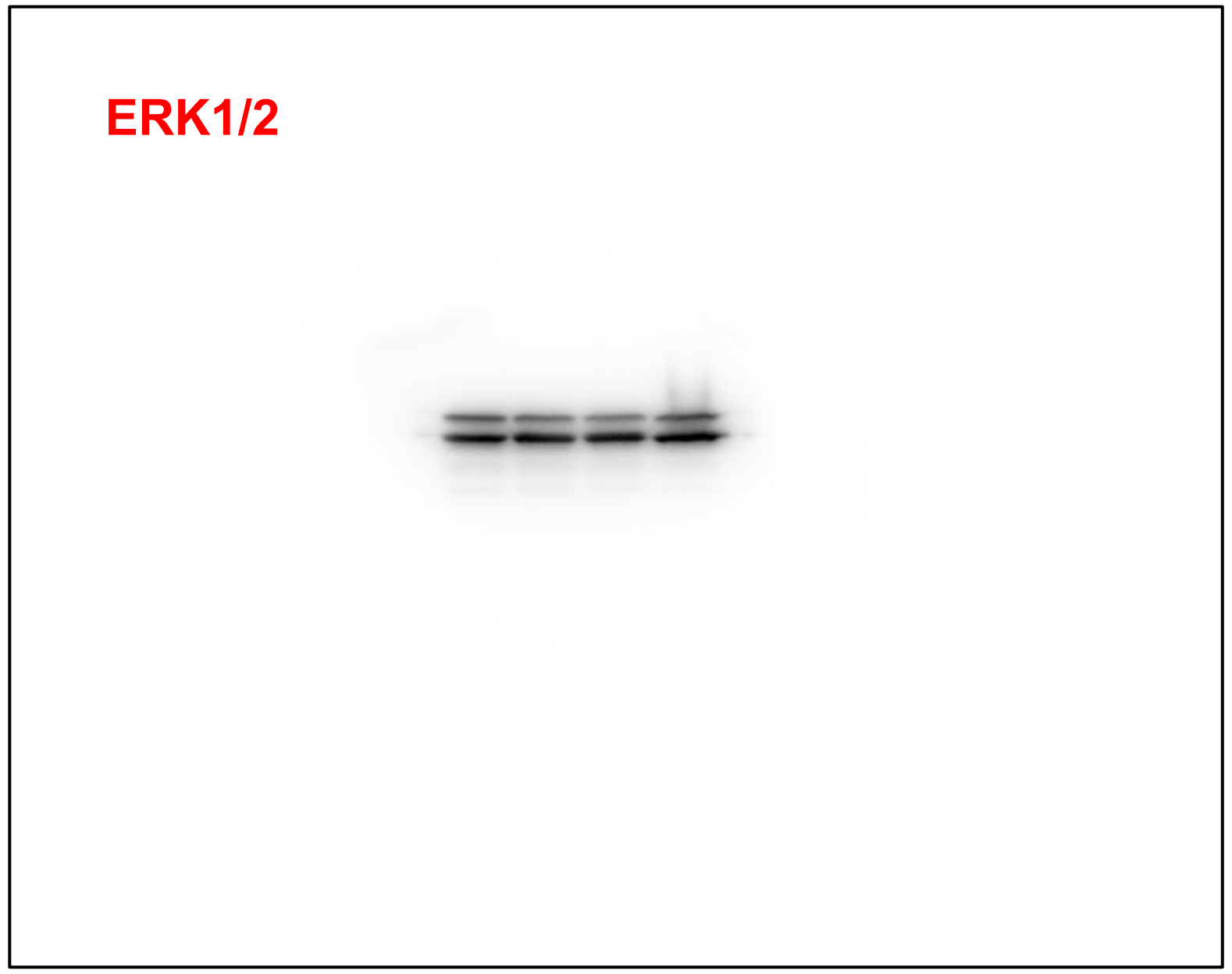

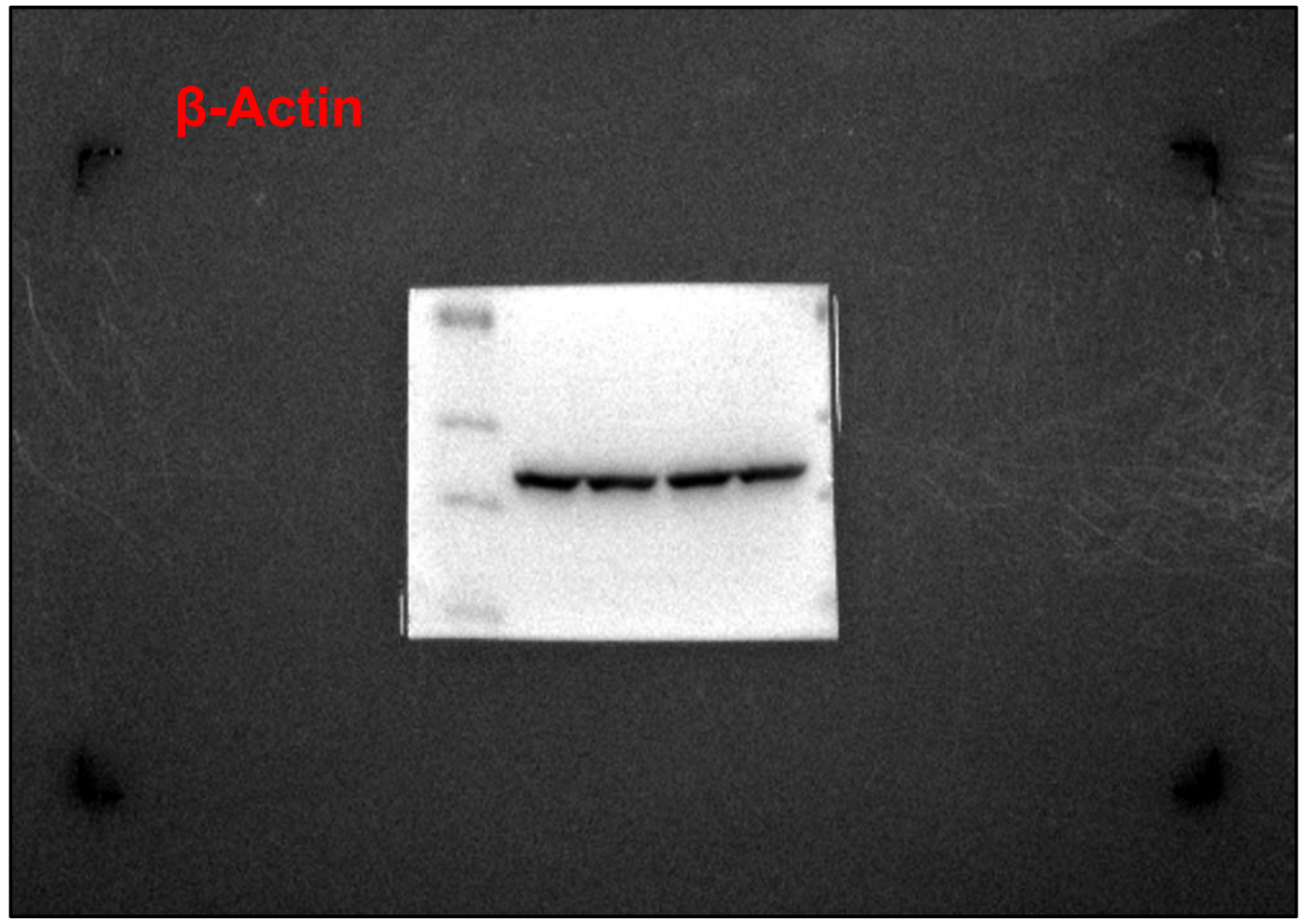

Figure S3E

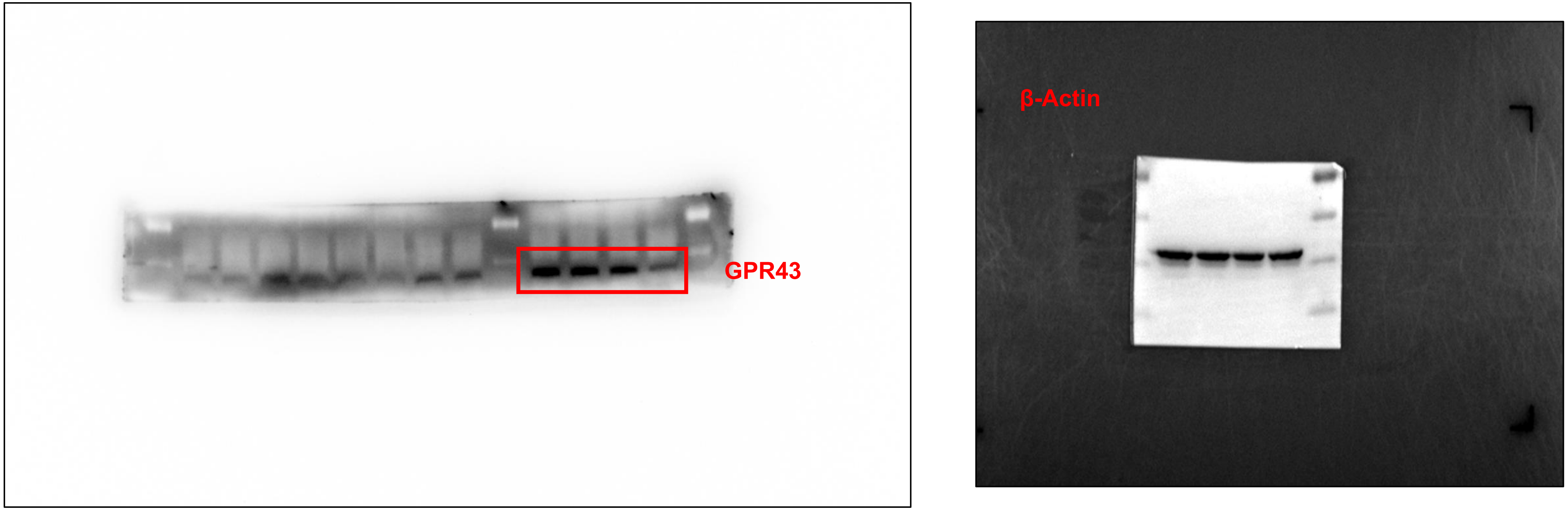

Figure S4A

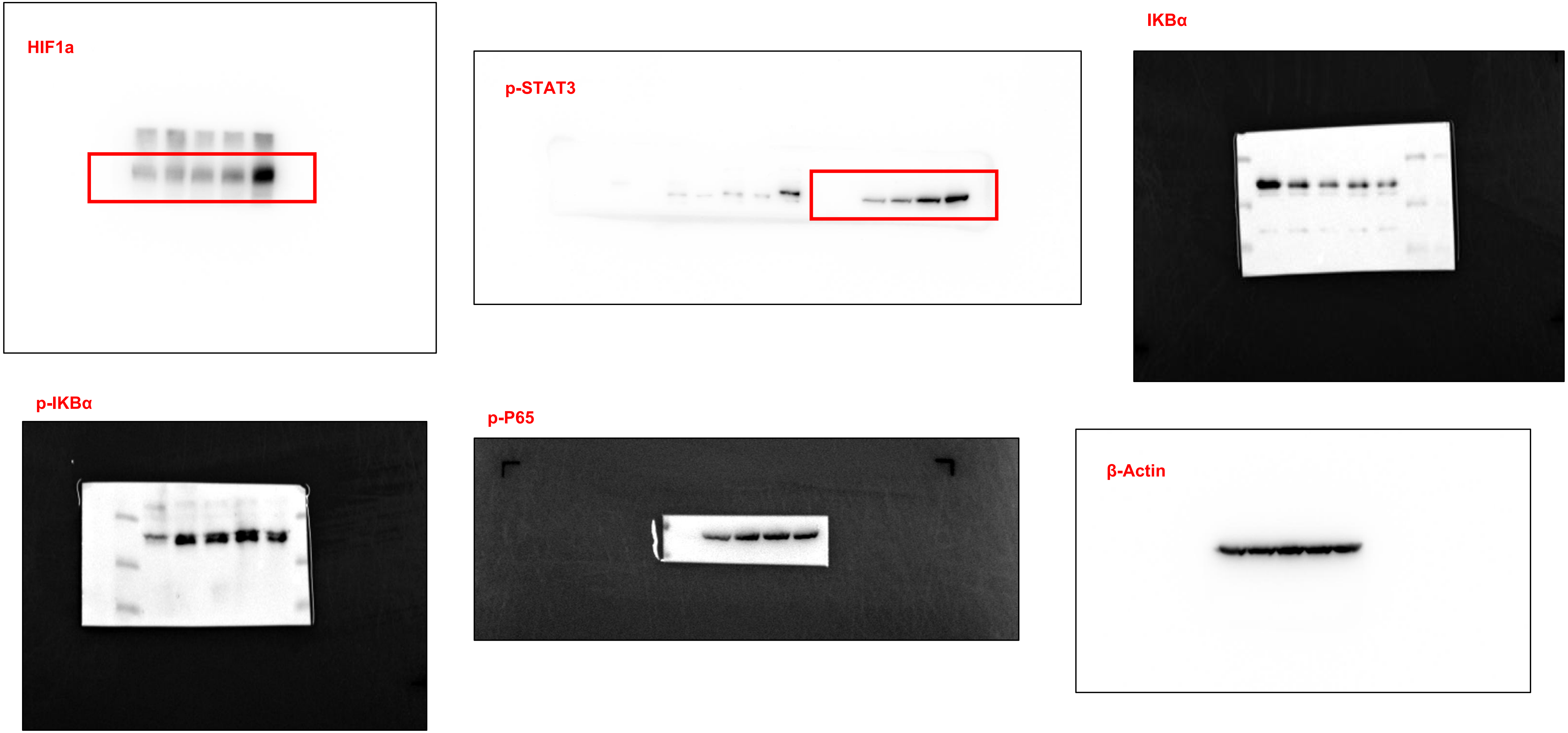

Figure S4B

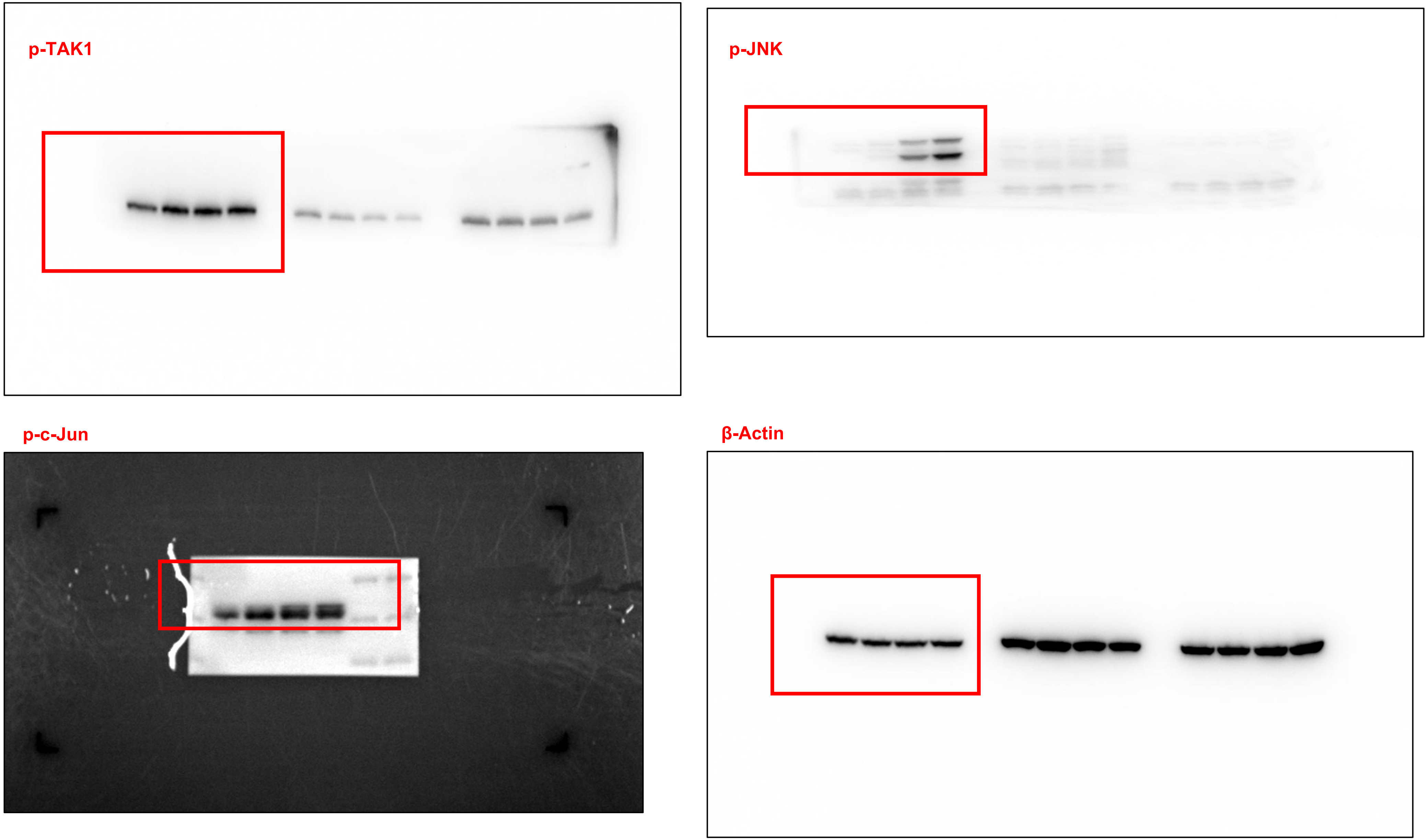

Figure S4C

HIF1a

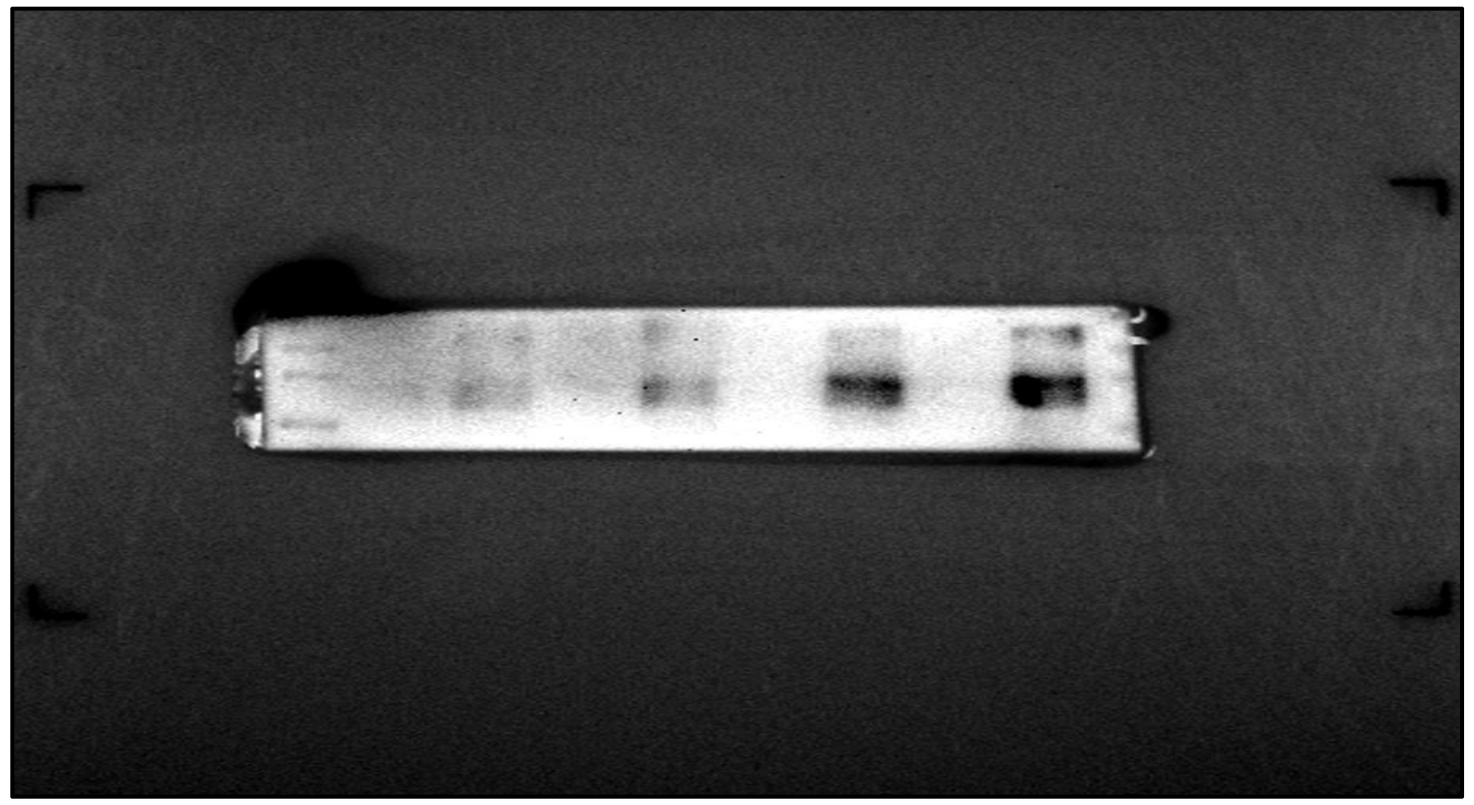

p-STAT3

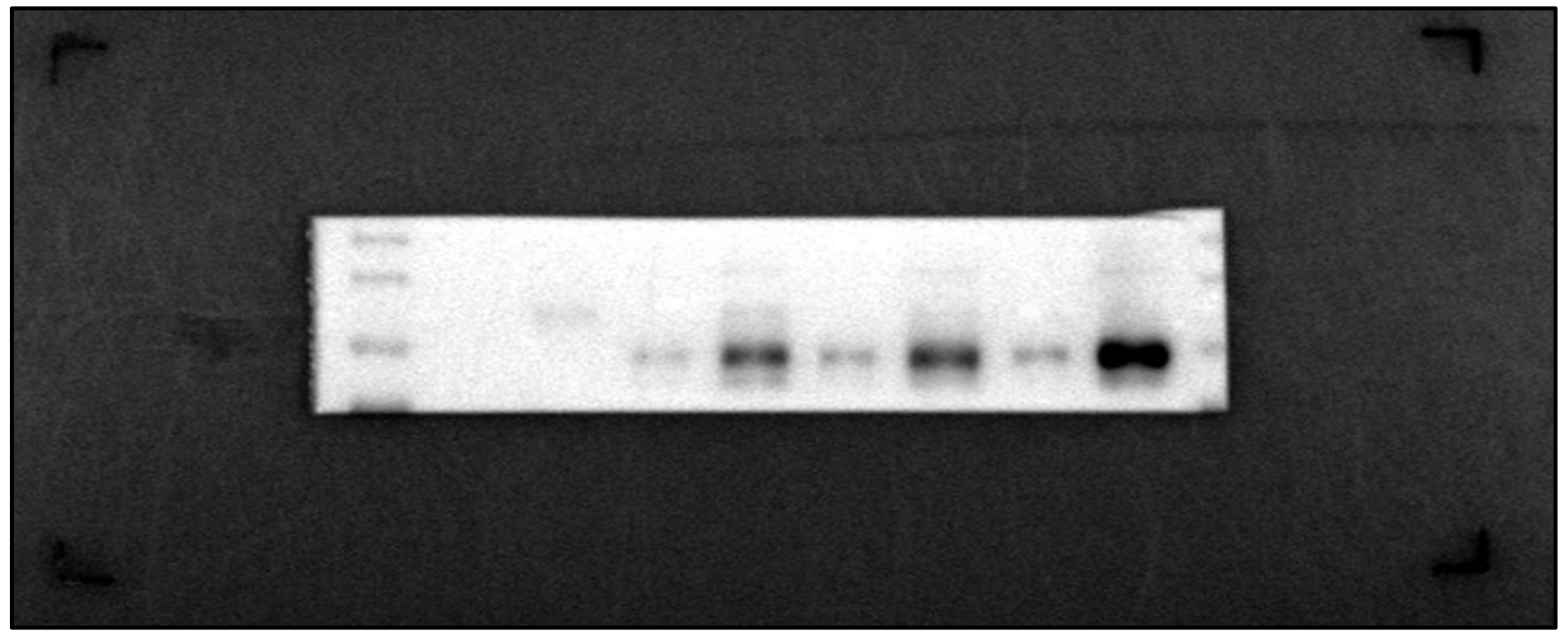

P65

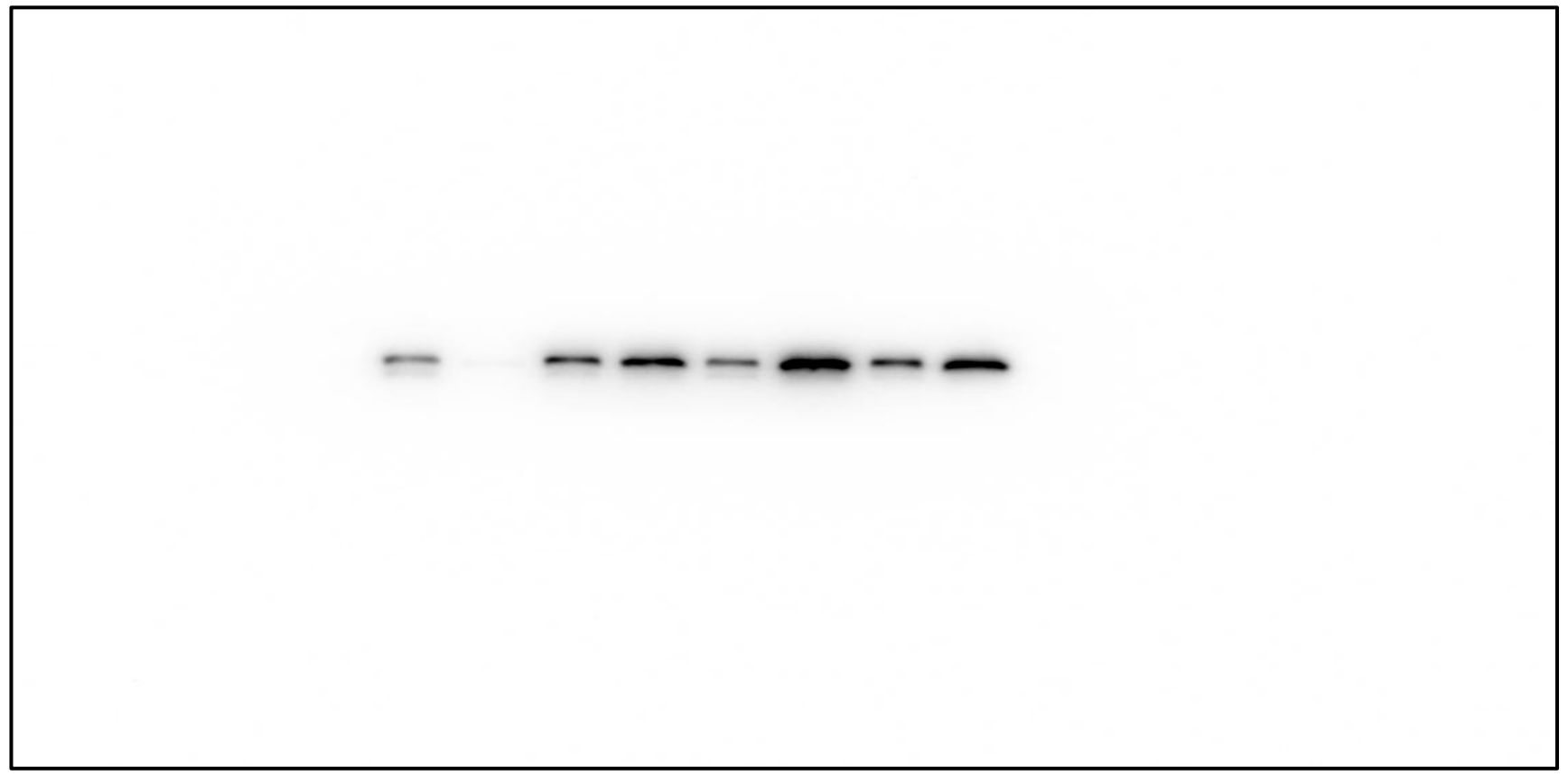

LaminA/C

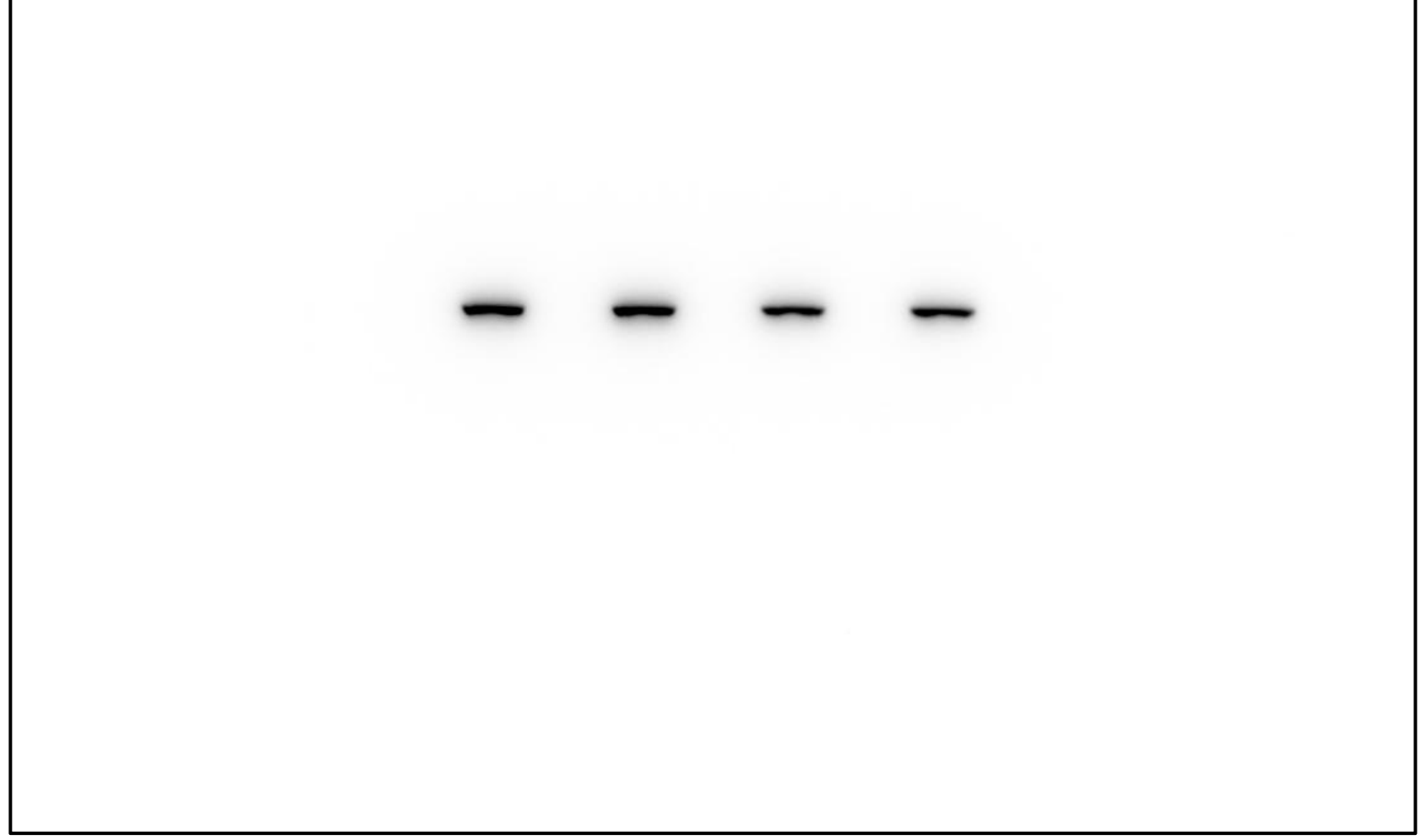

Tublin

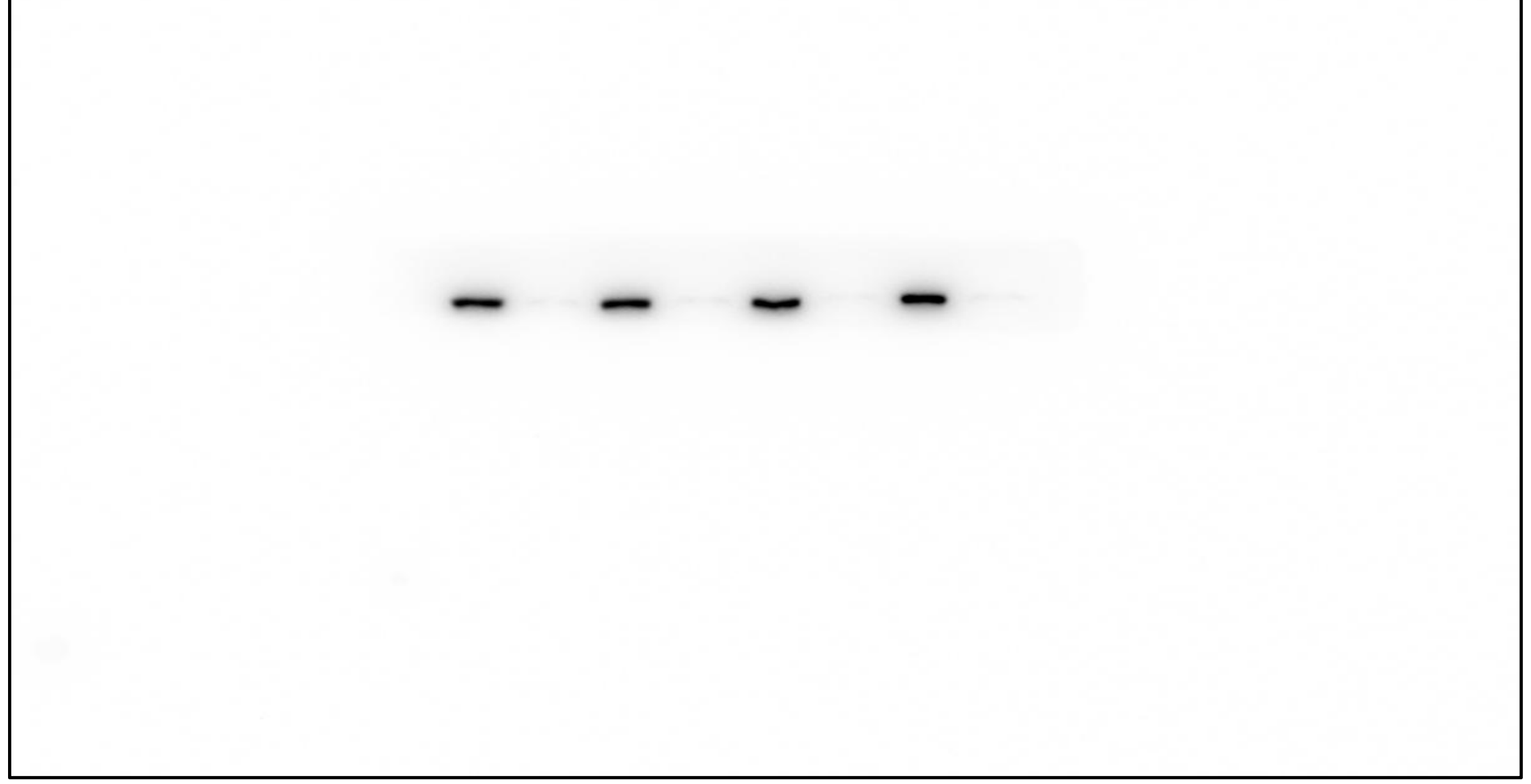

Figure S4D

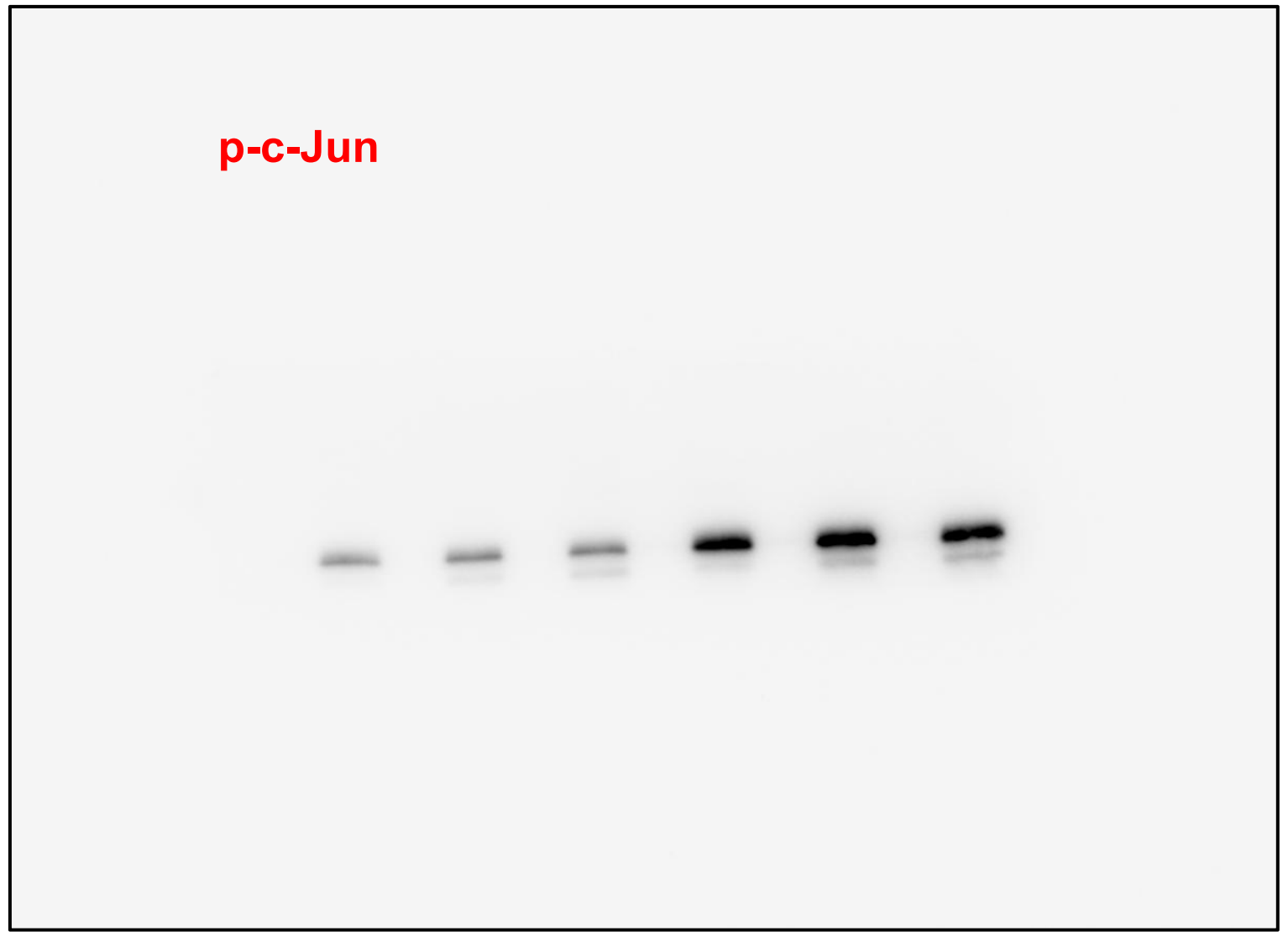

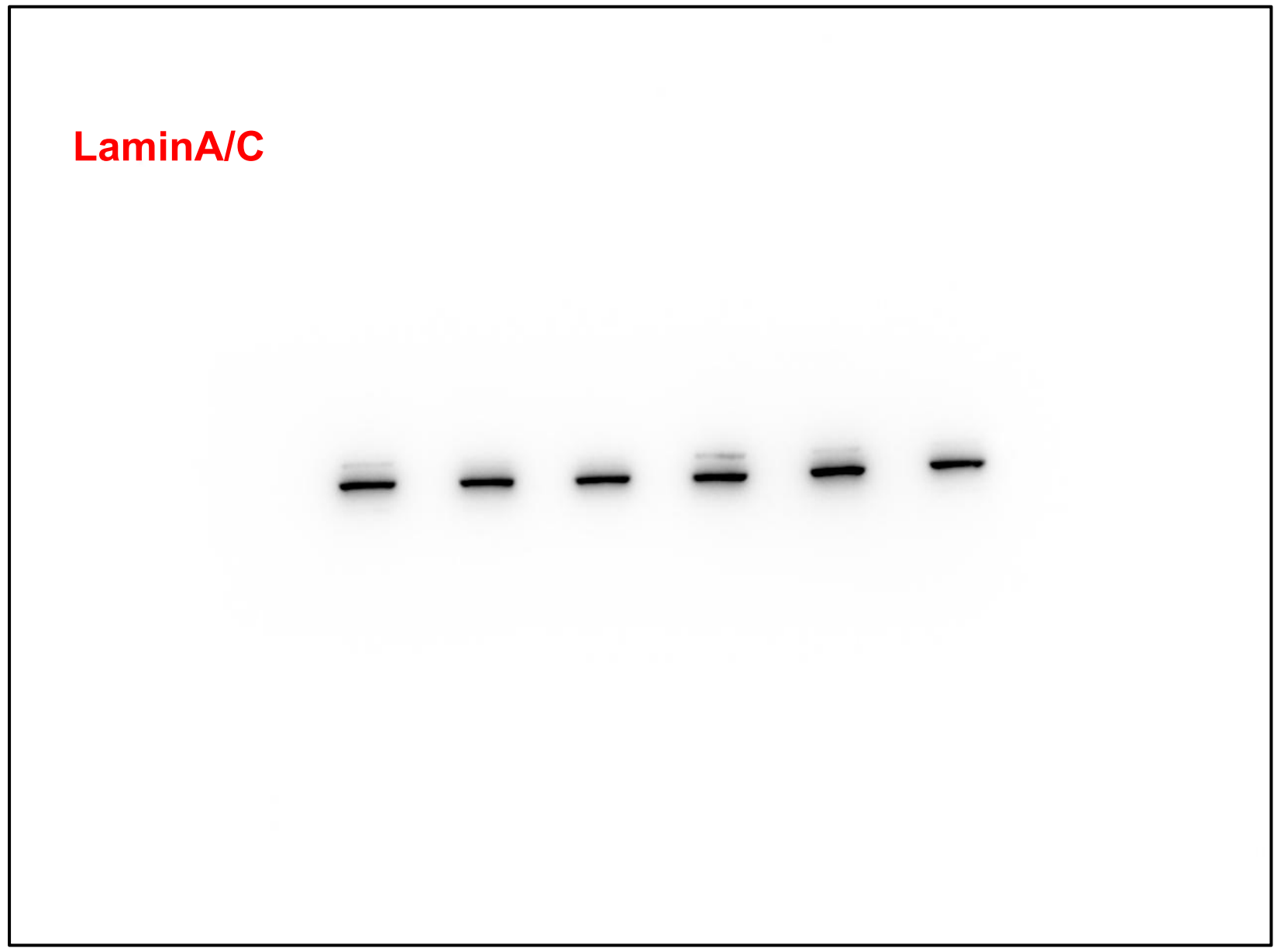

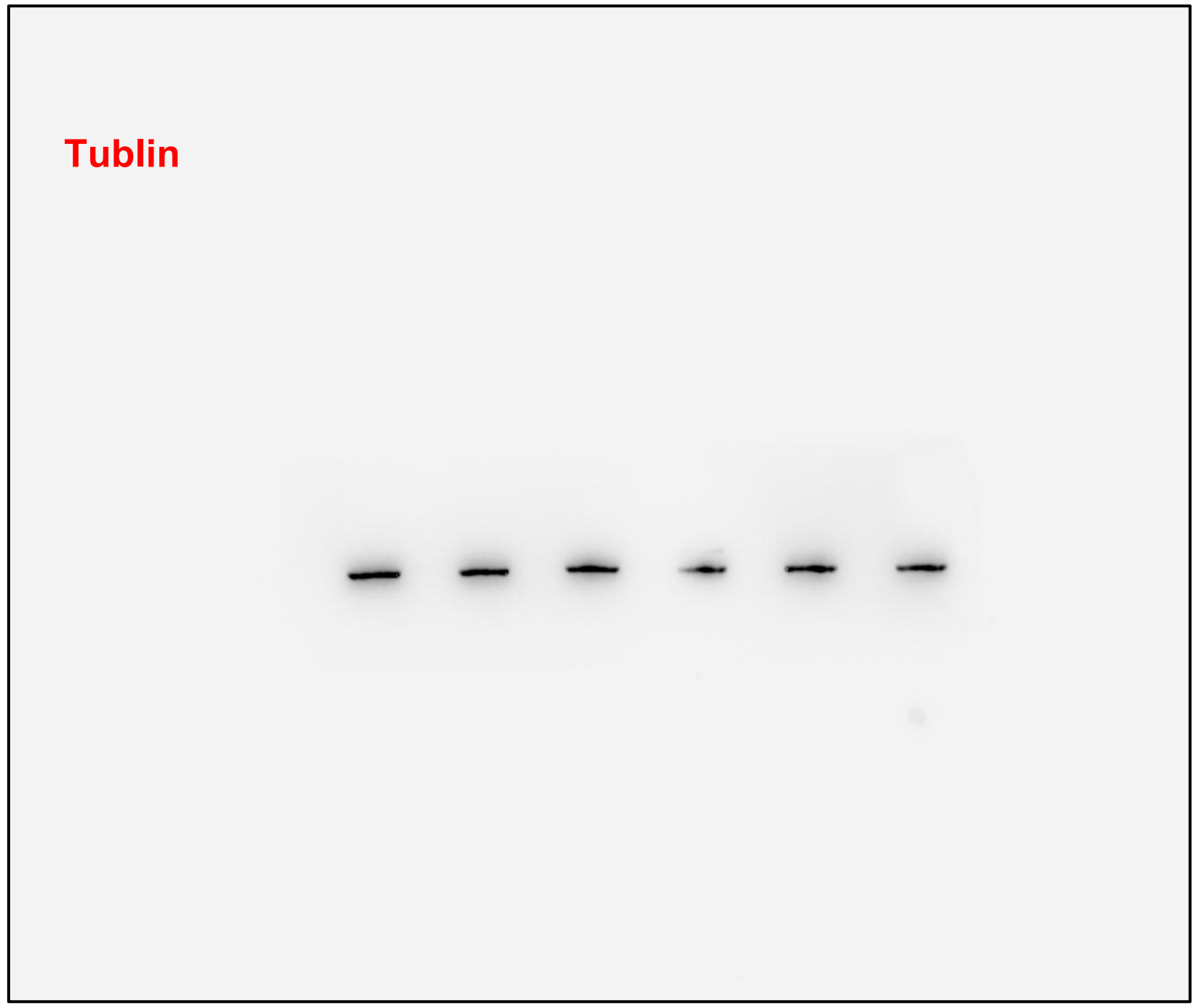

Figure S4E

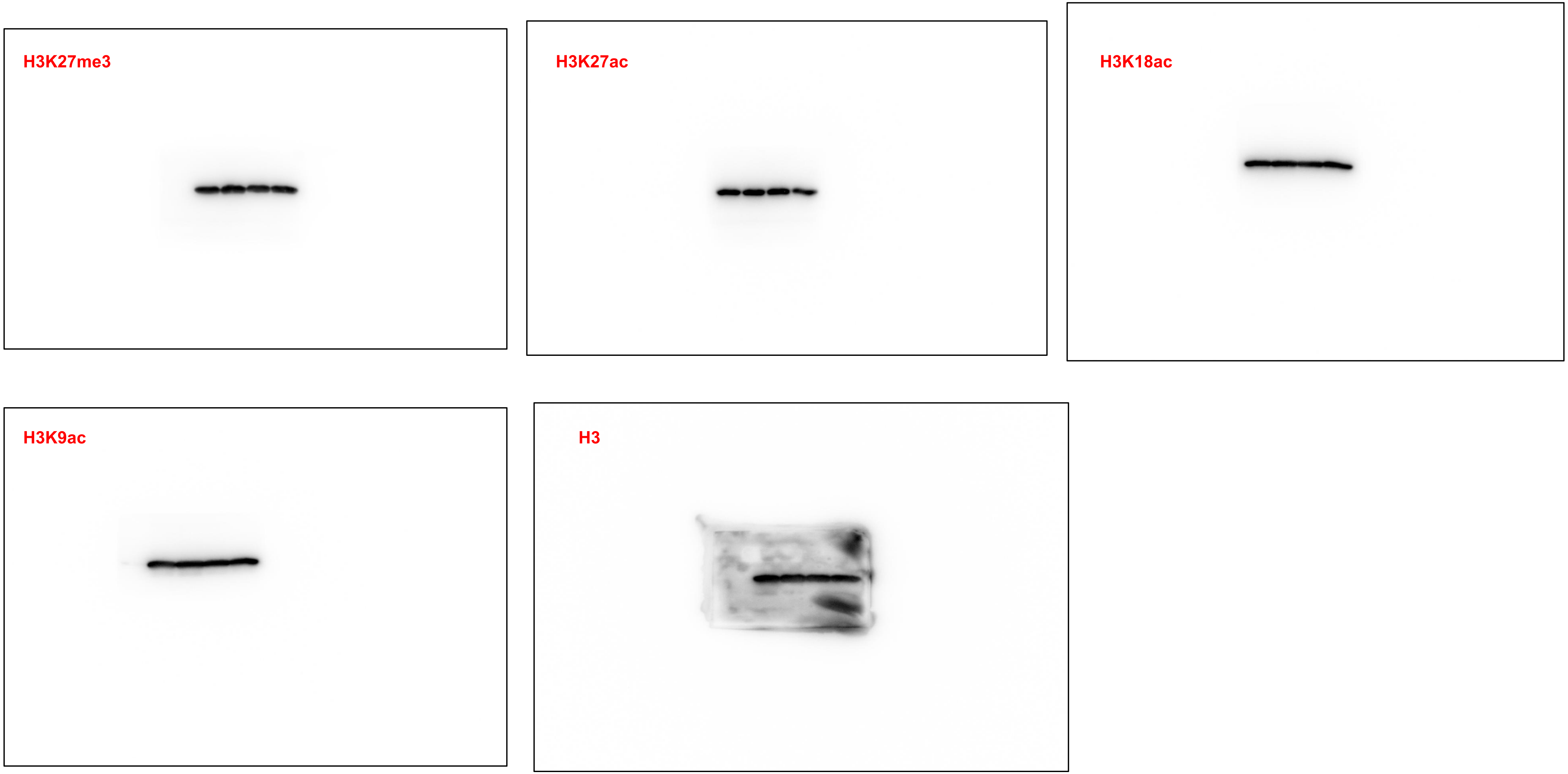

Figure S4G

DNMT1

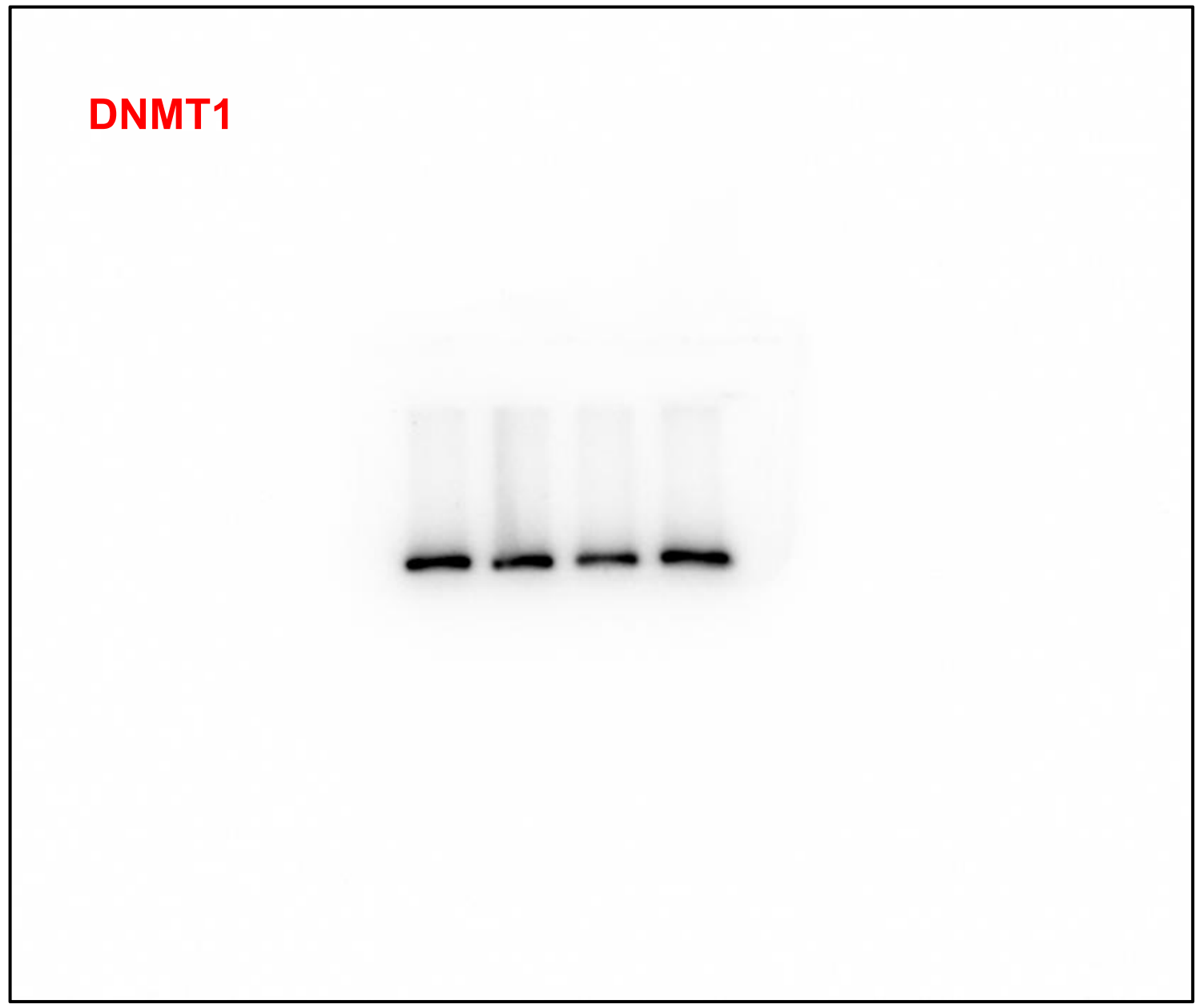

DNMT3A

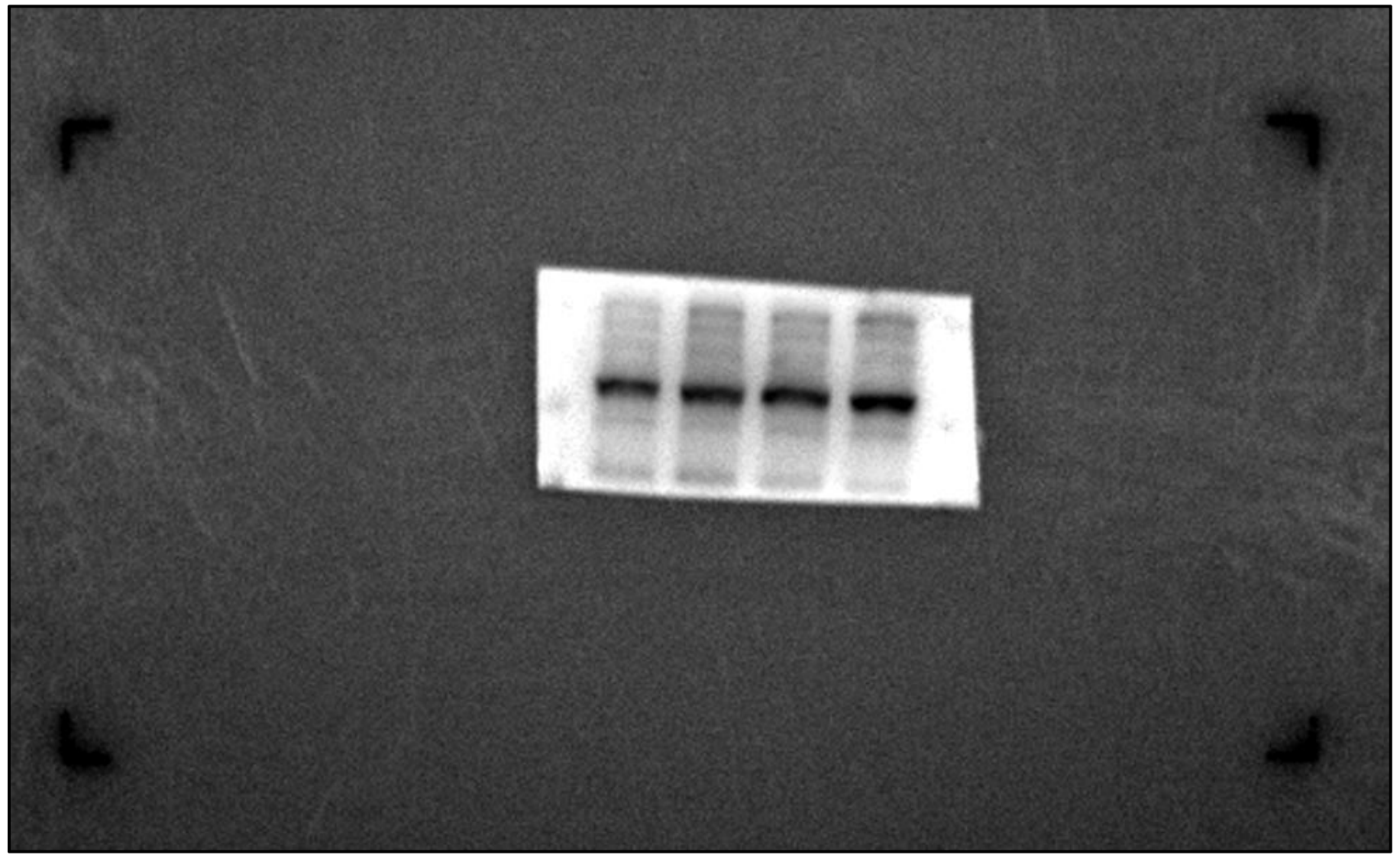

DNMT3B

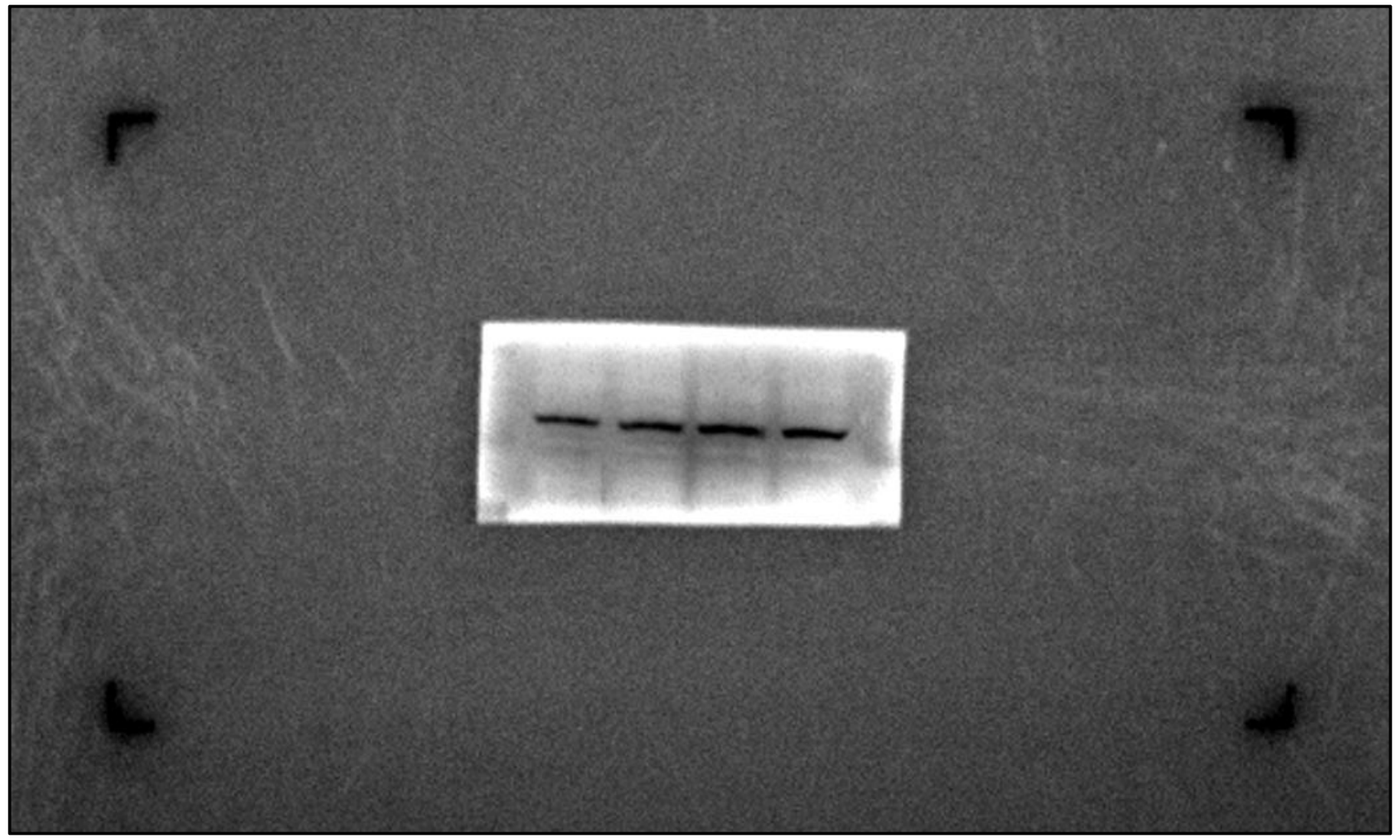

β-Actin

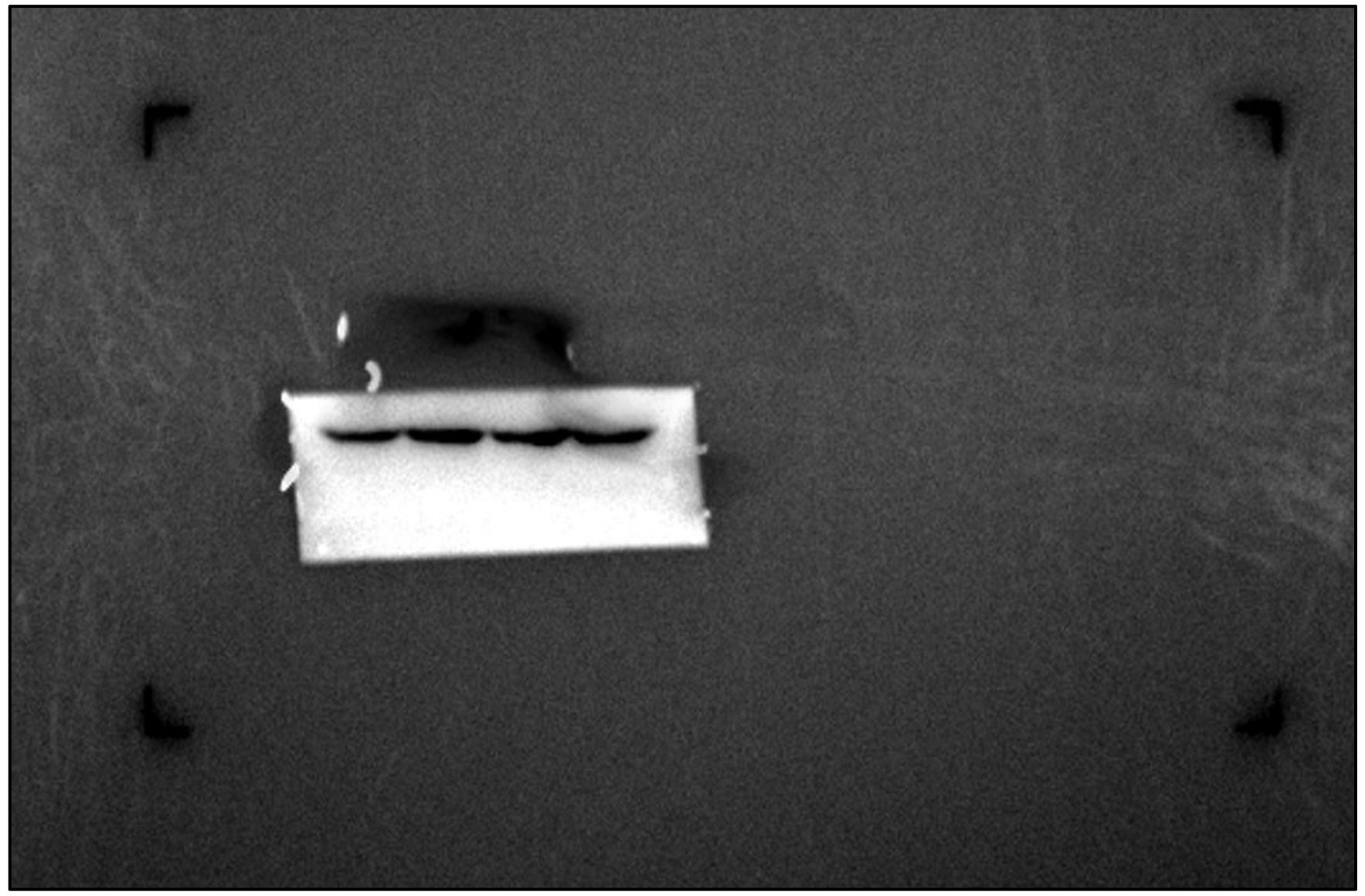

Figure S4H

TET2

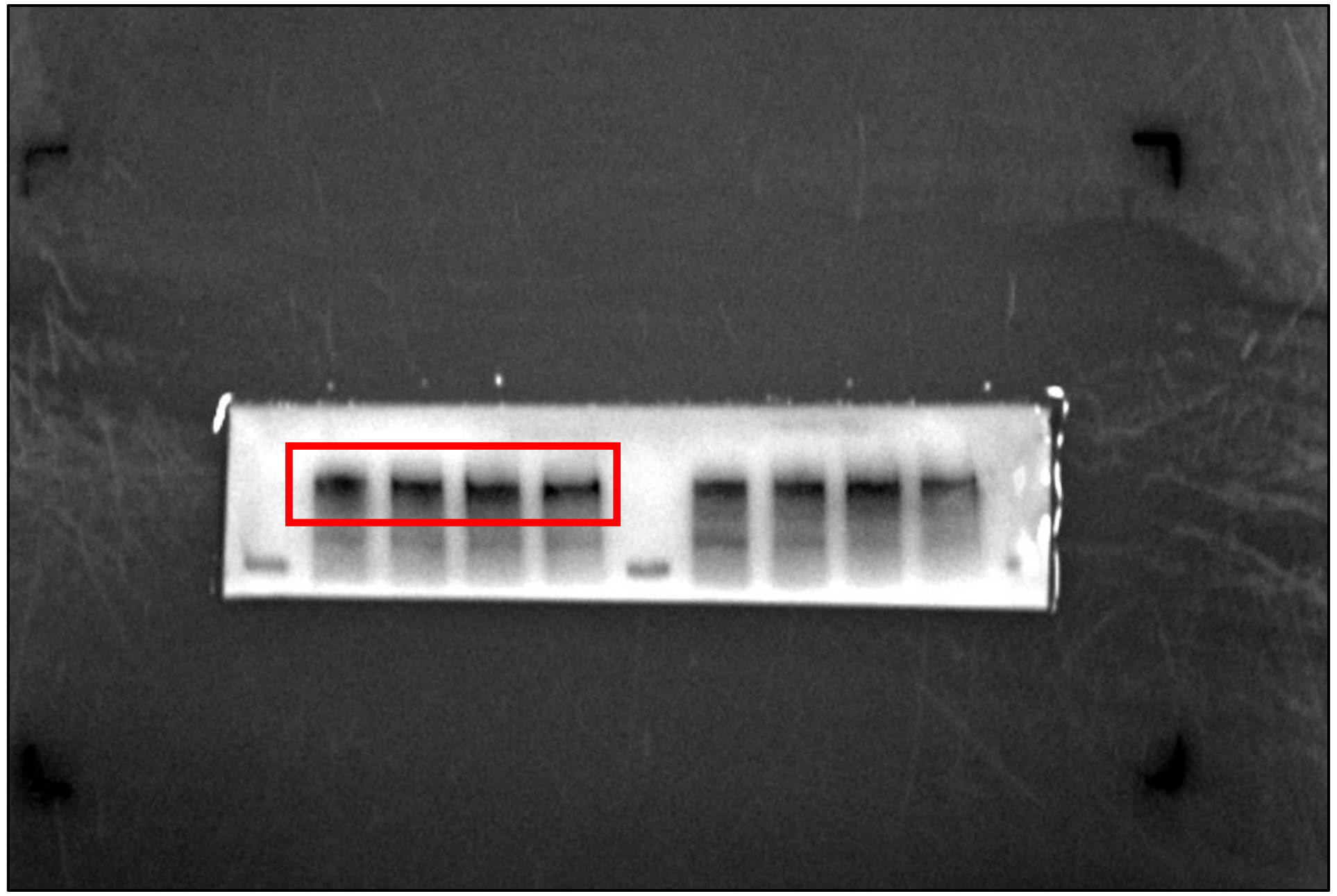

β-Actin

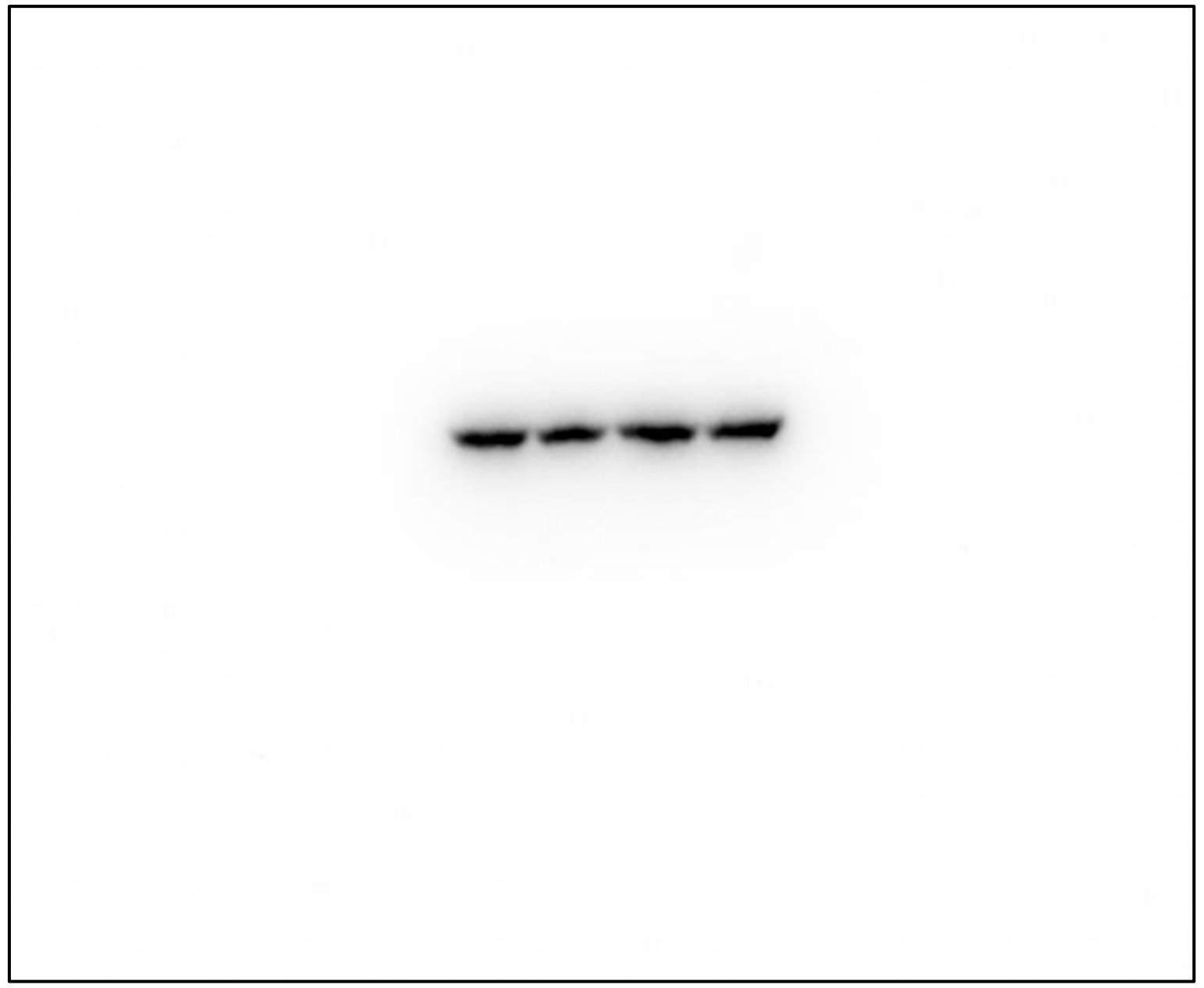

Figure S4I

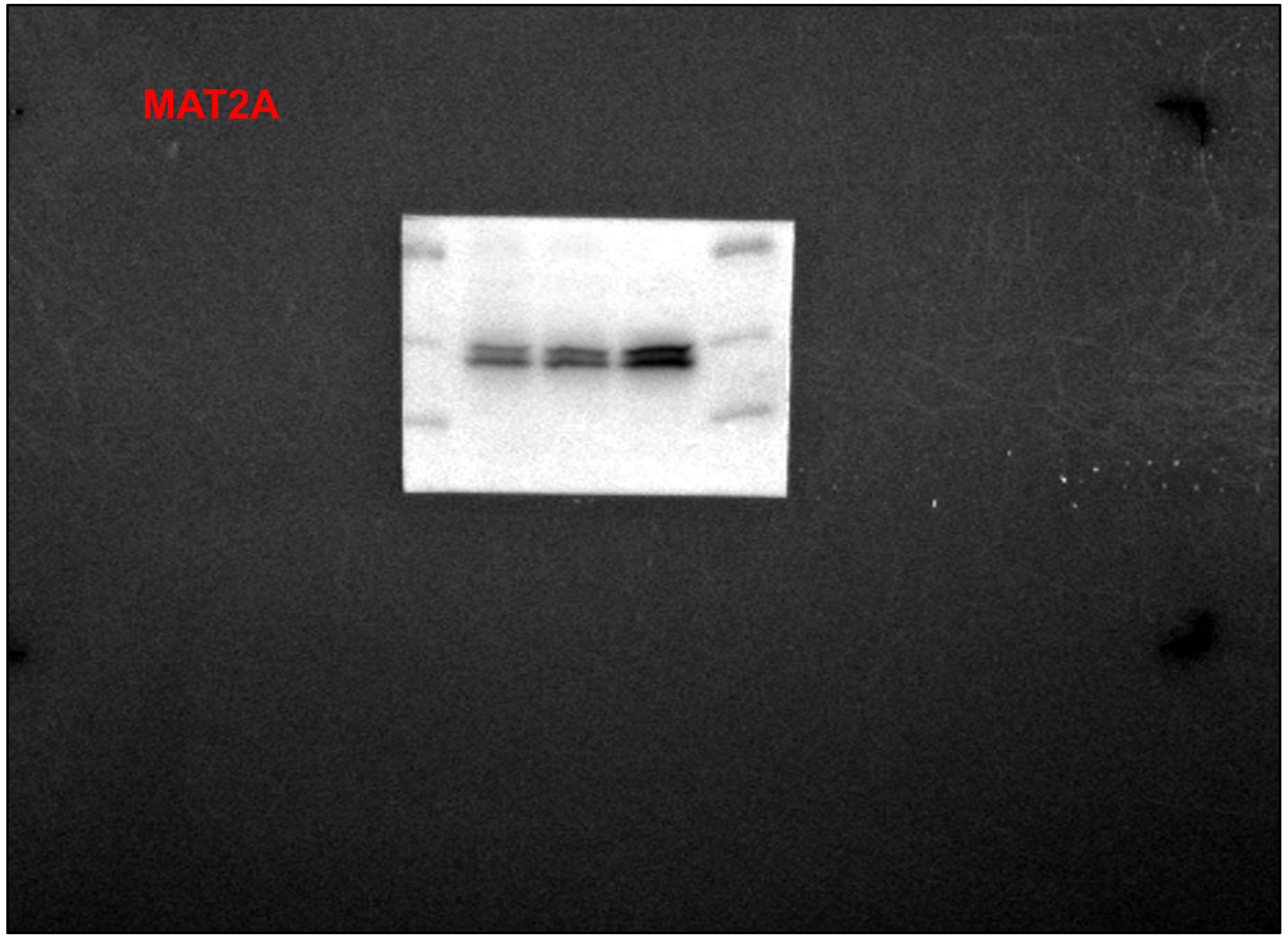

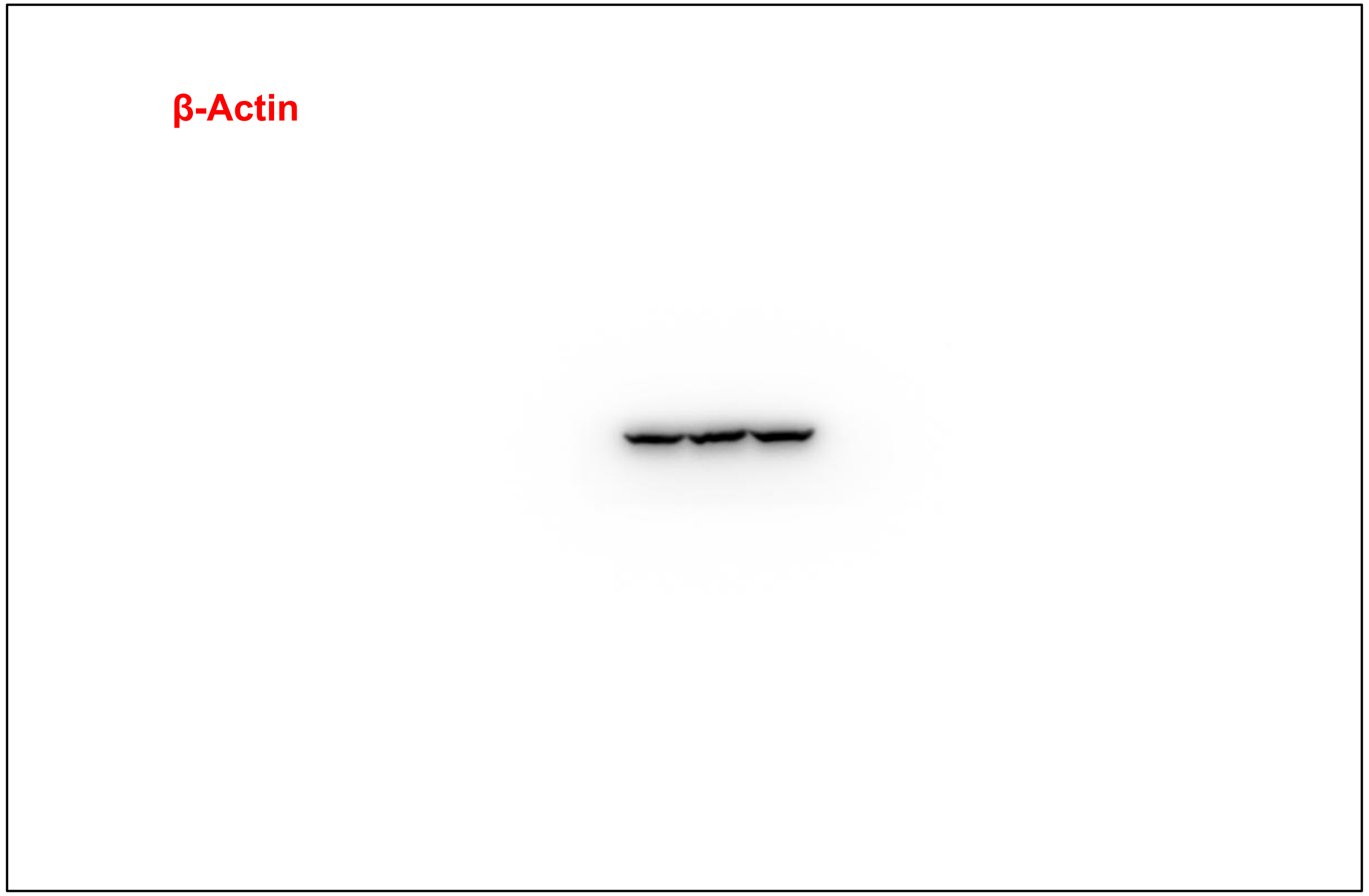

Figure S4J

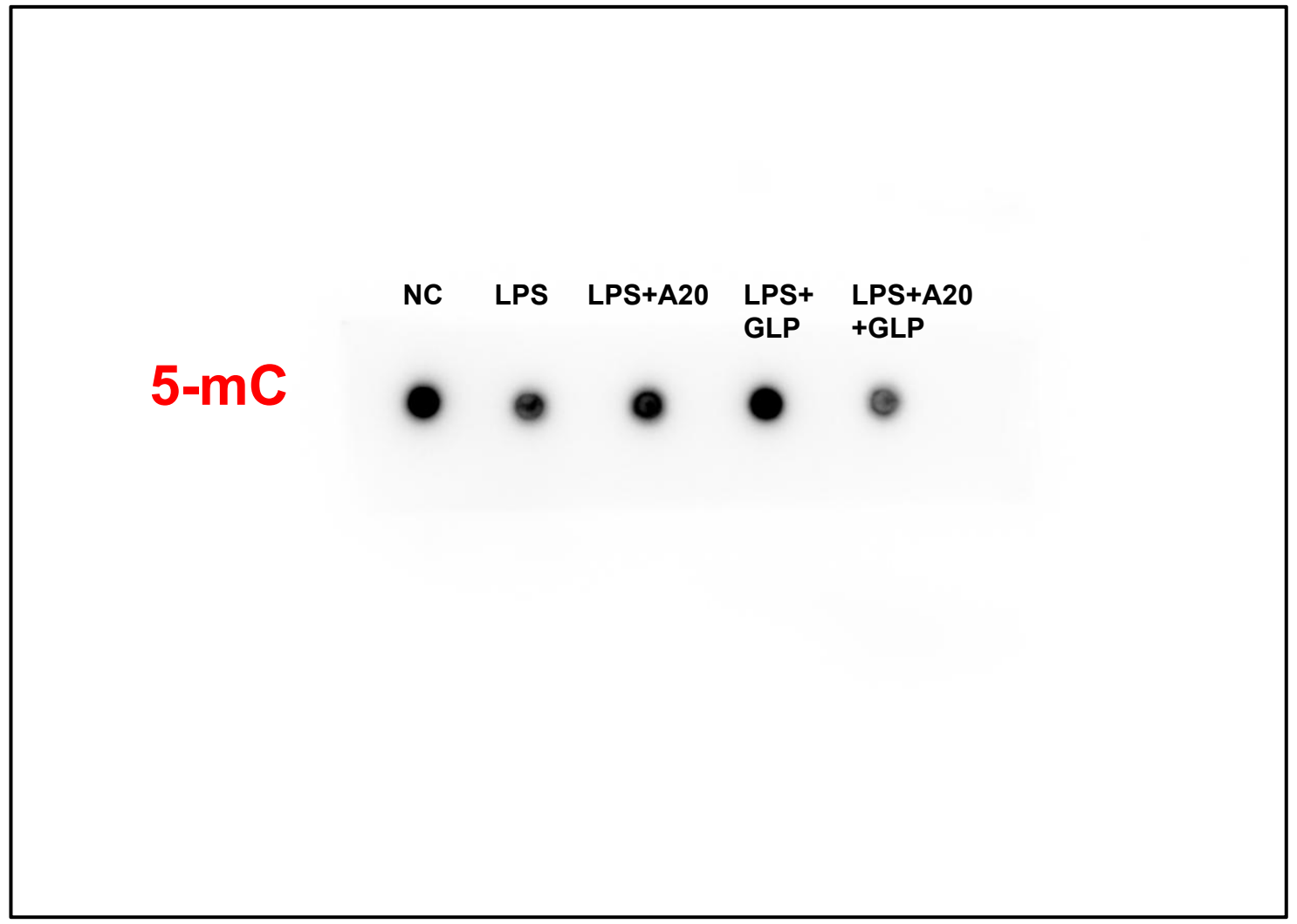
NC
LPS
LPS+A20
LPS+
GLP
LPS+A20
+GLP
5-mC

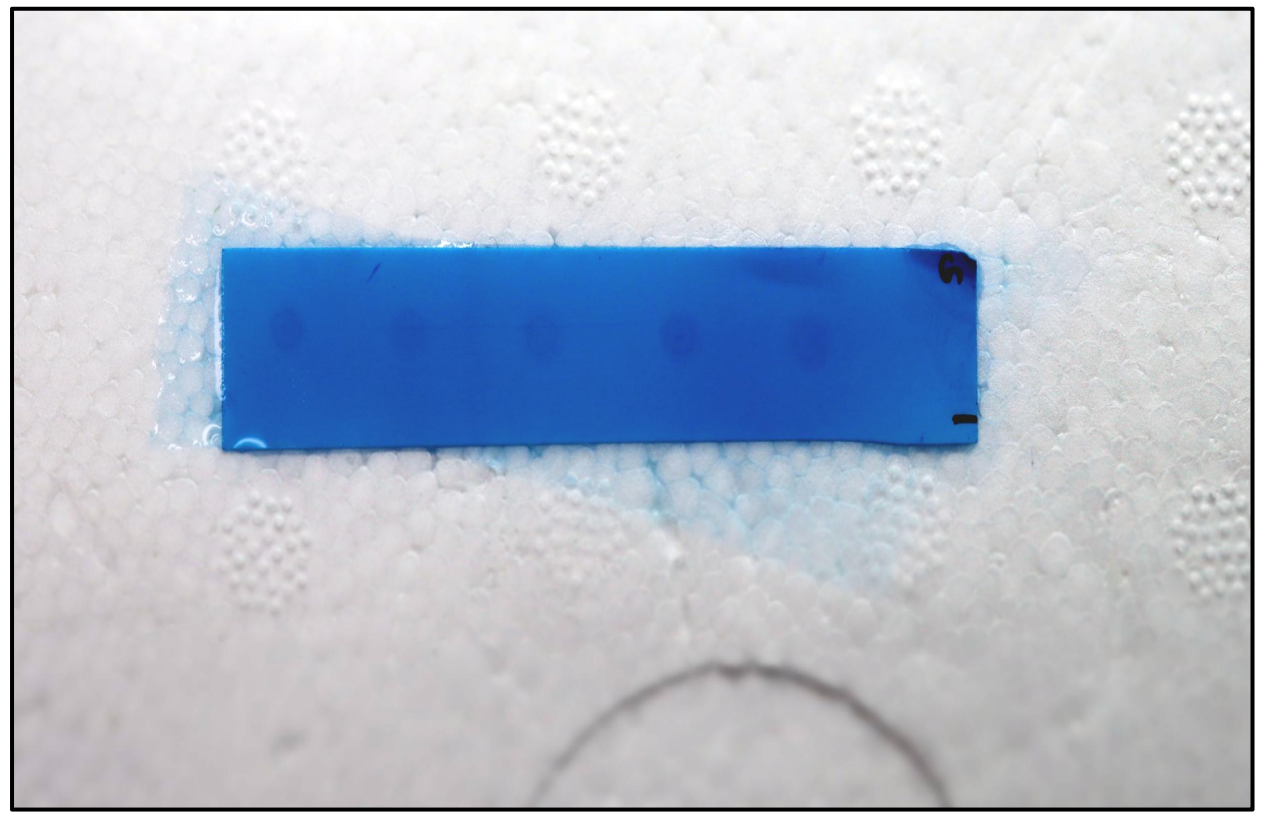

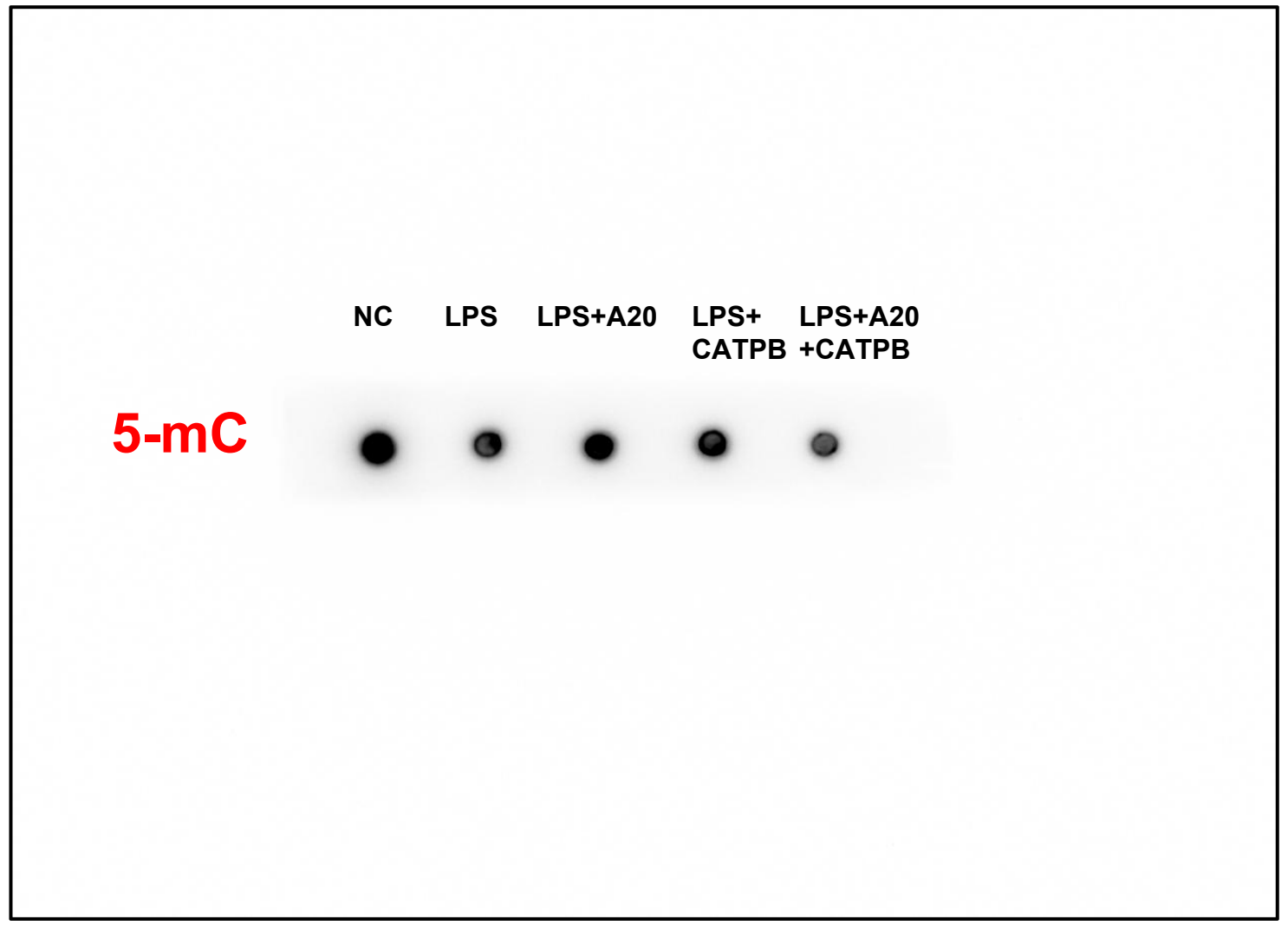
NC
LPS
LPS+A20
LPS+
CATPB
LPS+A20
+CATPB
5-mC

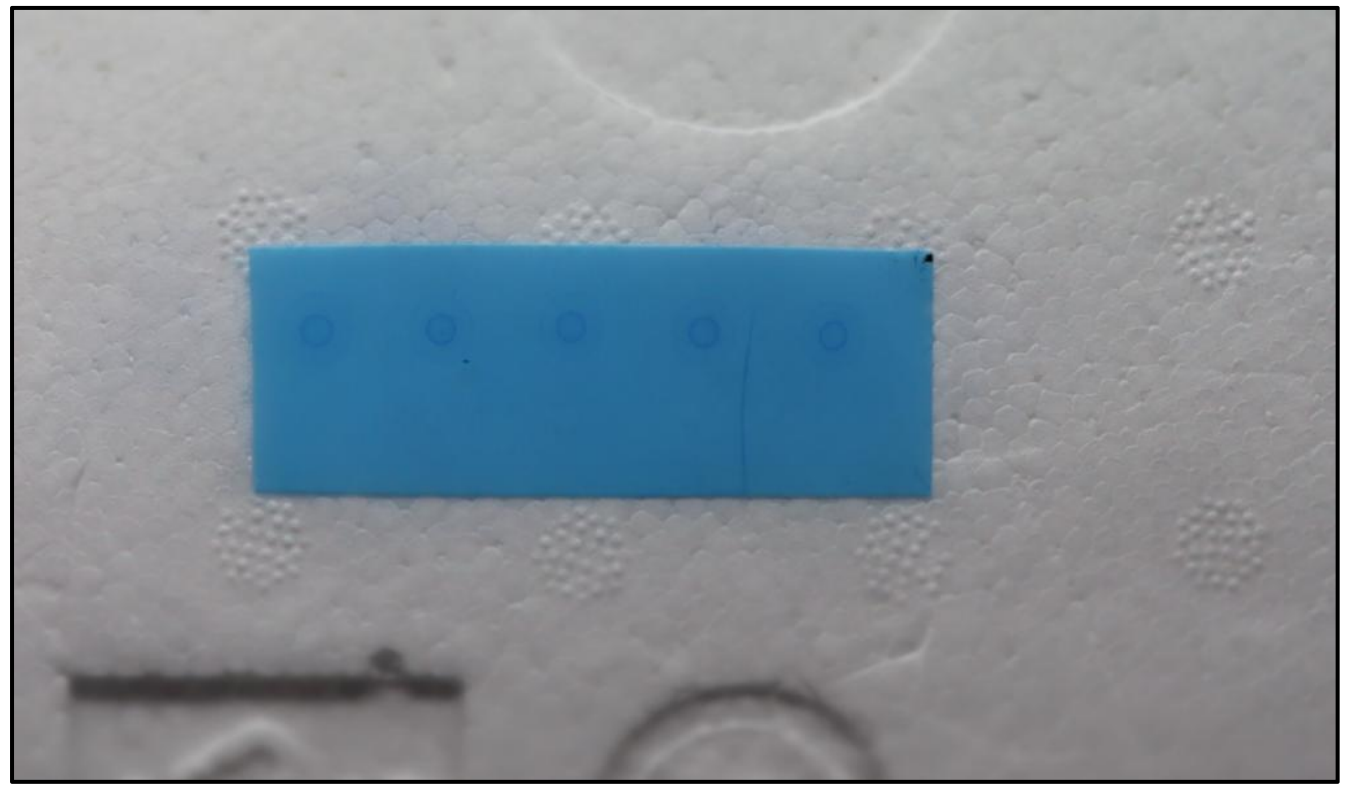

Figure S4K

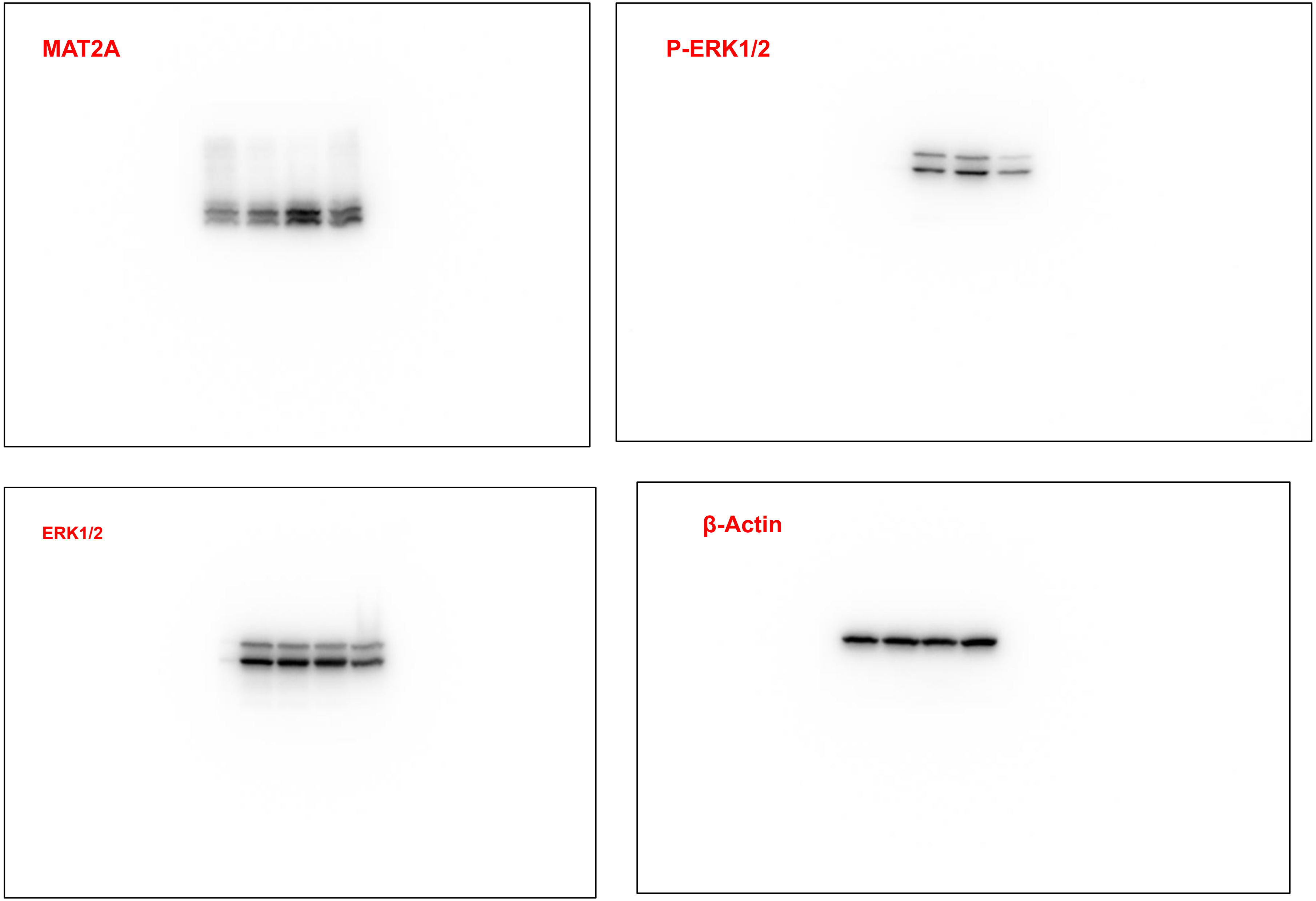

Supplement: Supplementary file 1 [file 12967_2026_8742_MOESM2_ESM.pdf]
