# Supplementary material for: Acetoacetate–GPR43 axis epigenetically silences IL-6/CSF1 to restrict TAMs-driven metastatic lung cancer
Source: J Transl Med. 2026 Aug 3;24:1035. doi: 10.1186/s12967-026-08742-6 (PMC13471439; doi:10.1186/s12967-026-08742-6)
Supplement: Supplementary file 2 [file 12967_2026_8742_MOESM1_ESM.docx]

**Figure S1. Clinical analyses reveal a collapse of acetoacetate homeostasis in metastatic lung cancer, while its restoration suppresses metastatic dissemination with no systemic toxicity**

(A). Mice’s weight was recorded throughout the experiment. (B). Food and water intake were monitored during the entire experiment. (C). Kidney, liver, spleen, and heart tissues were collected and weighed. (D). Representative images of H&E-stained Balb/c mouse liver, kidney, heart, and spleen. (E). Mice’s weight was recorded during the entire experiment. (F). Food and water intake were monitored throughout the experiment. (G). Liver and kidney tissues were collected and weighed. (H). Representative images of H&E pathological staining of C57BL/6 mouse liver, kidney, heart, and spleen. Values are presented as mean ± SD, with * *p* < 0.05, ** *p* < 0.01, *** *p* < 0.001, and n.s. indicating no significance, determined by one-way ANOVA with Dunnett’s post hoc test.

**Figure S2. Acetoacetate suppresses lung metastasis by functioning as an endogenous agonist of GPR43**

(A-B). Molecular modeling of acetoacetate/butyric acid and GPR43(FFAR2) was performed using Gold 5.0 docking software. The merging of the top-ranked acetoacetate/butyric acid pose (shown in green) from the FFAR2 crystal structure (PDB ID: 8T3S) is described. Low-energy binding conformations of acetoacetate/butyric acid (shown in green) bound to the FFAR2 protein were generated by virtual ligand docking. The binding pocket of FFAR2 is represented as a gray ribbon. Amino acid residues and small molecules are depicted as stick models, showing carbon (green, light blue), hydrogen (white), oxygen (red), and nitrogen (blue). (C). Statistical results of Ki-67 staining from randomly selected biological replicates are presented. Values are presented as mean ± SD, with * *p* < 0.05, ** *p* < 0.01, *** *p* < 0.001, and n.s. indicating no significance, determined by one-way ANOVA with Dunnett’s post hoc test.

**Figure S3. A GPR43-dependent metabolic signaling axis connects acetoacetate to the repression of IL-6 and CSF1**

(A). GO-BP enrichment pathways and the representative genes are provided in a detailed list. (B). Peritoneal macrophage (PM) cells were treated with acetoacetate (AC, 10 mM and 20 mM) for 24 hours, with LPS added 6 hours before harvesting. The mRNA levels of IL-6 and CSF1 were quantified by qPCR. (C). THP1 cells were treated with acetoacetate (AC, 10 mM and 20 mM) for 12 hours, with LPS added 6 hours before harvest. The mRNA levels of IL-6 and CSF1 were measured by qPCR. (D). CMT167 cells were treated with acetoacetate (AC, 5 mM, 10 mM, and 20 mM) for 24 hours. The mRNA levels of IL-6 and CSF1 were measured by qPCR. (E). Immunoblot analysis of RAW264.7 si-GPR43 samples. (F). qPCR analysis of RAW264.7 si-GPR43 samples. (G). RAW264.7 cells were transfected with siRNA. At 24 hours post‑transfection, the cells were replated. At 48 hours, they were treated with acetoacetate and LPS for 6 h, then harvested for qPCR. Values are presented as mean ± SEM, with * *p* < 0.05, ** *p* < 0.01, *** *p* < 0.001, and n.s. indicating no significance, determined by one-way ANOVA and Student's *t*-test (F).

**Figure S4. Acetoacetate inhibits IL-6 and CSF1 transcription via GPR43-dependent MAT2A-SAM increase and DNA hypermethylation**

(A-B). RAW264.7 cells were treated with various concentrations of acetoacetate as described and then subjected to Western blot analysis. (C-D). RAW264.7 cells were treated with the indicated concentrations of acetoacetate, followed by nuclear and cytoplasmic separation before Western blot analysis. (E). RAW264.7 cells were treated with various concentrations of acetoacetate as described, and histones were extracted for Western blotting. (F). The 5-mC enrichment levels in IL-6 or CSF1 gene regions were quantified using the MEDIP-qPCR assay. (G-I). Acetoacetate-treated RAW264.7 cells were subjected to Western blot analysis. (J). 5-mC levels in the indicated groups were detected using a dot blot. (K-L). RAW264.7 cells were treated with acetoacetate (AC, 20 mM) or with a combination of AC and SCH (5 µM) for 24 hours, with LPS added 6 hours before harvest. Samples were then analyzed by Western blot and qPCR. Values are presented as mean ± SEM, with * *p* < 0.05, ** *p* < 0.01, *** *p* < 0.001, and n.s. indicating no significance, determined by Student's *t*-test (F) and one-way ANOVA (L).
